# Functionalized Cytisine Squaramides: Synthesis, Structural Elucidation, and Co-Crystallization

**DOI:** 10.3390/molecules31111961

**Published:** 2026-06-04

**Authors:** Anna K. Przybył, Alona Mintianska, Adam Huczyński, Jan Janczak

**Affiliations:** 1 Department of Medical Chemistry, Faculty of Chemistry, Adam Mickiewicz University, Uniwersytetu Poznańskiego 8, 61-614 Poznań, Poland; alona.mintianska@amu.edu.pl (A.M.); adhucz@amu.edu.pl (A.H.); 2 Institute of Low Temperature and Structure Research, Polish Academy of Sciences, Okólna 2 Str., 50-422 Wrocław, Poland

**Keywords:** squaric acid, cytisine derivatives, squaramide, amino acids, crystal structure, co-crystals

## Abstract

Synthesis of bifunctional cytisine–squaramide derivatives bearing a single amino acid moiety has revealed an unexpected and intriguing chemical challenge. During modification of cytisine squaramates with α-amino acids, base-sensitive amido esters readily underwent hydrolysis, forming poorly soluble amido-acid side products that resisted standard purification and initially obscured their identity. Persistent observation of these elusive precipitates prompted a deliberate co-crystallization approach, which unambiguously revealed their supramolecular nature using single-crystal X-ray diffraction. With this insight, optimized purification strategies allowed isolation of analytically pure Cyt-SQ-OH and its derivatives, which were characterized by complementary spectroscopic techniques, X-ray crystallography and computational studies. Furthermore, the DFT-optimized parameters of all compounds were determined, providing additional insight into their structural and electronic properties. This work highlights the interplay between reactivity, solubility, and supramolecular assembly in cytisine–squaramide-amino acid hybrids, providing a robust platform for future exploration of multifunctional conjugates with potential applications in medicinal chemistry, molecular recognition, and materials science.

## 1. Introduction

Biological properties of small organic molecules are determined by the nature, spatial arrangement, and electronic characteristics of their functional groups, commonly referred to as pharmacophores. These elements govern molecular recognition and modulate interactions with enzymes, receptors, and other biological targets. Steric effects, polarity, solubility, and electron density collectively shape molecular behavior in chemical and biological environments [1,2], making targeted functional group modification a cornerstone of modern medicinal and supramolecular chemistry.

Naturally derived compounds represent a privileged class of scaffolds due to their structural diversity and inherent biological relevance. Many exhibit antimicrobial, antioxidant, anticancer, antiviral, and anti-inflammatory activities, making them attractive templates for further functionalization. Among them, quinolizidine alkaloids have received sustained attention for their rigid polycyclic frameworks. A prominent example is (-)-cytisine (CYT), a tricyclic alkaloid used as a partial agonist of nicotinic acetylcholine receptors (nAChRs) in smoking cessation therapy [3]. Beyond this established application, CYT modulates neurotransmission, glucose homeostasis, and tumor cell proliferation [4], highlighting its potential as a biologically validated molecular scaffold. Crystallographic studies of nAChRs (nicotinic acetylcholine receptors) have revealed precise interactions between CYT and key receptor residues [5,6,7], emphasizing the importance of functional group positioning in molecular recognition.

In parallel, squaric acid derivatives, particularly squaramides (SQA), have emerged as versatile motifs in medicinal chemistry, catalysis, and supramolecular systems. Squaramides are defined by a rigid cyclobutenedione core, electron-withdrawing character, and planar geometry, enabling directional hydrogen bonding and metal coordination. As a result, SA derivatives, including squarates, squaramates, and squaramides, can act as bio-isosteres of carboxylic acids, amino acids, phosphate, and sulfate esters [8], underlining their broad utility. Squaraine-type derivatives further expand the scope of SA chemistry, offering exceptional photophysical properties and structural rigidity, which have been exploited in bioimaging and stereoselective catalysis [9,10].

The functional versatility of squaramides spans medicinal chemistry, from organocatalysis to anticancer activity [9,10,11,12,13,14] and inhibition of pathogenic mycobacteria, where squaramides have recently emerged as ATP synthase inhibitors with micromolar activity against *M. tuberculosis* and improved tolerability [15]. Their conformational dynamics, including rotamerism, influence recognition and binding properties [16], inspiring the development of squaramide-based receptors [17]. Sustained interest in squaramides continues to drive advances in their synthetic design and biological evaluation, including recent studies demonstrating the bioisosteric potential of the cyclobutenedione moiety targeting cannabinoid receptors [18]. Incorporation of amino acids into squaramide frameworks enhances structural diversity and functionality, enabling antioxidant, metal-binding, and anion-recognition properties [19,20,21,22,23,24,25,26,27,28]. Recent studies demonstrate that amino acid functionalization or dipeptide motifs can enhance antioxidant activity and impede amyloid-β aggregation [26]. The combination of CYT with amino acid–functionalized squaramides offers a unique opportunity to integrate complementary functional elements: the hydrogen-bonding and metal-binding capacity of squaramides, the structural and antioxidant versatility of amino acids, and the neuroactive, metal-coordinating properties of CYT. Such hybrid molecules are well-positioned for multifunctional applications, including medicinal chemistry, bioimaging, and supramolecular recognition.

In this paper, we report the design, synthesis, and comprehensive structural characterization of novel cytisine–squaramide derivatives. By elucidating their molecular structure, conformational behavior, and solid-state organization through spectroscopic methods and single-crystal X-ray diffraction, we aim to establish a foundation for the rational development of multifunctional hybrid scaffolds, paving the way for future studies of their bioactivity and functional applications.

## 2. Results and Discussion

### 2.1. Chemistry

In this study, the synthesis of bifunctional squaramide derivatives bearing a single amino acid moiety was undertaken. Modification of cytisine squaramate CytSQOMe (**1**) was initiated by introducing the simplest α-amino acid, α-alanine, followed by an analogous approach employing α-phenylalanine. Although the target products were successfully obtained, the synthetic route was accompanied by several challenges related to side-product formation and purification. These difficulties arose primarily from base-induced hydrolysis of the initially formed amido esters (**1** and **2**), leading to the corresponding squaramide acid CytSQOH (**3**), as well as from the limited solubility of the resulting compounds in commonly used organic solvents.

**Scheme 1:**
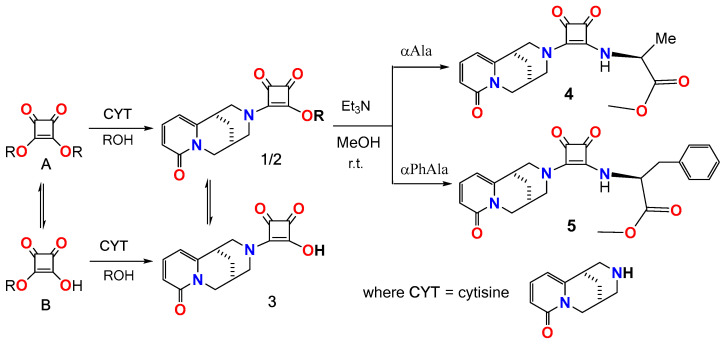
Equilibrium between diesters-SQ (**A**) and acid-SQ-ester (**B**), where R = Me (**1**) or R = Et (**2**) and synthesis of bifunctional squaramides **4** and **5**.

Recent investigations [29,30] expanded this concept to the synthesis of new amino acid-derived cytisine squaramides (**4** and **5**, Scheme 1). The relevant starting materials had already been employed as substrates for bifunctional squaramides [30]; however, the structures of the amido ester CytSQOMe (**1**/**2**) and the amido-acid CytSQOH (**3**) (Scheme 1) were not elucidated at that stage due to persistent difficulties in obtaining single crystals suitable for X-ray diffraction analysis.

Subsequent studies of amino acid derivatives of cytisine [31] encouraged further development of this approach through incorporation of a squaric acid linker. It was observed that the amido ester CytSQ-OMe (**1**), when dissolved in methanol in the presence of even trace amounts of CytSQOH (**3**), readily undergoes an acid–ester equilibrium. As a consequence, a poorly soluble white precipitate is formed, which had previously been regarded as an undefined side product and routinely removed during work-up. As the study progressed, it became evident that this behavior was intrinsic to the chemistry of the squaric acid ester precursor itself. Even the commercial 3,4-dimethoxycyclobutene-1,2-dione exists as an equilibrium mixture of the diester (**A**) and half-ester (**B**) forms (Scheme 1). This equilibrium was confirmed by TLC analysis, which showed a characteristic paired signal of both species, with compound **B** displaying lower polarity than diester **A**. The presence of the half-ester (**B**) predisposes the system to partial ester hydrolysis under mild conditions, promoting the formation of amido-squaramide acid derivatives.

In contrast to squaric acid, which exhibits low solubility in organic solvents and requires thermal activation to react with cytisine, the diester dimethyl squarate (**A**) displays enhanced reactivity due to electron-donating methoxy substituents. While this reactivity facilitates nucleophilic substitution and formation of the desired squaramate CytSQ-OMe (**1**), it simultaneously promotes formation of the amido-acid side product CytSQOH (**3**). Consequently, reactions designed to yield compound **1** often produced mixtures of ester **1** and acid **3**, complicating isolation and obscuring the origin of the insoluble material. These challenges were particularly evident during the purification of products **4** and **5** (Scheme 1). Attempts to isolate compound **1** via liquid–liquid extraction under basic conditions (pH = 11–14) resulted in extensive ester hydrolysis and transfer of squaramide-derived species, including **3** and amino acid fragments, into the aqueous phase. Under neutral conditions, both unreacted substrates and the target compound remained in the organic phase, preventing effective separation. Flash column chromatography emerged as the most reliable purification method; however, recrystallization from hot methanol or ethanol repeatedly led to partial hydrolysis upon prolonged standing.

A turning point in the study was the recurrent appearance of a poorly soluble white precipitate during work-up. Initially dismissed as an inert by-product, this material consistently resisted further reaction and purification, effectively halting subsequent synthetic transformations. Its persistence prompted a reassessment of its origin. Detailed analysis has revealed that the precipitate arises from spontaneous association of the amido ester CytSQ-OMe (**1**) with the amido-acid CytSQOH (**3**), generated through partial ester hydrolysis. To verify this hypothesis, co-crystal formation was deliberately induced by mixing equimolar amounts (1:1) of compounds **1** and **3** in DMSO. This controlled experiment yielded a well-defined co-crystal **6**, and a related co-crystal **7** was obtained upon heating of a 1:1 mixture of **2** and **3** in DMSO. Single-crystal X-ray diffraction unambiguously confirmed that the initially problematic precipitate corresponds to a stable supramolecular assembly composed of both ester and acid components. Deliberate co-crystallization not only clarified the identity of the side product but also resolved the origin of the poorly soluble and apparently non-reactive material encountered during synthesis. With this insight, subsequent synthetic efforts were focused on isolating the methanol-soluble amido ester CytSQ-OMe (**1**). Optimization of purification conditions, specifically, flash column chromatography combined with complete elimination of methanol from the eluent, enabled isolation of analytically pure compound **1**. Recrystallization from acetonitrile afforded crystals suitable for single-crystal X-ray diffraction, enabling the structural characterization of compound **1** and conjugates **4** and **5**. Compound **3** was partially crystallized from isopropanol, while a second crop obtained from DMSO yielded the solvate **3·DMSO**. Co-crystals **6** and **7** were obtained by recrystallization from DMSO.

### 2.2. X-Ray Structure Characterization

The crystal data of the investigated compounds and the final refinement parameters are summarized in Table 1.

#### 2.2.1. Squaramide of **3·DMSO**

**Figure 1:**
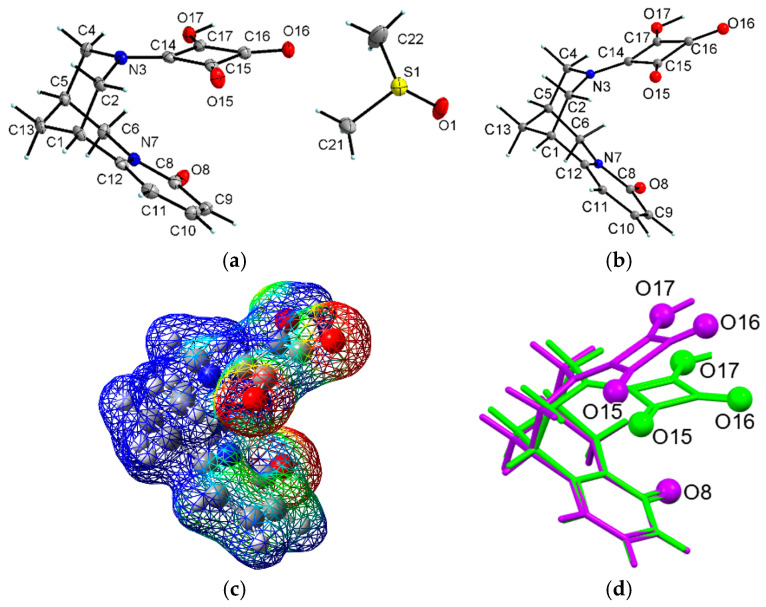
(**a**) Asymmetric unit of **3·DMSO** with atom labeling scheme. Displacement ellipsoids are shown at the 50% probability level, H atoms with arbitrary radii, (**b**) DFT optimized structure of amido-acid CytSQOH and (**c**) 3D molecular electrostatic potential mapped onto the total electron density isosurface (0.008 eÅ^−3^), red and blue in the EP represent values between −0.05 and 0.05 eÅ^−1^, and (**d**) comparison of the conformation of amido-acid CytSQOH molecule: DFT—purple and X-ray—green.

Amido-acid CytSQOH (**3**) crystallizes from DMSO as a solvate **3**·**DMSO** in the non-centrosymmetric space group *P2_1_* of the monoclinic system with two molecules per unit cell. The asymmetric unit contains one amido-acid CytSQOH molecule and one lattice DMSO molecule, which, as shown by refinement, exhibits a slight disorder of the S atom in two positions (C and O atoms are common to both DMSO orientations) with an occupancy of 0.949(3) and 0.051(3) Å, so for clarity, Figure 1 shows only the main orientation. The CYT framework adopts its typical rigid tricyclic quinolizidine conformation, comprising a planar pyridone ring (ring A, atoms 7–12), an envelope-shaped ring B (atoms 5–7, 12, 1, 13) with bridgehead atom C13 displaced by 0.718(3) Å from the mean plane, and a chair-shaped piperidine ring (ring C, atoms 1–5, 13). The pyridone ring is tilted relative to the squaramide moiety. Amido-acid SQOH (A, B and C rings see Scheme 1) is nearly planar and forms a dihedral angle of approximately 32.1(2)° with the pyridine ring.

In the gas phase, the fully DFT-optimized structure retains the overall cytisine conformation. However, significant differences are observed in the relative orientation of the squaramide fragment. The dihedral angle between the squaric unit and the pyridine ring increases to 60.79°, indicating conformational flexibility and a strong influence of crystal packing (Figure 1d). A complete set of the optimized structural parameters of **3** is provided in Table S1 in the Supplementary Materials.

**Figure 2:**
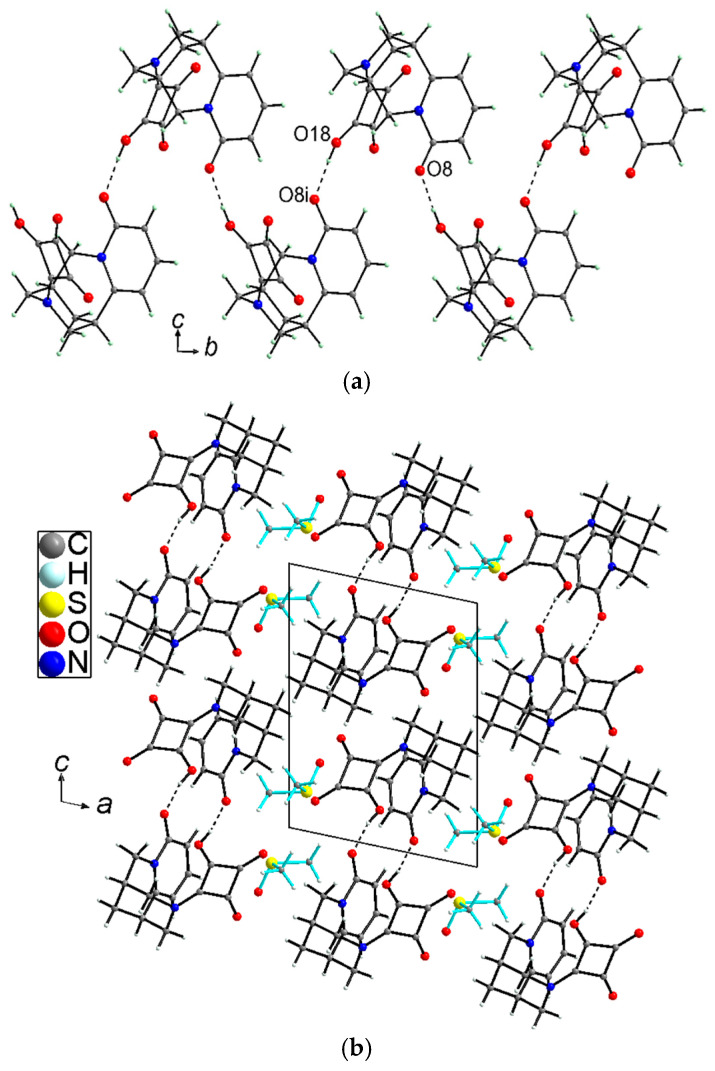
(**a**) View of a right-handed helix with an O–H···O hydrogen bond running along the *b*-axis and (**b**) the packing diagram of the helices with the lattice DMSO molecules. The DMSO lattice solvent molecules are marked in light-blue.

In the solid state, the dominant structural feature is the formation of hydrogen-bonded helices. CytSQOH molecules in crystal **3·DMSO** are interconnected via strong, nearly linear O–H···O hydrogen bonds (where O···O = 2.460(2) Å), formed between the carboxylic OH group and the carbonyl oxygen of the pyridone ring of a neighboring molecule. These interactions give rise to right-handed helices extending along the *b*-axis (Figure 2a). The hydrogen-bonding pattern is consistent with electrostatic complementarity, as the interactions occur between regions of opposite electrostatic potential (Figure 1c).

The crystal packing of **3·DMSO** consists of parallel helices arranged along the b-axis, with the voids between them occupied by DMSO molecules (Figure 2b). No directional hydrogen bonds are formed between the helices and the solvent molecules; instead, stabilization arises from van der Waals interactions and weak C–H···O electrostatic forces between C and O in the range 3.07–3.35 Å (Table S2 in Supplementary Materials).

#### 2.2.2. Squaramide **3**

**Figure 3:**
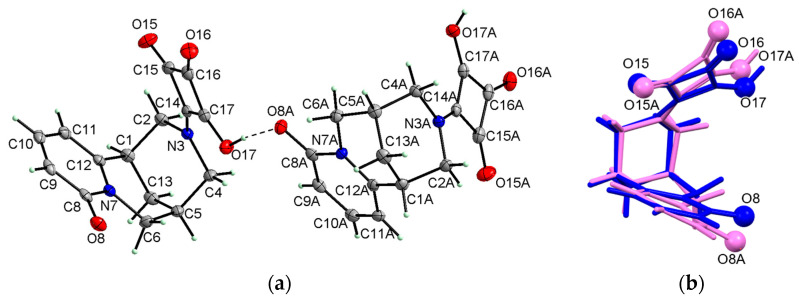
(**a**) Asymmetric unit of **3** with atom labeling scheme and displacement ellipsoids are shown at the 50% probability level, H atoms are depicted with arbitrary radii and (**b**) overlay of the independent squaramidic acid CytSQOH molecules: blue—molecule **1** with O8, O15, O16 i O17 and pink—molecule 1A with O8A, O15A, O16A i O17A.

Squaramidic acid CytSQOH (**3**) was obtained by repeated recrystallization of a poorly soluble precipitate from the mixture of methanol and isopropanol. Compound **3** crystallizes in a way similar to that of **3**·**DMSO** in the non-centrosymmetric space group P2_1_ of the monoclinic system, with four molecules per unit cell but without lattice solvent. The asymmetric unit contains two independent CytSQOH molecules (**3**), as shown in Figure 3a, which are linked via nearly linear O–H···O hydrogen bonds (with an O···O distance of 2.510 (2) Å) to form a dimeric motif.

The conformations of the two independent molecules of **3** and **3**·**DMSO** are closely related, as illustrated in Figure 3b. In both cases, the CYT framework retains its characteristic rigid tricyclic quinolizidine structure, with a planar pyridone ring (ring A), an envelope-shaped ring B with the bridgehead atom C13 (or C13A) displaced by 0.720(3) and 0.741(3) Å, respectively, and a chair-shaped piperidine ring (ring C). However, the main conformational difference between the two molecules concerns the orientation of the squaric acid fragment relative to the planar pyridone ring. The corresponding dihedral angle is 48.3(3)° in one molecule (with O8) and increases to 67.5(3)° in the second (with O8A).

**Figure 4:**
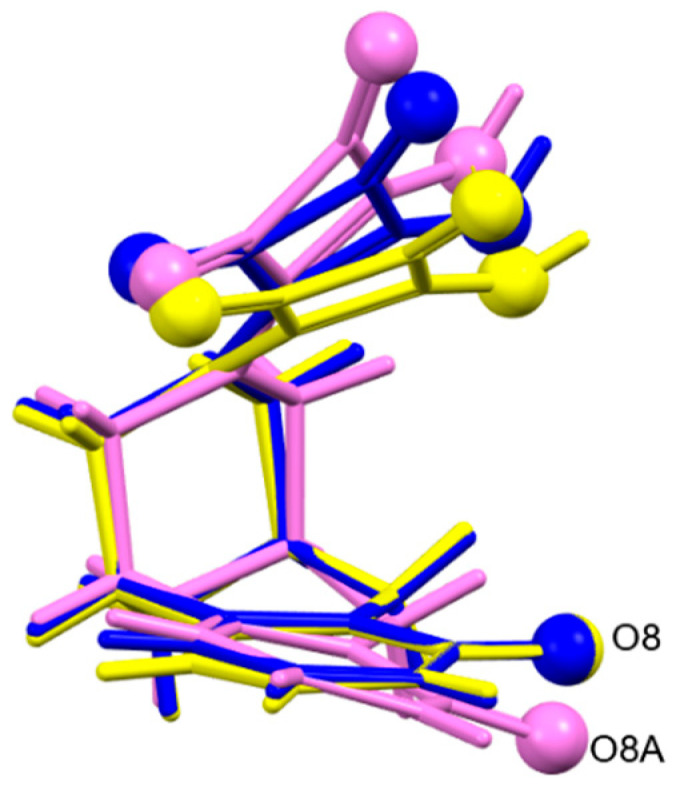
Overlay of the squaramidic acid CytSQOH molecules showing the differences in the conformations in crystals **3** and in **3**·**DMSO**. Color code: yellow— conformation in crystal **3**·**DMSO** and the other colors as in Figure 3b (i.e., blue—molecule **1** and pink molecule 1A in crystal **3**).

A comparison of structure **3** with the solvated structure **3**·**DMSO** (Figure 4) shows that the squaramide fragment adopts a significantly less inclined orientation (~32°) in the solvate than in crystal **3**, whose molecules are independent and interconnected by almost linear and relatively strong hydrogen bond O–H···O with O···O distance of 2.510 (2) Å into dimeric structure that is replicated translationally along the *b* axis to form a wave-like hydrogen-bonded O–H···O chain.

**Figure 5:**
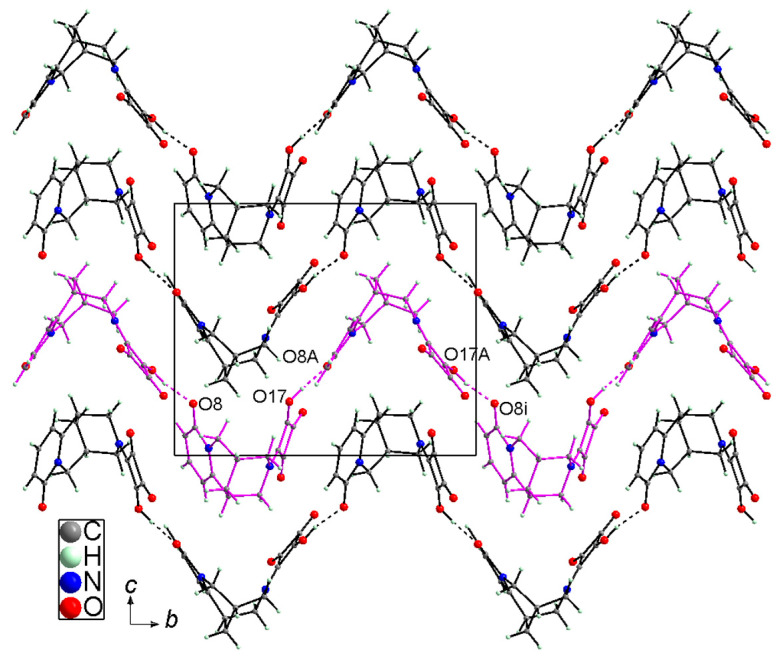
Wave-like arrangement of amide acid CytSQOH molecules into chains running along the *b*-axis in crystal **3**. One of the chains is marked in pink.

In the crystal, the dimeric units align along the *b*-axis via O–H···O hydrogen bonds, forming a wave-like one-dimensional chain, as shown in Figure 5. Additional O–H···O interactions (O···O = 2.531(2) Å) link adjacent dimers within the chain (Table S2, Supplementary Materials). No significant directional interactions are observed between neighboring chains; instead, crystal packing is governed by van der Waals forces and weak C–H···O contacts (3.052–3.393 Å, Table S3, Supplementary Materials).

#### 2.2.3. Squaramide **4**

**Figure 6:**
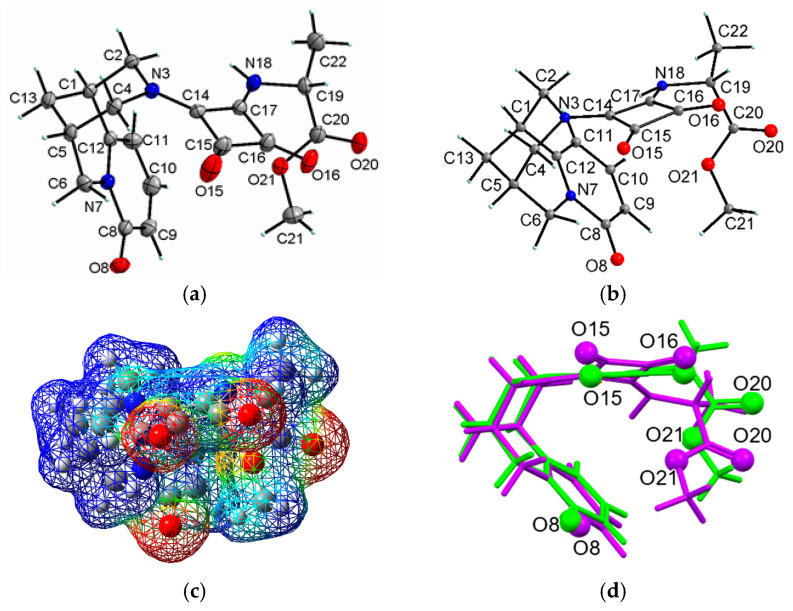
(**a**) Asymmetric unit of **4** with atom labeling scheme. Displacement ellipsoids are shown at the 50% probability level, H atoms with arbitrary radii. (**b**) DFT optimization of alanine-derived cytisine–squaramide and (**c**) 3D-molecular electrostatic potential mapped onto the total electron density isosurface (0.008 eÅ^−3^), red and blue in the EP represent values between −0.05 and 0.05 eÅ^−1^. (**d**) A comparison of the conformation of alanine-derived cytisine–squaramide molecules: DFT—purple, X-ray—green.

Alanine-derived cytisine–squaramide (**4**) crystallizes from acetonitrile in the non-centrosymmetric space group P2_1_2_1_2_1_ of the orthorhombic system, with four molecules per unit cell. The asymmetric unit contains one molecule, as shown in Figure 6a. The molecular structure features the typical CYT scaffold, comprising a planar pyridone ring, an envelope-shaped ring B (C13 displaced by 0.604 (3) Å), and a chair-shaped piperidine ring (ring C). The squaramide moiety is nearly planar and forms a dihedral angle of 46.5(3)° with the pyridone ring. The alanine methyl ester fragment (N18, C19, C20, O20, O21 and C21) is also nearly planar and is inclined by 72.8 (3)° to the squaramide ring (Figure 6b). The DFT-optimized structure (Figure 6b) and its comparison with the experimental geometry (Figure 6a) reveal that the overall molecular framework is preserved, although notable conformational differences are observed (Figure 6d). In particular, the displacement of the C13 atom increases (0.741 Å), and the dihedral angle between the squaramide and the pyridone ring increases to 58.0°. In contrast, the orientation of the methyl ester fragment (N18, C19, C20, O20, O21, C21) remains essentially unchanged (72.3°), (Figure 6d). A complete set of optimized parameters is provided in Table S4 (Supplementary Materials).

**Figure 7:**
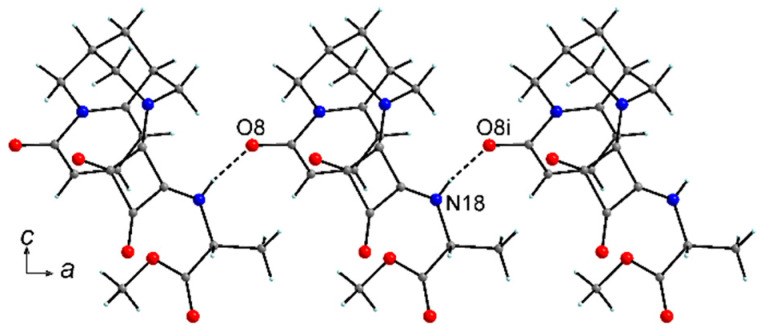
View of the hydrogen-bonded N–H···O chain of molecules in the crystal of compound **4**. Symmetry code: (i) x + 1, y, z.

In the crystal of compound **4**, the molecules are connected via N–H···O hydrogen bonds (N···O = 2.961(3) Å), forming one-dimensional chains along the *a*-axis, as shown in Figure 7. These interactions occur between regions of opposite electrostatic potential (Figure 6c).

**Figure 8:**
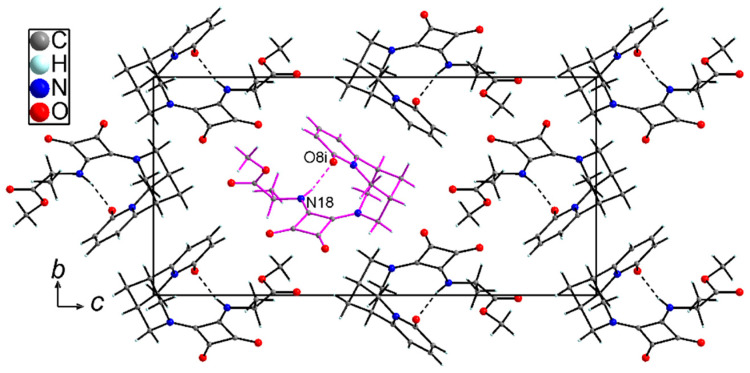
Arrangement of chains of alanine-derived cytisine–squaramide molecules, running along the *a*-axis in crystal **4**. One of the chains is marked in pink.

In the crystal of compound **4**, the chains are arranged parallel to the *a*-axis and interact mainly through van der Waals forces and weak C–H···O contacts (Figure 8). No significant interchain hydrogen bonding is observed (Table S5, Supplementary Materials).

#### 2.2.4. Squaramide **5**

**Figure 9:**
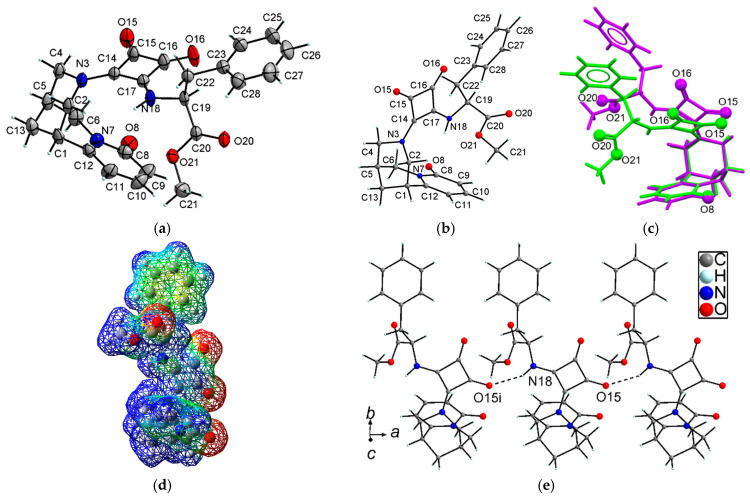
(**a**) Asymmetric unit of **5** with atom labeling scheme and displacement ellipsoids are shown at the 50% probability level, H atoms with arbitrary radii, (**b**) DFT optimized molecule, (**c**) overlay of molecules showing the differences between DFT (purple) and X-ray (green), (**d**) view of the 3D-molecular electrostatic potential mapped onto the total electron density isosurface (0.008 eÅ^−3^), red and blue in the EP represent values between −0.05 and 0.05 eÅ^−1^ and (**e**) the hydrogen-bonded N–H···O chain of molecules in the crystal **5** (symmetry code: (i) x + 1, y, z.).

Phenylalanine-derived cytisine–squaramide **5** crystallizes from acetonitrile in the non-centrosymmetric space group P2_1_2_1_2_1_ of the orthorhombic system, with four molecules per unit cell. The asymmetric unit consists of one molecule (Figure 9a). The (–)-cytisine framework adopts the characteristic rigid tricyclic conformation, comprising a planar pyridone ring, an envelope-shaped ring B (C13 displaced by 0.732(3) Å), and a chair-shaped piperidine ring. The squaramide moiety is nearly planar and forms a dihedral angle of 38.0(3)° with the pyridone ring, while the phenyl ring of the phenylalanine substituent is inclined by 50.2(3)° to ring A. The DFT-optimized structure of squaramide **5** is shown in Figure 9b, and its comparison with the experimental geometry is presented in Figure 9c. While the bond lengths and angles remain largely unchanged, noticeable conformational differences are observed. In particular, the dihedral angle between the squaramide and pyridone rings increases to 68.9° in the gas phase, compared to 38.0(3)° in the crystal. Similarly, the phenyl ring of phenylalanine is inclined at a greater angle (67.0°) to the pyridone plane in the optimized structure. These differences highlight the influence of intermolecular interactions on the molecular conformation in the solid state. A complete set of optimized geometrical parameters of squaramide **5** is provided in Table S6.

The calculated 3D molecular electrostatic potential (MESP), mapped onto the total electron density isosurface (0.008 eÅ^−3^), is shown in Figure 9d. The MESP surface reflects the distribution of electron density and identifies regions of complementary electrostatic potential (EP) that are relevant for intermolecular recognition.

In the crystal, translationally related molecules interact along the *a*-axis *via* relatively weak N–H···O hydrogen bonds (N···O = 2.946(3) Å; Table S7), forming one-dimensional chains (Figure 9e). These interactions occur between regions of opposite electrostatic potential (EP), consistent with the MESP analysis (Figure 9d).

**Figure 10:**
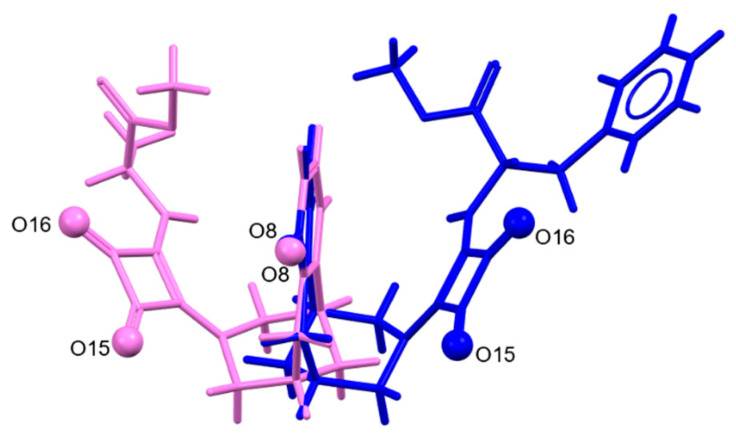
Overlay of molecules **4** (pink) and **5** (blue) to compare their conformations. The C atoms of the four-membered ring of squaric acid are common to both molecules **4** and **5**.

A comparison of the molecular conformations of compounds **4** and **5** indicates that the pyridone rings are well aligned, whereas differences are observed in the orientation of ring B and the squaramide fragment (Figure 10). In particular, the envelope conformation of ring B is preserved, but the direction of puckering is the opposite in the two structures. The inclination of the squaramide ring relative to the pyridone ring also differs slightly, amounting to 46.5(3)° for compound **4** and 50.2(3)° for compound **5**.

**Figure 11:**
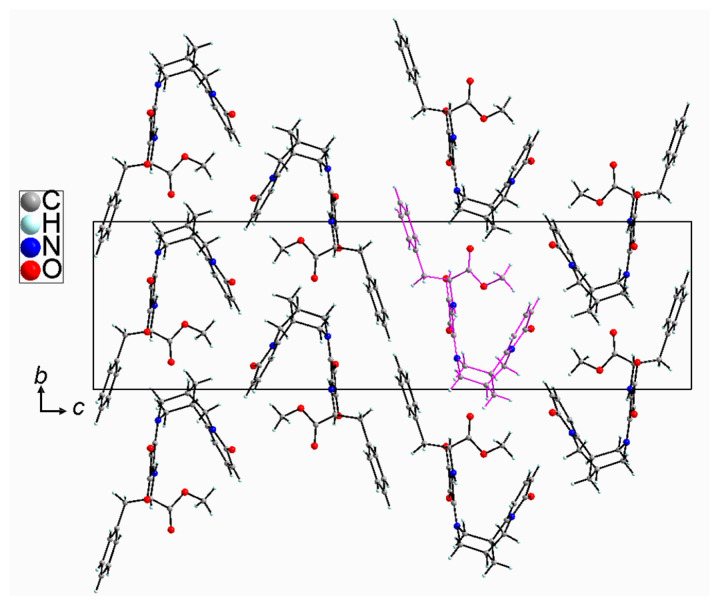
Arrangement of chains of phenylalanine-derived cytisine–squaramide molecules along the *a*-axis in the crystal of compound **5**. One of the chains is marked in pink.

The crystal packing of **5** shows that the hydrogen-bonded chains extend along the *a*-axis and are arranged in a parallel manner (Figure 11). Interactions between adjacent chains are dominated by van der Waals forces and weak C–H···O contacts. Due to the presence of the bulkier phenylalanine substituent, the packing is more spatially expanded compared to that of compound **4**, and no classical interchain hydrogen bonds are observed (Table S7, Supplementary Materials).

#### 2.2.5. Structure of Co-Crystal **6** (Co-Crystal of Amide Ester **1** with Amido Acid **3**)

**Figure 12:**
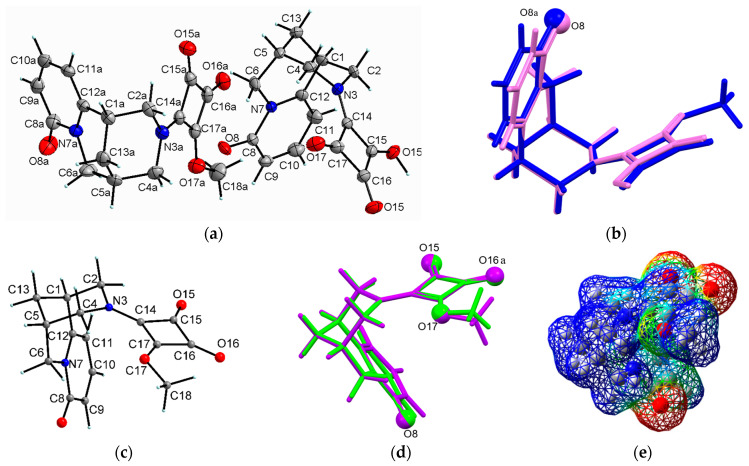
(**a**) Asymmetric unit of compound **6** with atom labeling scheme, displacement ellipsoids are shown at the 30% probability level, H atoms with arbitrary radii, (**b**) overlay of amide methyl ester CytSQ-OMe (**1**, blue) and amido-acid CytSQOH (**3**, pink) molecules in crystal **6**, (**c**) DFT optimized conformation of molecule **1**, (**d**) overlay of molecules **1** showing the differences between DFT (purple) and X-ray (green) and (**e**) 3D-molecular electrostatic potential for molecule **1**.

Recrystallization of the amide ester CytSQ-OMe (**1**) with the amido-acid CytSQOH (**3**) from DMSO afforded high-quality single crystals of co-crystal **6** suitable for X-ray diffraction analysis. The co-crystal crystallizes in the non-centrosymmetric space group *P*2_1_ of the monoclinic system. The asymmetric unit consists of one molecule of the amide ester CytSQ-OMe, **1**, and one molecule of the amide acid CytSQOH, **3**, (Figure 12a). The overall conformations of the (–)-cytisine frameworks in both components are very similar and retain the characteristic rigid tricyclic quinolizidine motif. The main conformational differences are associated with the orientation of the squaramide fragment with respect to the planar pyridone ring, with a dihedral angle in the ester (63.3(3)°) larger than in the acid (55.5(3)°) (Figure 12b).

DFT calculations were performed for both molecular components; however, as the optimized structure of the amide acid CytSQOH has been discussed above, only the ester component is considered here. The DFT-optimized structure of CytSQ-OMe (Figure 12c) and its comparison with the experimental geometry (Figure 12d) show good agreement, with only minor differences observed in the orientation of the methyl ester group. They are reflected in the torsion angle C16–C17–O17–C18, which amounts to −10.33° in the optimized structure and 4.7(5)° in the crystal. A complete set of optimized geometrical parameters is provided in Table S8 (Supplementary Materials).

The molecular electrostatic potential (MESP), mapped onto the total electron density isosurface (0.008 eÅ^−3^), is shown (Figure 12e) and illustrates regions of complementary electrostatic potential relevant to intermolecular interactions.

**Figure 13:**
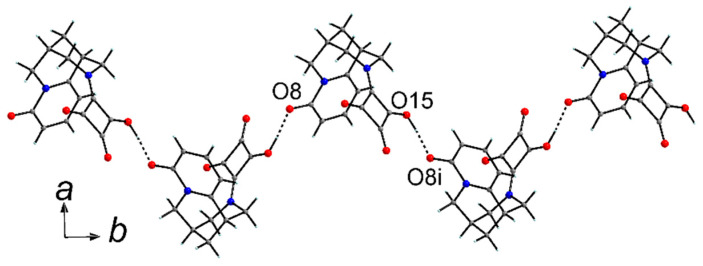
View of the hydrogen-bonded O–H···O chain of amido-acid CytSQOH (**3**) molecules in the co-crystal **6**. Symmetry code: (i) −x, y + 1/2, −z + 1.

In the crystal structure of compound **6**, only the amide acid CytSQOH molecules act as hydrogen-bond donors, whereas the ester component lacks a suitable donor functionality. As a result, hydrogen bonding occurs exclusively between the acid molecules, leading to the formation of one-dimensional O–H···O chains extending along the *b*-axis (Figure 13). These chains are generated by translation combined with the 2_1_ screw axis and are stabilized by nearly linear hydrogen bonds (O···O = 2.510(2) Å; Table S9), consistent with the motif observed in compound **3** and **3·DMSO**.

**Figure 14:**
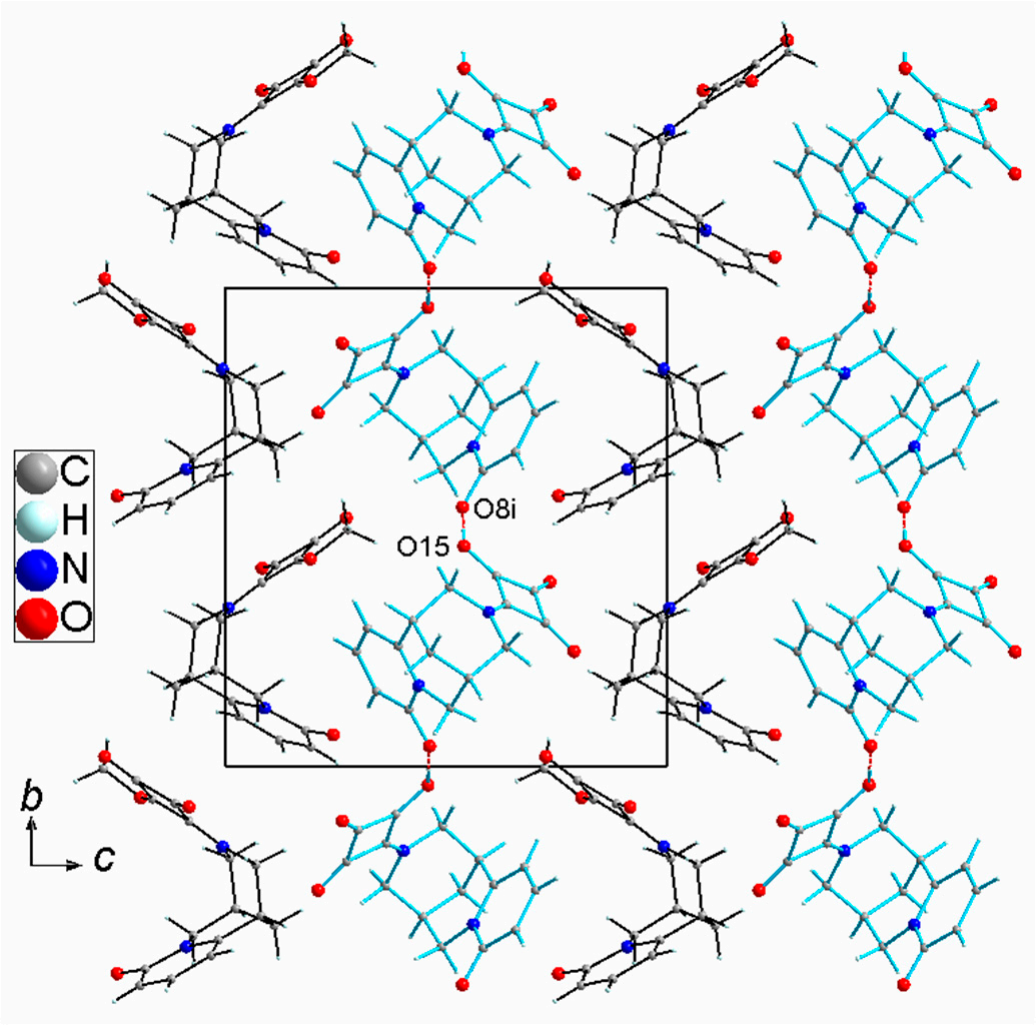
View of the packing of CytSQ-OMe (**1**) with CytSQOH (**3**) molecules in the co-crystal **6**, showing alternating arrangement of amide ester molecules and the chains of amido-acid molecules (blue).

The overall crystal packing in co-crystal **6** shows that the hydrogen-bonded chains of CytSQOH are arranged in parallel to the *b*-axis and alternate with stacks of CytSQ-OMe molecules, which are also aligned along this direction (Figure 14). No classical hydrogen bonds are formed between the two components; instead, the packing is governed by van der Waals interactions and weak C–H···O contacts (Table S9, Supplementary Materials).

#### 2.2.6. Structure of Co-Crystal **7** (Co-Crystal of Amido Ethyl Ester **2** with Amido Acid **3**)

**Figure 15:**
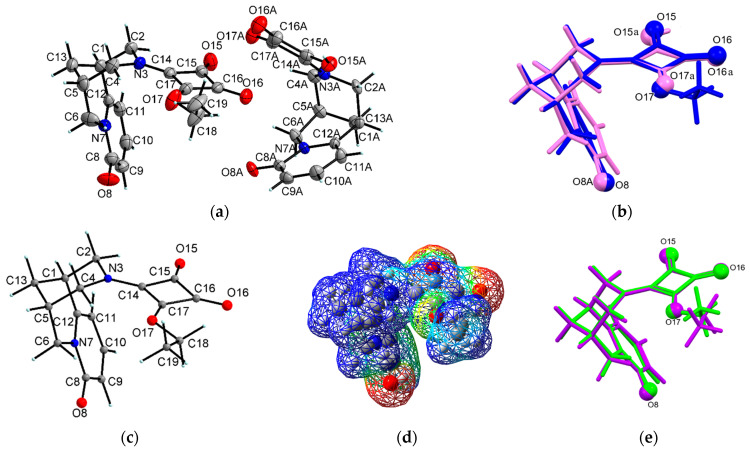
(**a**) Asymmetric unit of co-crystal **7** with atom labeling scheme and displacement ellipsoids are shown at the 30% probability level, H atoms with arbitrary radii, (**b**) overlay of amido ethyl ester CytSQ-OEt (**2**, blue) and amide acid CytSQOH (**3**, pink) molecules in crystal **7**, (**c**) DFT optimized conformation of molecule **2**, (**d**) 3D molecular electrostatic potential and (**e**) comparison of the conformations of CytSQ-OEt (**2**), purple—DFT conformation, green—X-ray conformation in co-crystal **7**.

Recrystallization of the amide ethyl ester CytSQ-OEt (**2**) with the amide acid CytSQOH (**3**) from DMSO in a 1:1 molar ratio afforded high-quality single crystals of co-crystal **7** suitable for X-ray diffraction analysis. The co-crystal crystallizes in the non-centrosymmetric space group *P*2_1_ of the monoclinic system. The asymmetric unit consists of one molecule of the amide ethyl ester CytSQ-OEt (**2**) and one molecule of the amide acid CytSQOH (Figure 15a).

The CYT frameworks in both components retain the characteristic rigid tricyclic quinolizidine conformation and are very similar. The main conformational differences arise from the relative orientation of the squaramide fragment with respect to the planar pyridone ring, with a dihedral angle in the ester (66.5(3)°) larger than in the acid (58.5(3)°) (Figure 15b).

DFT calculations were performed for the amide ethyl ester component, whereas the optimized structures of the amide acid CytSQOH (**3** and **3·DMSO**) have been discussed above. The DFT-optimized structure of CytSQ-OEt (**2**, Figure 15c), together with the corresponding molecular electrostatic potential (Figure 15d), shows good agreement with the experimental geometry (Figure 15e), with only a minor difference in the orientation of the ethyl group and a slight variation in the squaramide–pyridone angle C17–O17–C18–C19. In the DFT-optimized molecule, this angle is 163.34, while in the co-crystal **7**—it is 123.8 (7)°. In addition, a slight difference is also found in the inclination of the planar squaric acid ring to the planar pyridone ring: in DFT = 62.30° and in co-crystal **7** = 66.5(3)°. Fully optimized geometrical parameters are provided in Table S10 (Supplementary Materials).

**Figure 16:**
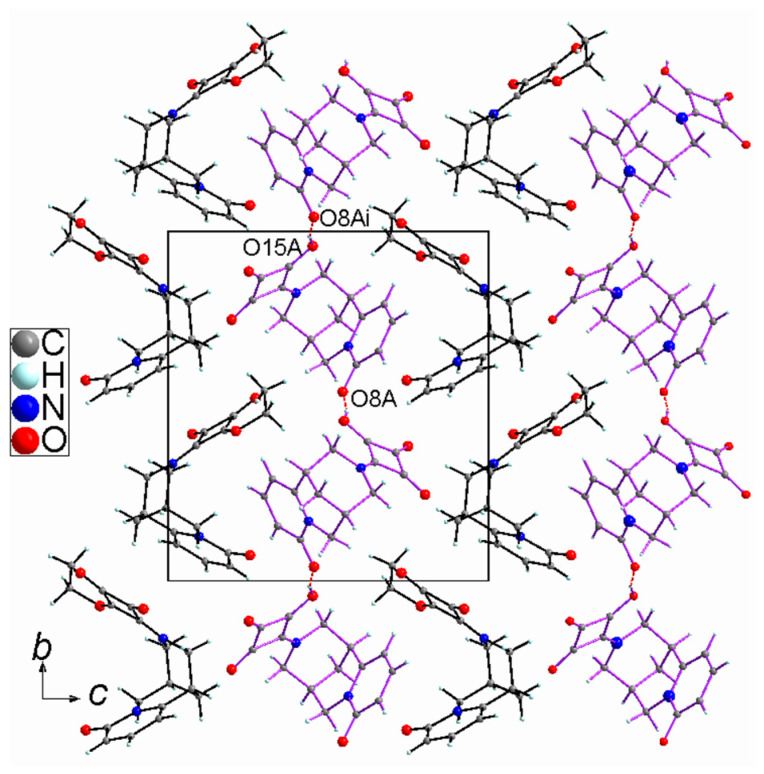
View of the packing of amido ethyl ester CytSQ-OMe (**1**) with the amido-acid CytSQOH (**3**) molecules in the co-crystal **7**, showing alternating arrangement of amide ester molecules and the chains of amido-acid molecules (marked in purple).

In the co-crystal **7** structure, the amide acid CytSQOH molecules act as hydrogen bond donors and form one-dimensional helical chains extending along the *b*-axis (Figure 16). These chains are generated by the 2_1_ screw axis and are stabilized by nearly linear O–H···O hydrogen bonds (O···O = 2.512(4) Å; Table S11), in accordance with the motifs observed in compounds **6**, **3** and **3·DMSO**.

The amide ethyl ester CytSQ-OEt (**2**) molecules are arranged in parallel stacks, also extending along the *b*-axis. No classical hydrogen bonds are formed between the ester stacks and the hydrogen-bonded chains of the acid component; the crystal packing is governed by van der Waals interactions and weak electrostatic contacts, including C–H···O interactions (Table S11, Supplementary Materials).

Across compounds **3**–**7**, the (–)-cytisine scaffold consistently preserves its rigid tricyclic quinolizidine framework, with only minor variations in ring puckering parameters, confirming its conformational robustness. In contrast, the squaramide fragment exhibits pronounced conformational flexibility, as reflected in the wide range of dihedral angles to the pyridone ring. These variations are strongly influenced by the molecular environment, including the presence or absence of solvent, the nature of the substituent (aliphatic vs. aromatic), and co-crystal formation. Notably, a comparison with DFT-optimized geometries indicates a systematic tendency toward larger dihedral angles in the gas phase, highlighting the key role of intermolecular interactions in stabilizing more compact conformations in the solid state.

The supramolecular organization is governed primarily by directional hydrogen bonding involving the amide acid functionality, which acts as a reliable synthon across all structures. In pure compound **3** and in co-crystals **6** and **7**, the O–H···O interactions dominate, leading to the formation of one-dimensional chains or helical motifs, whereas in derivatives **4** and **5**, the N–H···O hydrogen bonds define the assembly. Despite differences in substituents and crystal systems, the resulting architectures are consistently one-dimensional, with higher-order packing driven mainly by van der Waals interactions and weak C–H···O contacts. The absence of strong interchain hydrogen bonding highlights the modular nature of these systems and suggests that subtle modifications in substituent structure can effectively tune both molecular conformation and crystal packing.

### 2.3. Spectroscopic Analysis

**Figure 17:**
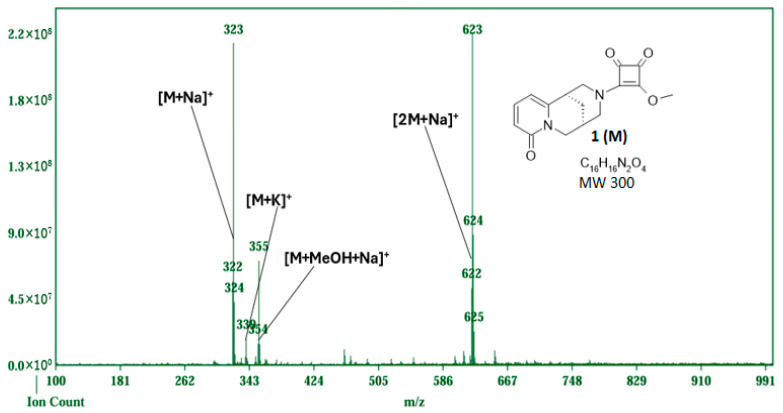
ESI-MS spectrum of squaramate **1** (MW 300) in methanol.

**Figure 18:**
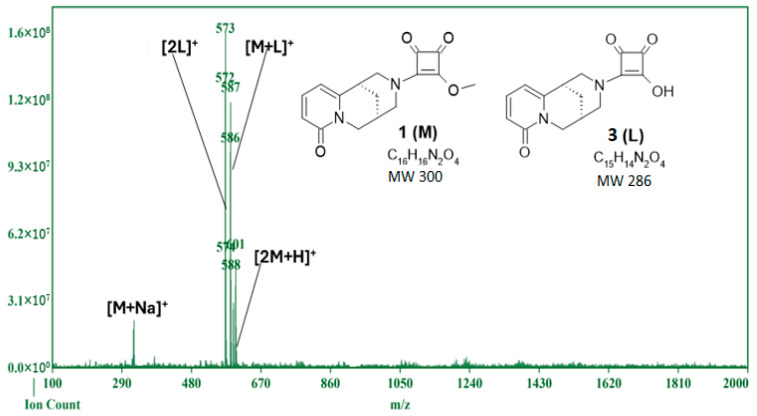
ESI-MS spectrum of co-crystal **6** (MW 586) in methanol.

**Figure 19:**
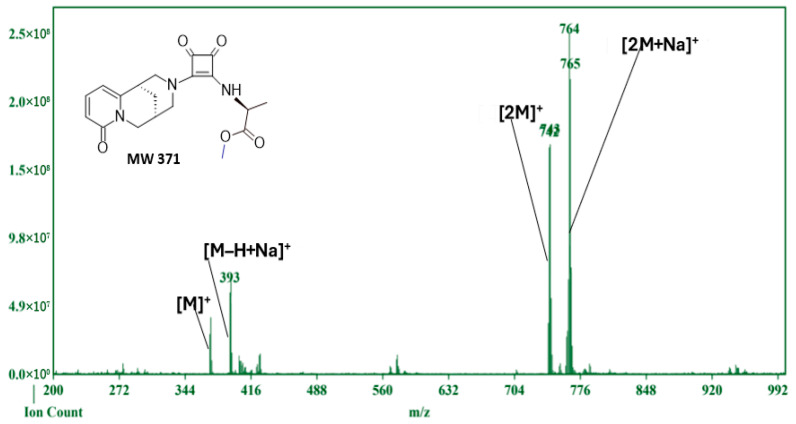
ESI-MS spectrum of **4** (MW 371) in methanol.

**Figure 20:**
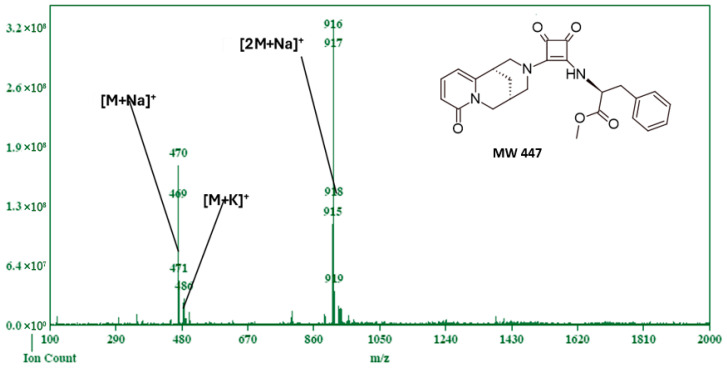
ESI-MS spectrum of **5** (MW 371).

ESI-MS analysis of methanolic solutions containing the respective components (Figure 17 and Figure 18) revealed characteristic signals corresponding to squaramate **1** (Figure 17) and to the mixture of squaramate **1** and CytSQOH (**3**) (Figure 18), along with an additional prominent peak attributed to a noncovalent hydrogen-bonded complex—a feature typical of squaric acid derivatives. ESI-MS spectra of both target compounds **4** (MW 371, Figure 19) and **5** (MW 433, Figure 20) were collected following the flash column separation step, which ensured thorough purification.

The synthesis leading to the alanine methyl ester derivative **4** was confirmed by ESI–MS analysis (Figure 19), which revealed a signal at *m*/*z* 371 corresponding to the molecular ion [M]^+^. Notably, the relative intensity of this peak was significantly lower than that of the dimeric species observed at *m*/*z* 742, assigned to [2M]^+^ (Figure 19). The pronounced formation of such dimeric ions is consistent with the well-documented tendency of alkaloid-derived, hydrogen-bonding systems to undergo noncovalent self-association under ESI conditions, particularly at elevated analyte concentrations. Additionally, sodium adducts were clearly observed, with signals at *m*/*z* 393 and 764 corresponding to [M + Na]^+^ and [2M + Na]^+^, respectively (Figure 19). The presence of both monomeric and dimeric sodiated species further supports the propensity of these systems to coordinate metal ions. A similar behavior was observed for conjugate **5**. In its ESI–MS spectrum (Figure 20), no intense signals corresponding to [M + H]^+^ ions were detected. Instead, prominent peaks at *m*/*z* 470 and 917 were observed, attributable to the sodium adducts [M + Na]^+^ and [2M + Na]^+^, respectively (Figure 20).

**Figure 21:**
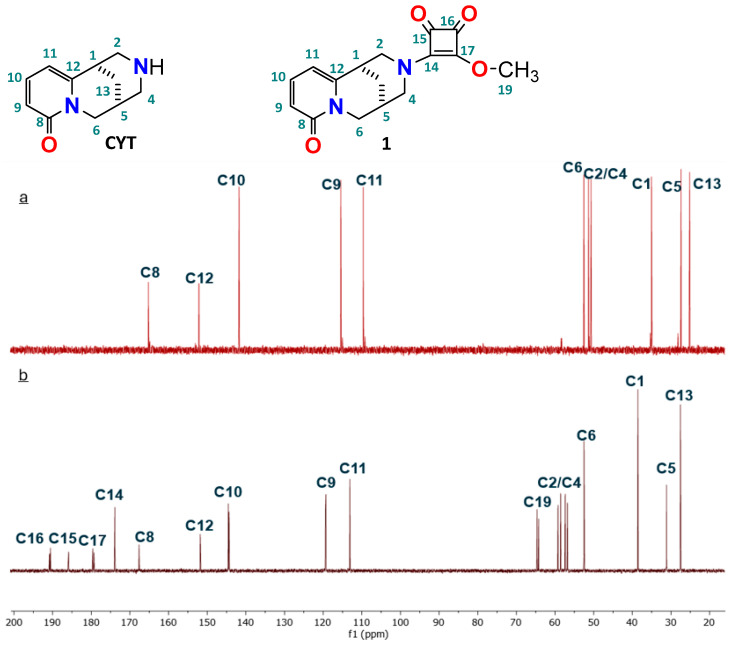
The comparison of ^13^C NMR spectra in D_2_O of **CYT** (**a**) and squaramate **1** (**b**).

To further support the structural assignment, both ^1^H and ^13^C NMR spectra of the obtained compounds were recorded. Analysis of the NMR data provided insight into conformational dynamics of the investigated amides, which were further elucidated by ^13^C NMR spectroscopy of compound **1** recorded in D_2_O (Figure 21b). The observed doubling of carbon chemical shifts indicates a slow rotation about the amide bond on the NMR timescale, resulting in the presence of distinct cis/trans amide bond rotamers. This behavior arises from the partial double-bond character of the C–N amide linkage, which imposes a relatively high rotational barrier and thus allows both rotameric forms to be observed as separate sets of signals. This interpretation is consistent with previously reported data for structurally related alkaloid-derived amides, in which restricted rotation around the amide bond leads to the resolution of distinct resonances for individual rotamers.

The carbon chemical shifts were displaced downfield, reflecting substantial deshielding effects arising from the strong electron-withdrawing squarate moiety (Figure 21). Among the solvents tested, D_2_O provided superior spectral resolution compared to those of DMSO-d_6_ and CDCl_3,_ likely due to improved solvation and reduced intermolecular aggregation. However, the use of D_2_O also results in a rapid H/D exchange of labile protons, leading to the disappearance of exchangeable –OH signals. Consequently, the characteristic ester and/or carboxylic acid proton resonances of compound **3** were not observed in the ^1^H NMR spectra. In contrast to the ^13^C NMR spectrum of compound **1** (Figure 21), the spectra of compounds **4** and **5** do not exhibit a doubled set of signals (Supplementary Data, Figures S9–S10 and Figures S11–S12, respectively). This is most likely a result of steric hindrance caused by the bulkier substituent at C-17, which may bias the conformational equilibrium toward a single predominant rotamer and/or render rotamer-specific carbon chemical-shift differences too small to resolve. However, the ^1^H NMR spectra still indicate restricted amide rotation, most clearly for compound **5**. A similar signal overlap has also been reported for other squaramides [29,30]. Additionally, analysis of the recorded ^13^C NMR spectra of compounds **4** and **5** revealed that two signals from the cytisine moiety, corresponding to the chemical shifts in C2 and C4, are barely visible in the spectra recorded in DMSO-d_6_ (Supplementary Data, Figure S9 and Figure S11, respectively).

Intensities of the peaks corresponding to phenylalanine ester derivative are relatively lower compared to those of the alanine analog, which is consistent with increased line broadening. The chemical shifts in C24 and C28, expected to be very similar, appear as distinct peaks (δ 129.1 and 129.8 ppm, respectively), indicating that these carbons are not magnetically equivalent, possibly due to the combined influence of neighboring carbonyl groups and conformational asymmetry. The methoxy carbon C21, common to both products, resonates at δ 52.3 ppm, while formation of the benzylic bridge shifts the peak assigned to C22 downfield to δ 37.8 ppm.

**Figure 22:**
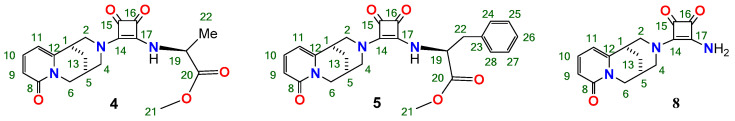
Structures of the products **4, 5** and the known derivative **8** [30] with numbered carbon atoms.

For signal assignment, compound **6** was used as a structural model. Its previously published ^1^H and ^13^C NMR chemical-shift data [30] were used as a reference set for the interpretation of the spectra of compounds **4** and **5**. Particular attention was paid to diagnostic resonances of the cytisine fragment and the aliphatic region. In the reported spectrum of compound **8** (Figure 22), the most shielded aliphatic carbons (C1, C5, and C13) resonate in the δ ≈ 20–40 ppm region. Observation of signals in the same range for compounds **4** and **5** supported their assignment. Comparative analysis of characteristic resonances, including those assigned to the squarate carbonyl carbons, benzylic bridge carbon and methoxy carbon, further confirmed structural correspondence between the model compound and the newly synthesized derivatives. Minor variations in chemical shifts were attributed to substituent effects and local conformational differences. Although the assignments of C1 and C5 in our spectra appear interchanged relative to ref. [30], the overall chemical-shift distribution and pattern of diagnostic signals remain in full agreement with the literature data, thereby validating the structural assignments of compounds **4** and **5**.

**Figure 23:**
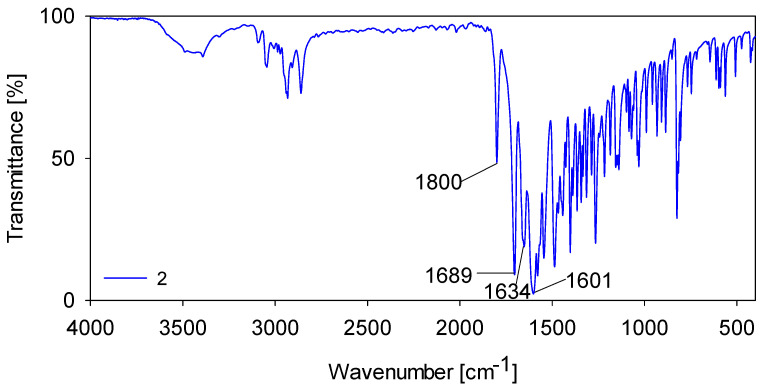
The FT-IR spectrum of compound **1** (amorphous solid) was recorded using the KBr pellet technique (2 mg sample/200 mg KBr).

**Figure 24:**
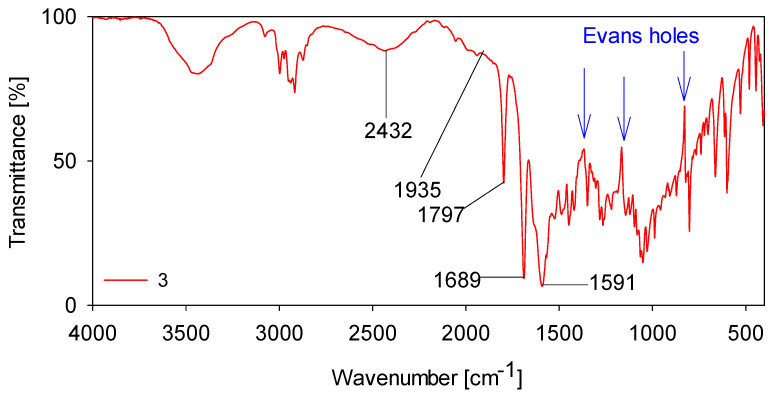
The FT-IR spectrum of compound **3** (crystalline form) was recorded using the KBr pellet technique (2 mg sample/200 mg KBr).

Figure 23 and Figure 24 compare the FT-IR spectra of compound **1** (amorphous solid) and **3** (crystalline form), recorded using the KBr pellet technique (2 mg sample/200 mg KBr). This comparison shows the spectroscopic signature of the very strong hydrogen bonds that stabilize the dimeric structure of **3** in the solid state.

According to the structural parameters of the two hydrogen bonds of the [O⋯HO] type present in the crystalline structure of **3**, they can be classified as short and strong hydrogen bonds. In the crystal structure of **3**, the asymmetric unit contains two independent CytSQOH dimeric motifs linked via nearly linear O–H···O hydrogen bonds (with an O···O distance of 2.510(2) Å).

In the FT-IR spectrum of **3**, a very intense absorption band extending over the 1400–700 cm^−1^ region, the so-called D band [32], clearly indicates the presence of a very strong hydrogen bond in the crystal structure of the complex. According to the structural data summarized in Table S3, this band is associated with the intermolecular hydrogen bonds of the type O17–H···O8A and O17A–H17A···O8^i^. A characteristic feature of the D band is the presence of Evans holes [33], which are clearly visible at approximately 1370 cm^−1^, 1164 cm^−1^, and 830 cm^−1^ and are indicated by arrows in the spectrum (Figure 24).

An Evans hole (or Evans transmission window) appears when a sharp, weak absorption band (ν_2_) falls within a broad and intense absorption envelope (ν_1_) associated with proton stretching in a strong hydrogen bond, resulting in the formation of a transmission window due to anharmonic coupling (Fermi resonance). Such spectral features are characteristic of systems containing very short and strong hydrogen bonds, typically with O···O distances of approximately 2.4–2.5 Å [33].

The FT-IR spectrum of crystalline **3** exhibits two moderately intense broad bands with maxima at approximately 2432 and 1935 cm^−1^, together with a broad absorption near 1500 cm^−1^ and one very intense band extending over the 1400–700 cm^−1^ region. The spectral behavior is characteristic of relatively short (O···HO) hydrogen bonds described by an asymmetric double-minimum proton potential, as proposed by Hadži [32,34].

The broad absorption observed in the spectrum of the crystal is consistent with theoretical predictions for asymmetric O···HO hydrogen bonds with lengths in the range 2.50–2.60 Å [35,36], and a separate band at approximately 2435 cm^−1^ should be assigned to the 0–2 vibrational transition. Furthermore, very similar spectral behavior of the bands at approximately 2432 cm^−1^, 1935 cm^−1^, and circa 1500 cm^−1^ has been reported in the spectra of various compounds containing asymmetric (O···HO) hydrogen bonds with comparable structural parameters [37].

Stretching vibrations of the squaric dicarbonyl fragment (C15 and C16) appear for both derivatives in the 1740–1800 cm^−1^ range. The bands at ν = 1679 cm^−1^ and 1646 cm^−1^ are assigned to ν(C=O) conjugated with a double bond in the ring, as well as the stretching vibrations of the ester carbonyl (C20) and the pyridone moiety (C8), respectively, are present in the spectra of all studied compounds. Additionally, the FT-IR spectra of squaramide derivatives **4** and **5** exhibit broad and intense absorption bands at ν = 3246 cm^−1^ and 3206 cm^−1^, respectively (Figures S13 and S14, Supplementary Materials), corresponding to ν(N–H) stretching vibrations and confirming amide bond formation. The appearance of a single N–H stretching mode, in contrast to the spectra of **CYT** or the parent amino acids, supports successful conjugation. Differences between the spectra of the two derivatives arise mainly from the vibrations associated with sp^2^-hybridized C–H bonds, which are significantly more intense for compound **5**, due to the presence of the phenyl ring.

In the “fingerprint” region, the spectrum of compound **5** (Figure S13) shows a strong band characteristic of aromatic ring ν(C–H) bending vibrations, whereas in that of the alanine derivative **4**, only medium-intensity alkene-related bending modes are observed. Analysis of the double-bond region (1000–1800 cm^−1^; Figure S14) reveals bands characteristic of squaric ring incorporation and substituent differentiation.

In the spectrum of compound **5** (Figure S13), these absorptions partially overlap with aromatic and alkene ν(C=C) stretching modes, resulting in increased band intensity.

Overall, the crystallographic and spectroscopic results consistently confirm the structural integrity and conformational preferences of the synthesized squaramide derivatives in both the solid state and solution.

The solid-state structures of the synthesized squaramides were elucidated by single-crystal X-ray diffraction (Figure 1, Figure 2, Figure 3, Figure 4, Figure 5, Figure 6, Figure 7, Figure 8, Figure 9, Figure 10, Figure 11, Figure 12, Figure 13, Figure 14, Figure 15 and Figure 16). Both compounds crystallize in the orthorhombic space group *P*2_1_2_1_2_1_ with four molecules per unit cell. The analysis confirms retention of the stereogenic centers originating from both the CYT scaffold and the amino acid residues. In both cases, the preferred chair–chair conformation of the alkaloid framework is preserved, while steric effects influence the spatial orientation of the substituents relative to the squaric plane.

Spectroscopic characterization by FT-IR, ESI-MS, and NMR confirmed the formation of the final amino acid ester derivatives (**4** and **5**). Comparative spectroscopic analysis employing the previously reported squaramide **8**, containing a primary amide (–NH_2_, Figure 22) group at the CYT–squaramide ring, as a reference compound [30], facilitated assignment of selected NMR features related to conformational behavior and intermolecular interactions, in agreement with the structural information obtained from X-ray diffraction.

## 3. Materials and Methods

### General

Products of the reactions were purified using silica gel RediSep^®^ Rf columns on CombiFlash Rf+ Lumen Flash Chromatography System with integrated ELS and UV detectors (Teledyne Isco, Lincoln, NE, USA). The solvents used in flash chromatography were of HPLC grade (ChemSolve, Łódź, Poland): dichloromethane, methanol, acetonitrile, DMSO, anhydrous MgSO_4_, and aq. NH_4_OH (POCH, Gliwice, Poland) were used as received. Solvents were removed using a rotary evaporator. 3,4-Dimetoxy-cyklobuten-1,2-dione, 99% purity; (dimethyl squarate **A**, TH.Geyer, Warszawa, Poland); 3,4-Diethoxy-3-cyclobutene-1,2-dione (Chemat, Gdańsk, Poland).

Electrospray ionization (ESI) measurements with positive mode detection were performed using the PurIon Mass Spectrometer Model L (Teledyne ISCO, Lincoln, NE, USA). The mass range for ESI experiments was from *m*/*z* = 100 to *m*/*z* = 1000. The concentration of the studied compounds in final solutions used for the ESI measurement was 5 × 10^−5^ mol dm^−3^. The samples were infused directly into the ESI source. The major parameters were set as follows: Source: ESI + 3.5 kV 350 °C, Capillary: 150 V 300 °C, Offset: 25 V, Span: 0 V, full scan ranging 100−1000 (*m*/*z*). Additionally, a Waters/Micromass (Manchester, UK) ZQ mass spectrometer (MassLynx V4.0) was used. The samples were prepared in methanol or in a methanol–water solution. The concentration of the samples analyzed was 3.6 d 10^−5^ M (1:1 ratio), which is typical of ESI, unless indicated otherwise.

Fourier Transform Infrared spectra of the studied compounds were recorded with the ATR technique, using a Jasco FT-IR spectrometer (ABL&E-JASCO Polska Sp. z o.o., Kraków, Poland). Intensity of the laser is of class 3R, according to the class division of the laser apparatus by IEC 60825-1:2007 (JIS C 6802:2011) or was recorded in KBr tablets on an IFS 113 v FT-IR spectrophotometer (Bruker, Karlsruhe, Germany) equipped with a DTGS detector; wavenumbers (cm^−1^) resolution 2 cm^−1^, NSS = 64.

The NMR spectra of the products were recorded on a Bruker AvanceNEO 600 (Bruker BioSpin GmbH, Ettlingen, Germany). The ^1^H NMR measurements were carried out at the operating frequency **of** 600.1 MHz, while the ^13^C NMR was at 151.2 MHz at 293.0 K. No window function or zero filling was used. ^1^H NMR spectra are reported in chemical shifts downfield (D_2_O δ 4.79 ppm and DMSO-d_6_ at 2.54 ppm) using the respective residual solvent peak as internal standard and TMS. Line broadening parameters were 0.5 or 1.0 Hz, while the error of the chemical-shift value was 0.1 ppm. ^1^H NMR spectra are described as follows: chemical shift (δ, ppm), integration and multiplicity (s = singlet, d = doublet, dd = doublet of doublets, m = multiplet). ^13^C NMR spectra are reported in chemical shifts downfield from the respective residual solvent peak as internal standard DMSO-d_6_ at 40.45 ppm and TMS (0.0 ppm). Line broadening parameters were 0.5 or 1.0 Hz, while the error of the chemical shift value was 0.1 ppm.

X-ray single-crystal data collection. The X-ray intensity data for selected single crystals were collected using graphite monochromatic MoKα radiation on an Xcalibur four-circle κ geometry diffractometer with an Atlas two-dimensional area CCD detector 600 (Rigaku, Yarnton, Oxfordshire, UK). Data collections, cell refinements, integration, correction for Lorenz and polarization effects and absorption corrections were performed using the CrysAlisPro 1.171.42.93a program system [38]. Using Olex2 [39], the structure was solved by the direct methods using SHELXT [40] and refined with the SHELXL-2018/3 program [41]. The hydrogen atoms joined to carbon atoms were introduced in their geometrical positions and treated as rigid with U_iso_ = 1.2U_eq_ (C) for aromatic carbons and U_iso_ = 1.2U_eq_ (C) for other carbons. The positions of the hydrogen atoms of morpholine molecules were refined with U_iso_ = 1.5U_eq_ (C), other carbons joined H. Details of the data collection parameters, crystallographic data and final agreement parameters are collected in Table 1. Diamond 3.0 program [42] was used for visualization of the structure. Refinement of the crystal structure of **3·DMSO** showed that the DMSO solvent molecule in the crystal exhibits slight disorder on the S atoms (C and O atoms are common to both DMSO orientations) with occupancy of 0.949(3) and 0.051(3).

Synthesis of the squaramate **1**

**CYT** (0.190 g, 1 mmol, 1.0 equiv.) and dimethyl squarate **A** (0.213 g, 1.5 mmol) were dissolved separately in methanol (10 mL each) and preheated at ~70 °C. Then the solutions were combined and stirred overnight. After the solvent was removed under reduced pressure, the crude residue was dissolved in water (5 mL) and washed with diethyl ether (3–4 × 20 mL). The aqueous phase was extracted with dichloromethane (4 × 30 mL), and the combined organic layers were dried over anhydrous MgSO_4_ and concentrated. Recrystallization from acetonitrile afforded amido ester **1** as a white solid.

Chemical Formula: C_16_H_16_N_2_O_4_, MW = 300.14; ESI-MS: [M + Na]^+^ = 323; [2M + H]^+^ = 601; [2M + Na]^+^ = 623. FT-IR (ATR, diamond disk) ν (cm^−1^): 3087, 3045, 2960, 1798, 1700, 1648, 1596, 1542, 1483, 1399, 1262, 1030, 822. FT-IR KBr pellet technique—Figure 21. ^1^H NMR (600 MHz, D_2_O) δ, ppm 2.06–2.17 (dd, 2H); 2.60 (m, 1H); 3.35 (m, 1H); 3.51–3.61 (m, 2H); 3.73–3.90 (m, 2H); 4.07/4.26 (s, 3H, -OMe); 4.15 (d, 1H); 4.44 (d, 1H); 6.40–6.45 (m, 2H); 7.45–7.56 (ddd, 1H). ^13^C NMR (150.9 MHz, D_2_O) δ, ppm 26.9; 30.5; 38.0; 52.0; 56.3/56.8; 58.0/58.6; 63.7/64.1; 112.7; 118.9/119.0; 144.0/144.3; 151.5; 167.4; 173.7; 179.1/179.4; 185.7/185.8; 190.4/190.6 (Supplementary Figures S1–S5).

Synthesis of the squaramate **2** [30]

Synthesis of the conjugate **3** and **3·DMSO**

Cytisine (**CYT**, 0.380 g, 2 mmol), previously isolated from the seeds of *Laburnum anagyroides*, was used for the synthesis. Squaric acid (0.456 g, 4 mmol) was employed, maintaining a molar ratio of 1:2. Squaric acid was dissolved in DMF (5 mL), while cytisine was dissolved in DCM (5 mL). The resulting solutions were combined, followed by the addition of toluene (5 mL). The reaction mixture was refluxed under a condenser for 2 h. After completion of the reaction (monitored by TLC), the solvents were removed under reduced pressure. The crude white powder product (0.56 g) was purified by recrystallization.

A portion of the white solid was dissolved in hot isopropanol. After separation of the undissolved residue, the solution was left to crystallize, affording the first crop of crystals of compound **3**. The remaining solid was subsequently dissolved in hot DMSO, from which a second crop of crystals, corresponding to the solvate **3·DMSO**, was obtained. Combined, both crops afforded the desired product as white crystals in 62% yield (0.35 g).

Chemical formula: C_15_H_14_N_2_O_4_, MW = 286.29, ESI-MS: [M + H]^+^ = 287; [M + Na]^+^ = 309; [2M + H]^+^ = 573; [2M + Na]^+^ = 595 (Supplementary Figure S4). FT-IR KBr pellet technique ν (cm^−1^): 2432, 1797, 1689, 1591—Figure 22. ^1^H NMR (600 MHz, DMSO-d_6_) δ, ppm 2.07–1.97 (m, 2H), 2.52 (bm 1H), 3.23 (bs, 1 H), 3.47 (m, 1H), 3.54 (dd, 1H), 3.68 (ddd, 1H), 3.94 (d, 1H), 4.11 (d, 1H), 4.26 (d, 1H), 6.16 (dd, 1H), 6.20 (dd, 1H), 7.32 (dd, 1 H). ^13^C NMR (150.9 MHz, DMSO-d_6_): δ, ppm 25.2, 28.0, 35.2, 49.3, 53.5, 55.0, 106.3, 117.2, 140.0, 149.7, 163.1, 172.8, 184.1, 185.4/185.4 (Supplementary Figures S6–S8).

Synthesis of the squaramate **4** and **5**

Squaramate **1** (150 mg, 0.5 mmol, 1.0 equiv) was dissolved in hot methanol (10 mL) and added to a methanolic solution (5 mL) of the relevant amino acid methyl ester hydrochloride (2.0 equiv). Triethylamine (2 drops) was added, and the reaction mixture was stirred at ambient temperature for 24 h. After completion, the solvent was removed under reduced pressure. Compound **4** was purified directly by flash column chromatography using CMA eluent (dichloromethane/methanol/aq. NH_4_OH in a ratio of 90:10:1). The crude residue with compound **5** was subjected to neutral liquid–liquid extraction with diethyl ether and water, followed by water/dichloromethane partitioning, drying, and solvent removal prior to flash chromatography. Both compounds, after recrystallization from acetonitrile, were obtained as white, needle-shaped crystals.

Squaramide **4**. Chemical Formula: C_19_H_21_N_3_O_5_, MW = 371.39. ESI-MS: [M]^+^ = 371; [M + Na]^+^ = 394; [2M]^+^ = 742; [2M + Na]^+^ = 765. FT-IR (ATR, diamond disk) ν (cm^−1^): 3246, 2945, 1792, 1729, 1679, 1646, 1585, 1505, 1436, 1347, 1271, 1058, 804. ^1^H NMR (600 MHz, DMSO-d_6_) δ, ppm 1.38–1.39 (d, 3H); 1.98–2.06 (dd. 2H); 2.49 (m, 1H); 3.21 (t, 1H); 3.39/3.65 (s, 3H); 3.48–3.51 (m, 4H); 3.67–3.71 (d, 1H); 3.98–4.01 (d, 1H); 4.87 (broad, 1H); 6.12–6.13 (d, 1H); 6.20–6.21 (d, 1H); 7.29–7.32 (m, 1H), 7.79 (broad, 1H). ^13^C NMR (150.9 MHz, DMSO-d_6_): δ, ppm 18.2; 24.5; 27.0; 33.9; 48.1; 51.4; 52.3; 52.9; 54.1; 105.3; 116.1; 138.9; 148.6; 162.1; 166.5; 166.3; 172.2; 182.0; 182.2. (Supplementary Figures S9, S10, S13 and S14).

Squaramide **5**. Chemical Formula: C_25_H_25_N_3_O_5,_ MW = 447,48. ESI-MS: [M + Na]^+^ = 470; [2M + Na]^+^ = 917. FT-IR (ATR, diamond disk) ν (cm^−1^): 3206, 3058, 2925, 1789, 1734, 1660, 1647, 1550, 1509, 1435, 1348, 1282, 1252, 1037, 698. ^1^H NMR (600 MHz, DMSO-d_6_) δ, ppm 1.97–2.04 (dd, 2H); 2.24–2.26 (m, 1H); 2.57–2.59 (d, 1H); 2.97–2.99 (m, 1H); 3.14–3.20 (m, 2H); 3.45–3.50 (m, 2H); 3.68 (s, 3H); 3.75 (d, 1H); 3.97–3.99 (d, 1H); 5.08–5.12 (broad, 1H); 6.11–6.12 (d, 1H); 6.23–6.25 (d, 1H); 7.17–7.30 (m, 9H); 7.93 (broad, 1H). ^13^C NMR (150.9 MHz, DMSO-d_6_): δ, ppm 24.4; 26.9; 34.0; 48.2; 52.3; 52.8; 55.4; 57.1; 105.0; 116.1; 126.5; 128.2; 128.3; 129.1; 129.8; 136.5–136.8; 138.8; 148.8; 162.0; 165.3; 165.9–166.2; 171.1; 181.7; 182.0. (Supplementary Figures S11–S14).

Synthesis of co-crystals **6** and **7**

Co-crystals **6** and **7** were obtained by combining the respective squaramide ester and squaramide acid components in a 1:1 molar ratio, followed by recrystallization from DMSO. Recrystallization of CytSQ-OMe (**1**) with CytSQOH (**3**) afforded high-quality single crystals of co-crystals suitable for X-ray diffraction analysis. ESI-MS of dissolved crystals is presented in Figure 18. Similarly, recrystallization of CytSQ-OEt (**2**) with CytSQOH (**3**) under the same conditions afforded high-quality single crystals of co-crystal **7** suitable for X-ray diffraction analysis.

DFT calculation. Molecular geometries of the investigated compounds were optimized using the Gaussian09 software package [43]. The optimization process was conducted without any symmetry constraints to achieve the most stable structure. The Becke, three-parameter, Lee-Yang-Parr (B3LYP) exchange-correlation functional was employed, along with the 6-31G(d,p) basis set for all atoms [44,45]. The optimized molecular structure was visualized using the GaussView program Version 5.0.8 [46], enabling a detailed examination of bond lengths, angles, and overall molecular conformation. Additionally, the three-dimensional molecular electrostatic potential maps were generated for elucidation of intermolecular interactions and their mutual organization in crystals.

## 4. Conclusions

We report the synthesis and comprehensive structural elucidation of cytisine–squaramide conjugates incorporating single amino acid units, uncovering a previously unrecognized interplay between ester hydrolysis and supramolecular assembly in this system. We demonstrate that the formation of amido-acid species is not a side reaction but an intrinsic feature of cytisine squaramate chemistry, governed by a dynamic ester–acid equilibrium under mild conditions.

A key advance of this work is the identification of the persistent insoluble “by-products” as well-defined co-crystals **6** and **7** composed of amido ester (**1** or **2**) and amido-acid (**3**) components. Their unambiguous structural characterization by single-crystal X-ray diffraction resolves the origin of long-standing purification challenges and provides direct experimental evidence of spontaneous supramolecular organization driven by complementary hydrogen bonding and electrostatic interactions. This mechanistic insight enabled rational optimization of purification protocols, allowing reliable access to squaramate and squaramide derivatives.

Across all investigated structures (**1**–**7**), the cytisine scaffold consistently preserves its rigid tricyclic quinolizidine framework, confirming its conformational robustness. In contrast, the squaramide–amino acid fragments exhibit pronounced conformational flexibility, reflected in a wide range of dihedral angles relative to the pyridone ring. These variations are strongly influenced by the molecular environment, including solvent effects, substituent identity aliphatic (**4**) vs. aromatic (**5**), and co-crystal formation (**6** and **7**). Comparison with DFT-optimized geometries reveals a systematic tendency toward larger dihedral angles in the gas phase, highlighting the critical role of intermolecular interactions in stabilizing more compact conformations in the solid state. The supramolecular organization is governed primarily by directional hydrogen bonding. In compound **3** and in co-crystals **6** and **7**, the O–H···O interactions dominate, leading to one-dimensional chains or helical motifs, whereas in derivatives **4** and **5**, the N–H···O hydrogen bonds define the assembly. Despite differences in substituents and crystal systems, the resulting architectures remain predominantly one-dimensional, with higher-order packing controlled by van der Waals interactions and weak C–H···O contacts. The absence of strong interchain hydrogen bonding underscores the modular nature of these systems and suggests that subtle structural modifications can effectively tune both molecular conformation and crystal packing.

These findings redefine the synthetic and supramolecular landscape of cytisine–squaramide chemistry and provide a mechanistically informed platform for future functional design. The integration of a biologically validated alkaloid scaffold with tuneable squaramide and amino acid motifs positions these systems as promising candidates for applications in medicinal chemistry, molecular recognition, and adaptive supramolecular materials.

## Figures and Tables

**Table 1:** Crystallographic data and final refinement parameters for investigated crystals.

	3·DMSO	3	4	5	6	7
Formula	C_17_H_20_N_2_O_5_S	C_15_H_14_N_2_O_4_	C_19_H_21_N_3_O_5_	C_25_H_25_N_3_O_5_	C_31_H_30_N_4_O_8_	C_32_H_32_N_4_O_8_
Mol. weight	364.41	286.28	371.39	447.48	586.59	600.61
T (K)	100(2)	100(2)	295(2)	295(2)	295(2)	295(2)
Crystal system	monoclinic	monoclinic	orthorhombic	orthorhombic	monoclinic	monoclinic
Space group	P2_1_	P2_1_	P2_1_2_1_2_1_	P2_1_2_1_2_1_	P2_1_	P2_1_
*a* (Å)	8.5773(4)	7.3355(2)	7.1836(3)	6.9920(3)	7.6070(3)	7.6761(3)
*b* (Å)	8.4952(5)	14.4095(4)	11.0542(5)	9.3258(5)	14.1981(5)	14.3340(4)
*c* (Å)	11.7978(6)	12.0799(3)	22.4305(8)	33.231(2)	13.1188(5)	13.1636(5)
*β* (°)	101.924(5)	94.953(2)			91.282(4)	90.203(3)
*V* (Å^3^)	841.11(8)	1272.09(6)	1781.18(13)	2166.9(2)	1416.54(9)	1448.37(9)
Z	2	4	4	4	2	2
*F* (000)	384	600	784	944	616	632
*D_cal_* (g·cm^−1^)	1.439	1.495	1.385	1.372	1.375	1.377
θ range(°)	2.69–29.30	2.78–29.50	2.59–29.38	2.51–29.35	2.69–20.43	
μ (mm^−1^)	0.224	0.110	0.102	0.097	0.101	0.100
Crystal size (mm)	0.32 × 0.27 × 0.13	0.29 × 0.25 × 0.11	0.30 × 0.24 × 0.21	0.32 × 0.26 × 0.22	0.32 × 0.25 × 0.10	0.25 × 0.1 × 0.07
T_min_/T_max_	0.9574/1.00	0.9826/1.00	0.9816/1.00	0.9584/1.00	0.9848/1.00	0.9328/1.00
Total/unique/obs. refls (*F*^2^ > 2*σ*(*F*^2^))	11,223/4041/3528	28,221/6412/5826	33,703/4598/2812	13,733/5257/3753	20,424/6767/4608	26,866/7138/4191
*R_int_*	0.0213	0.0308	0.0749	0.0329	0.0268	0.0440
*R* [*F*^2^ > 2*σ*(*F*^2^)] *^a^*	0.0389	0.0356	0.0556	0.0550	0.0468	0.0571
*wR* [*F*^2^ all refls] *^a^*	0.0845	0.0811	0.0929	0.1297	0.1016	0.1329
*S*	1.002	1.002	1.002	1.046	1.004	1.001
Flack parameter	−0.05(4)	0.2(3)	0.4(7)	−0.7(6)	0.2(5)	0.1(5)
Δρ_max_, Δρ_min_ (eÅ^−3^)	+0.300, −0.227	+0.265, −0.197	+0.159, −0.183	+0.162, −0.216	+0.142, −0.139	+0.186, −0.231

*^a^ R* = Σ ||*F*_o_| − |*F*_c_||/Σ*F*_o_, *wR* = {Σ [*w*(*F*_o_^2^ − *F*_c_^2^)^2^]/Σ*wF*_o_^4^}^½^; *w*^−1^ = σ^2^(*F*_o_^2^) + (*aP*)^2^ + *bP* where *P* = (*F*_o_^2^ + 2*F*_c_^2^)/3. The *a* and *b* parameters are 0.0396 and 0.1347 for **3**·**DMSO**, 0.400 and 0.3044 for **3**, 0.0358 and 0.0467 for **4**, 0.0641 and 0.0346 for **5**, 0.0434 and 0.0891 for **6**, 0.0589 and 0.0678 for **7**.

## Data Availability

The original contributions presented in this study are included in the article/Supplementary Material. Further inquiries can be directed to the corresponding author(s).

## Supplementary Materials

The following supporting information can be downloaded at: https://www.mdpi.com/article/10.3390/molecules31111961/s1. Additional materials containing ESI-MS, ^1^H NMR and ^13^C NMR and the DFT-optimized parameters all compounds CCDC No. **2552221**, **2552222**, **2552223**, **2552224**, **2552225** and **2552226** contain the supplementary crystallographic data in cif format for crystals **3·DMSO**, **3**, **4**, **5**, **6**, and **7**, respectively. These data can be also obtained free of charge from the Cambridge Crystallographic Data Centre, 12 Union Road, Cambridge CB2 1EZ, UK; fax: (+44) 1223-336-033; or e-mail: deposit@ccdc.cam.ac.uk.

## References

[1] Giordanetto F., Tyrchan C., Ulander J. (2017). Intramolecular Hydrogen Bond Expectations in Medicinal Chemistry. ACS Med. Chem. Lett..

[2] Harrold M.W., Zavod R.M. (2024). Functional Group Characteristics and Roles. Basic Concepts in Medicinal Chemistry.

[3] Zatoński W., Janik-Koncewicz K., Stępnicka Z., Zatońska K., Połtyn-Zaradna K., Herbeć A. (2020). History of Smoking Cessation Treatment in Poland—The Strengthening Role of Cytisine as the Most Effective and Safe Pharmacotherapy. J. Health Inequal..

[4] Wang X., Yang J., Huang P., Wang D., Zhang Z., Zhou Z., Liang L., Yao R., Yang L. (2024). Cytisine: State of the Art in Pharmacological Activities and Pharmacokinetics. Biomed. Pharmacother..

[5] Morales-Perez C.L., Noviello C.M., Hibbs R.E. (2016). X-Ray Structure of the Human A4β2 Nicotinic Receptor. Nature.

[6] Knox H.J., Rego Campello H., Lester H.A., Gallagher T., Dougherty D.A. (2022). Characterization of Binding Site Interactions and Selectivity Principles in the A3β4 Nicotinic Acetylcholine Receptor. J. Am. Chem. Soc..

[7] Blom A.E.M., Campello H.R., Lester H.A., Gallagher T., Dougherty D.A. (2019). Probing Binding Interactions of Cytisine Derivatives to the A4β2 Nicotinic Acetylcholine Receptor. J. Am. Chem. Soc..

[8] Ruseva N., Sbirkova-Dimitrova H., Atanasova M., Marković A., Šmelcerović Ž., Šmelcerović A., Bakalova A., Cherneva E. (2023). Synthesis and DNase I Inhibitory Properties of New Squaramides. Molecules.

[9] Priyanka N., Bila G., Mavileti S.K., Bila E., Negrych N., Gupta S., Tang L., Bilyy R., Pandey S.S., Kato T. (2024). A Biocompatible NIR Squaraine Dye and Dye-Antibody Conjugates for Versatile Long-Term in Vivo Fluorescence Bioimaging. Mater. Adv..

[10] Bacher E.P., Twiringiyimana R., Rodriguez K.X., Wilson R.A., Bodnar A.K., O’Connell R., Toni T.A., Eckert K.E., Wiest O., Ashfeld B.L. (2023). A Proline-Squaraine Ligand Framework (Pro-SqEB) for Stereoselective Rhodium(II)-Catalyzed Cyclopropanations. Org. Lett..

[11] Marchetti L.A., Kumawat L.K., Mao N., Stephens J.C., Elmes R.B.P. (2019). The Versatility of Squaramides: From Supramolecular Chemistry to Chemical Biology. Chem.

[12] Alegre-Requena J.V., Marqués-López E., Herrera R.P. (2015). One-Pot Synthesis of Unsymmetrical Squaramides. RSC Adv..

[13] Agnew-Francis K.A., Williams C.M. (2020). Squaramides as Bioisosteres in Contemporary Drug Design. Chem. Rev..

[14] Quintana M., Alegre-Requena J.V., Marqués-López E., Herrera R.P., Triola G. (2016). Squaramides with Cytotoxic Activity against Human Gastric Carcinoma Cells HGC-27: Synthesis and Mechanism of Action. MedChemComm.

[15] Palme P.R., Grover S., Abdelaziz R., Mann L., Kany A.M., Ouologuem L., Bartel K., Sonnenkalb L., Reiling N., Hirsch A.K.H. (2025). Design, Synthesis, and Biological Evaluation of Mono- and Diamino-Substituted Squaramide Derivatives as Potent Inhibitors of Mycobacterial Adenosine Triphosphate (ATP) Synthase. J. Med. Chem..

[16] Rotger M.C., Piña M.N., Frontera A., Martorell G., Ballester P., Deyà P.M., Costa A. (2004). Conformational Preferences and Self-Template Macrocyclization of Squaramide-Based Foldable Modules. J. Org. Chem..

[17] Ramalingam V., Domaradzki M.E., Jang S., Muthyala R.S. (2008). Carbonyl Groups as Molecular Valves to Regulate Chloride Binding to Squaramides. Org. Lett..

[18] Nguyen T., Decker A.M., Barrus D.G., Song C.H., Liu J., Gamage T.F., Harris D.L., Li J., Zhang Y. (2025). Development of Squaramides as Allosteric Modulators of the CB 1 Receptor: Synthesis, Computational Studies, Biological Characterization, and Effects against Cocaine-Induced Behavioral Sensitization and Reinstatement in Rats. J. Med. Chem..

[19] Onaran M.B., Comeau A.B., Seto C.T. (2005). Squaric Acid-Based Peptidic Inhibitors of Matrix Metalloprotease-1. J. Org. Chem..

[20] Picci G., Montis R., Lippolis V., Caltagirone C. (2024). Squaramide-Based Receptors in Anion Supramolecular Chemistry: Insights into Anion Binding, Sensing, Transport and Extraction. Chem. Soc. Rev..

[21] Arifian H., Maharani R., Megantara S., Gazzali A.M., Muchtaridi M. (2022). Amino-Acid-Conjugated Natural Compounds: Aims, Designs and Results. Molecules.

[22] Xu Q., Deng H., Li X., Quan Z.S. (2021). Application of Amino Acids in the Structural Modification of Natural Products: A Review. Front. Chem..

[23] Ishida T., Shinada T., Ohfune Y. (2005). Synthesis of Novel Amino Squaric Acids via Addition of Dianion Enolates Derived from N-Boc Amino Acid Esters. Tetrahedron Lett..

[24] Dolev N., Katz Z., Ludmer Z., Ullmann A., Brauner N., Goikhman R. (2020). Natural Amino Acids as Potential Chelators for Soil Remediation. Environ. Res..

[25] Jacob R.H., Afify A.S., Shanab S.M., Shalaby E.A. (2024). Chelated Amino Acids: Biomass Sources, Preparation, Properties, and Biological Activities. Biomass Convers. Biorefin..

[26] Dattatray Shinde S., Kumar Behera S., Kulkarni N., Dewangan B., Sahu B. (2024). Bifunctional Backbone Modified Squaramide Dipeptides as Amyloid Beta (Aβ) Aggregation Inhibitors. Bioorg. Med. Chem..

[27] Elmes R.B.P., Jolliffe K.A. (2015). Amino Acid-Based Squaramides for Anion Recognition. Supramol. Chem..

[28] Tong H., Ali F., Elmes R.B.P. (2024). Amino Acid–Squaramide Conjugates as Anion Binding Receptors. Results Chem..

[29] Wijata A., Grajewska A., Janczak J., Huczyński A. (2025). Synthesis and Structural Characterization of a New Colchicine Monosquarate-Amide Derivative. J. Mol. Struct..

[30] Przybył A.K., Janczak J., Huczyński A. (2025). Synthesis and Structural Analysis of New (–)-Cytisine Squaramides. Molecules.

[31] Przybył A.K., Grzeskiewicz A.M., Kubicki M. (2021). Weak Interactions in the Structures of Newly Synthesized (–)-Cytisine Amino Acid Derivatives. Crystals.

[32] Hadži D. (1965). Infrared Spectra of Strongly Hydrogen-Bonded Systems. Pure Appl. Chem..

[33] Evans J.C. (1962). Narrow Transmission Regions within Broad Infrared Absorption Bands in Solids. Spectrochim. Acta.

[34] Stare J., Mavri J., Ambrožič G., Hadži D. (2000). Strong Intramolecular Hydrogen Bonds. Part I. Vibrational Frequencies of the OH Group in Some Picolinic Acid N-Oxides Predicted from DFT Calculated Potentials and Located in the Infrared Spectra. J. Mol. Struct. THEOCHEM.

[35] Bratos S., Ratajczak H., Viot P. (1991). Properties of H-Bonding in the Infrared Spectral Range. Hydrogen-Bonded Liquids.

[36] Zeegers-Huyskens T., Sobczyk L. (1990). Critical Behaviour of Strong Hydrogen Bonds and the Isotopic Effects. J. Mol. Liq..

[37] Huczyński A., Ratajczak-Sitarz M., Katrusiak A., Brzezinski B. (2010). Crystals of the Kemp’s Triacid Salts. Part V: Structure of Hydrogen-Bonded Complex of Kemp’s Triacid with 7-Methyl-1,5,7-Triazabicyclo [4.4.0]Dec-5-Ene Studied by X-Ray and FT-IR Methods. J. Mol. Struct..

[38] Rigaku Divisions (2023). CrysAlisPro 1.171.42.93a Software System.

[39] Dolomanov O.V., Bourhis L.J., Gildea R.J., Howard J.A.K., Puschmann H. (2009). OLEX2: A Complete Structure Solution, Refinement and Analysis Program. J. Appl. Crystallogr..

[40] Sheldrick G.M. (2015). SHELXT—Integrated Space-Group and Crystal-Structure Determination. Acta Crystallogr. Sect. A Found. Crystallogr..

[41] Sheldrick G.M. (2015). Crystal Structure Refinement with SHELXL. Acta Crystallogr. Sect. C Struct. Chem..

[42] Putz H., Brandenburg K. (2014). DIAMOND.

[43] Frisch M.J., Trucks G.W., Schlegel H.B., Scuseria G.E., Robb M.A., Cheeseman J.R., Scalmani G., Barone V., Mennucci B., Petersson G.A. (2009). Gaussian.

[44] Becke A.D. (1993). Density-Functional Thermochemistry. III. The Role of Exact Exchange. J. Chem. Phys..

[45] Lee C., Yang W., Parr R.G. (1988). Development of the Colle-Salvetti Correlation-Energy Formula into a Functional of the Electron Density. Phys. Rev. B.

[46] Dennington R., Keith T., Millam J. (2009). GaussView.

