# Supplementary material for: Functionalized Cytisine Squaramides: Synthesis, Structural Elucidation, and Co-Crystallization
Source: Molecules. 2026 Jun 4;31(11):1961. doi: 10.3390/molecules31111961 (PMC13257630; doi:10.3390/molecules31111961)
Supplement: Supplementary file 1 [file molecules-31-01961-s001.zip › 5_Supplementary data_AKP_AM_Co-crystalization - FINAL.pdf]

Supplementary data

Functionalized Cytisine Squaramides: Synthesis, Structural Elucidation, and Co-Crystallization

Anna K. Przybył [1,*], Alona Mintianska [1], Adam Huczyński [1] and Jan Janczak [2,*]

[1] Department of Medical Chemistry, Faculty of Chemistry, Adam Mickiewicz University, Uniwersytetu
Poznańskiego 8, 61-614 Poznań, Poland; alona.mintianska@amu.edu.pl (A.M.); adhucz@amu.edu.pl (A.H.)

[2] Institute of Low Temperature and Structure Research, Polish Academy of Sciences, Okólna 2 Str.,
50-422 Wrocław, Poland

* Correspondence: anna.przybyl@amu.edu.pl (A.K.P.); j.janczak@intibs.pl (J.J.)

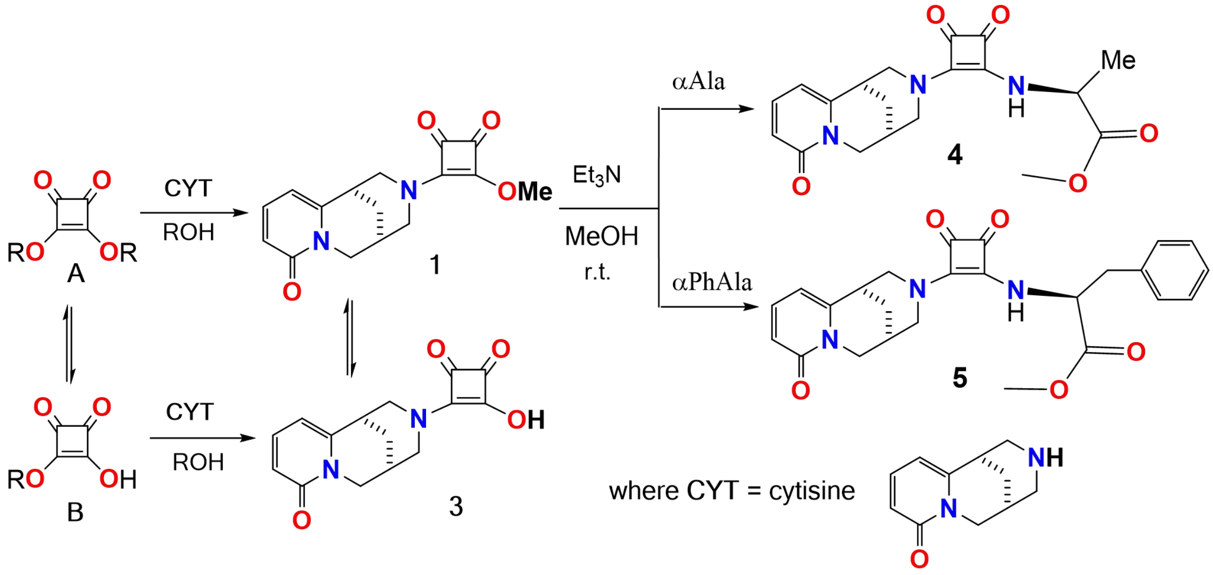

Scheme S1. Synthesis of squaramide-esters (**1** and **3**) and bifunctional squaramides **4** and **5**.

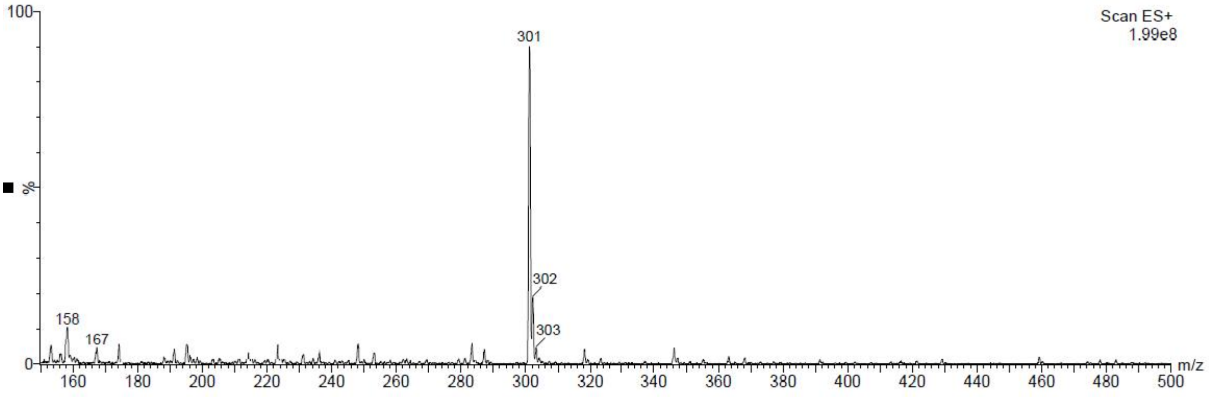

Figure S1. ESI-MS spectrum of **1** (MW 300).

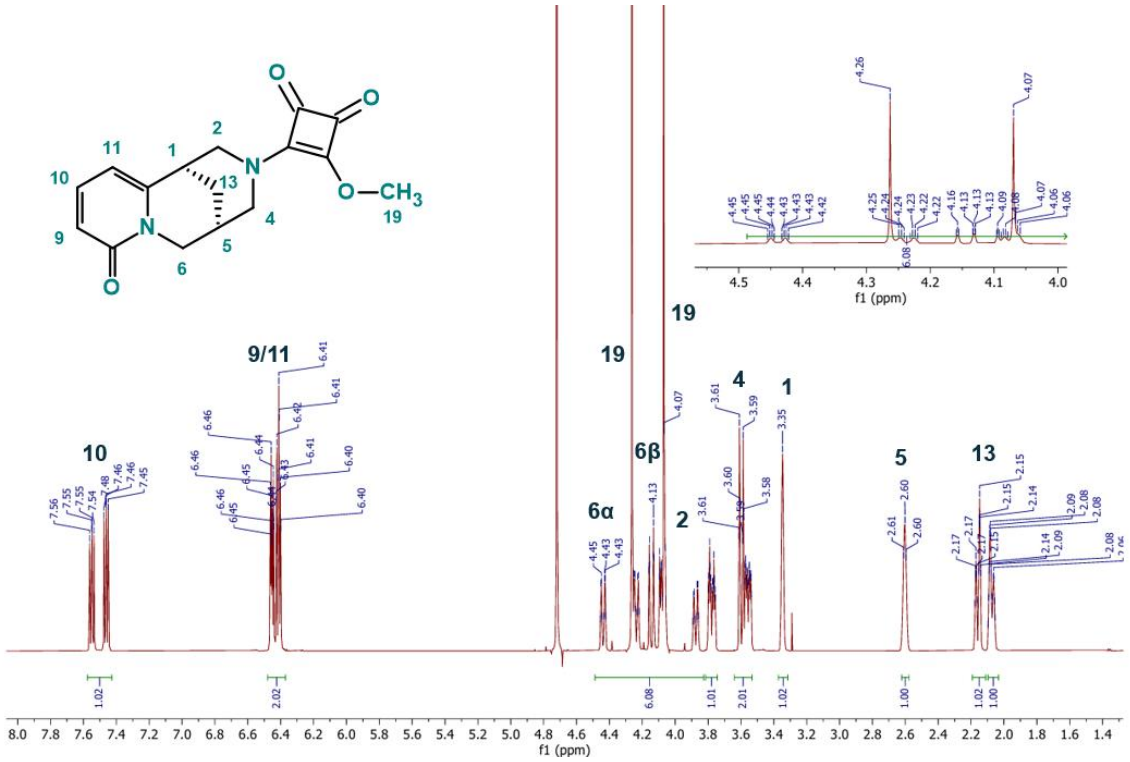

Figure S2. ^{1}H NMR spectrum of **1** (600 MHz, D_2O).

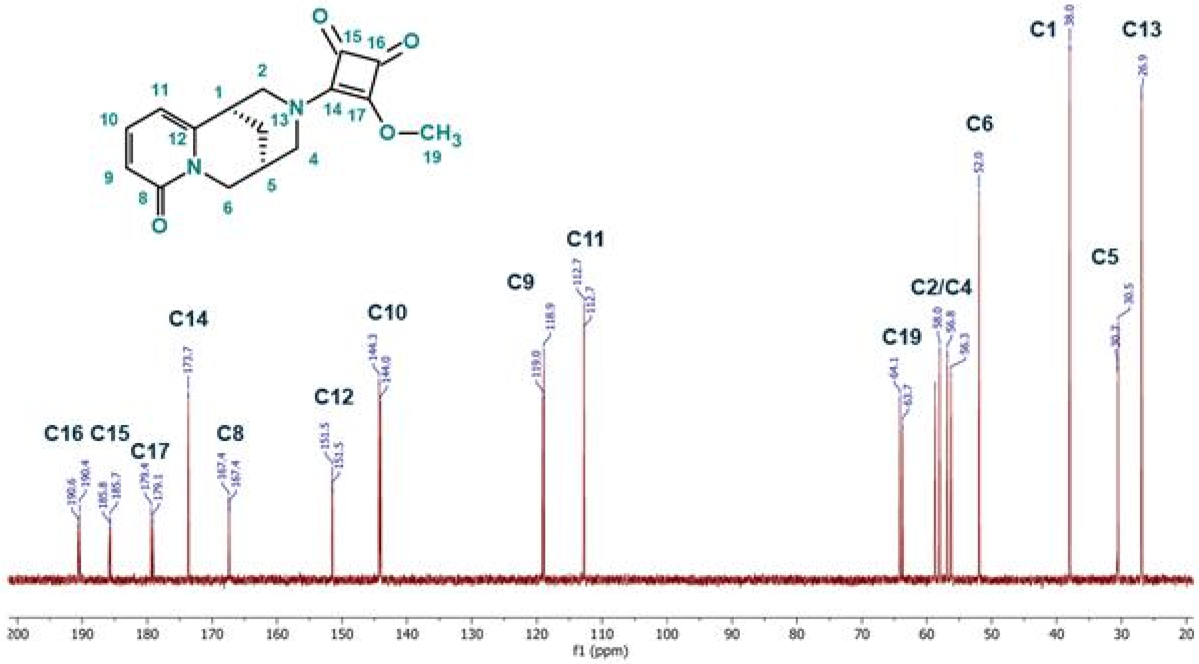

Figure S3. ^{13}C NMR spectrum of **1** (151 MHz, D_2O).

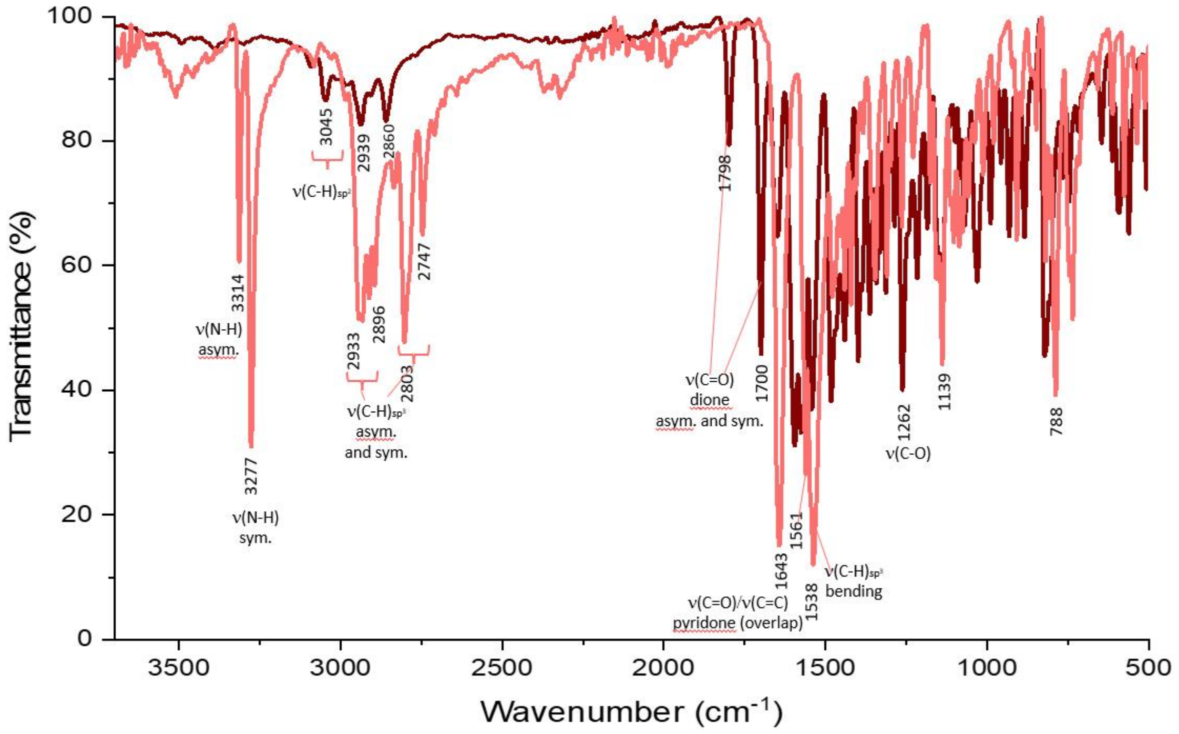

Figure S4. Overlaid FT-IR spectra of **CYT** (pink line) and **1** (maroon line).

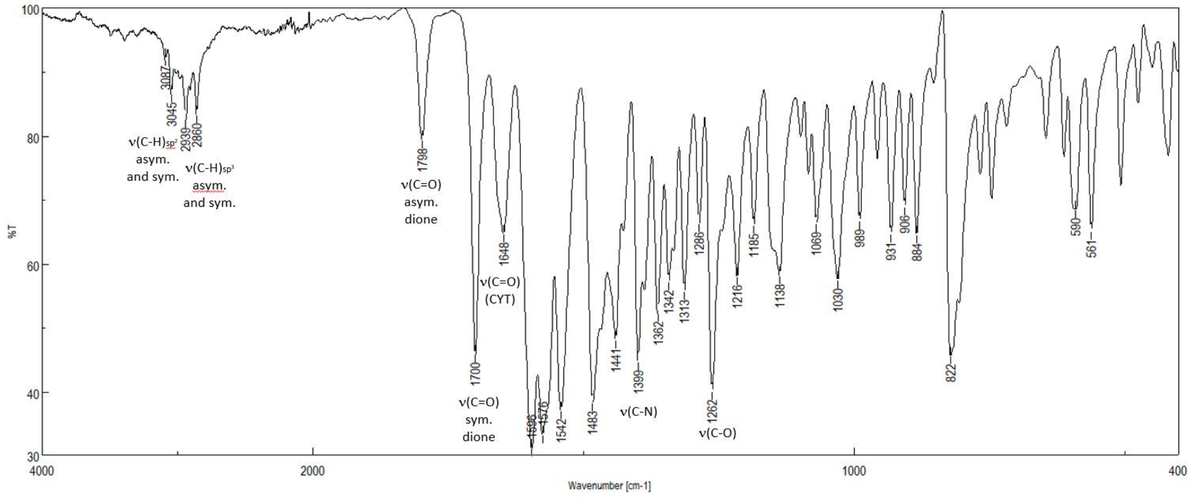

Figure S5. FT-IR spectrum of **1**.

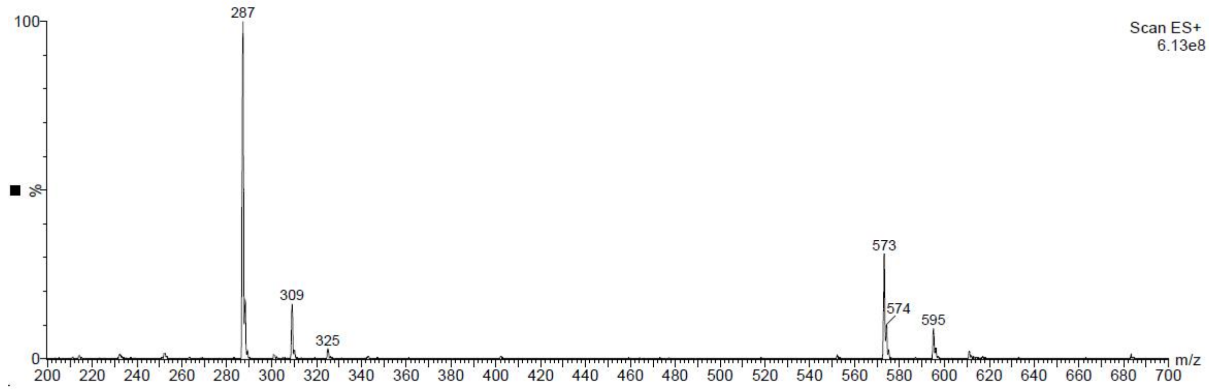

Figure S6. ESI-MS spectrum of squaramate-acid **3** (MW 300).

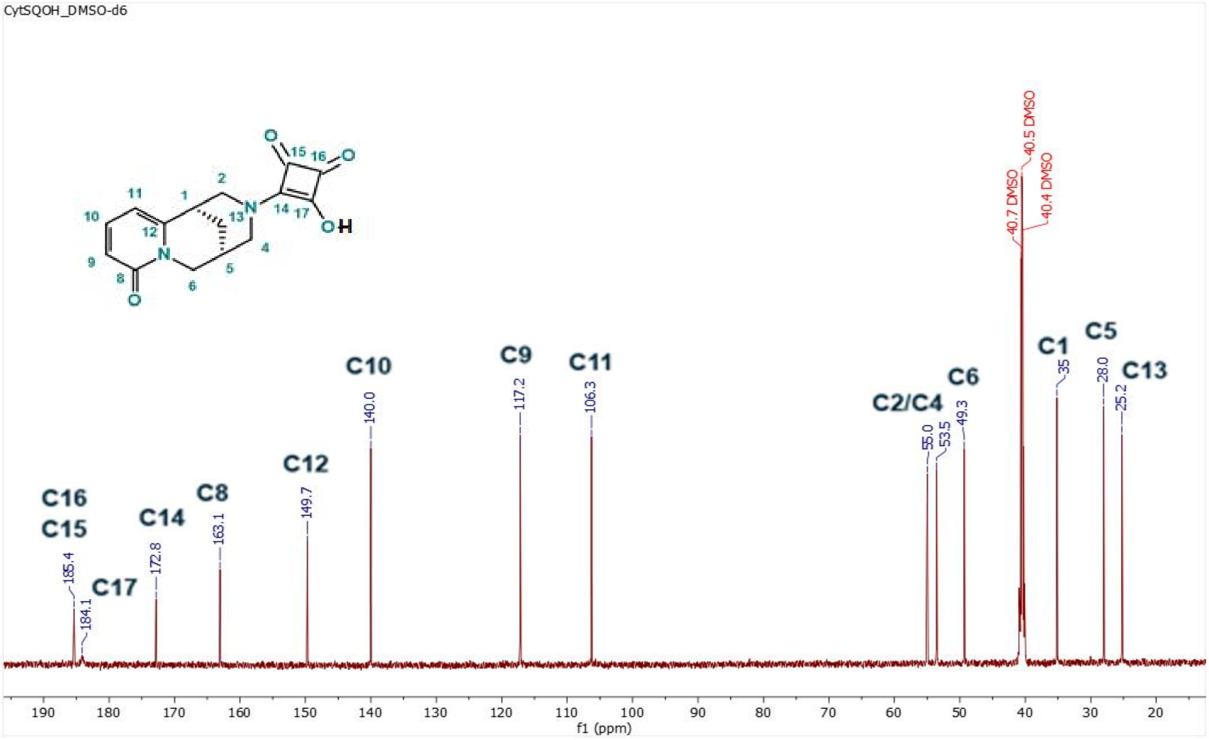

Figure S7. ^{13}C NMR spectrum of **3** (151 MHz, DMSO-d$_6$).

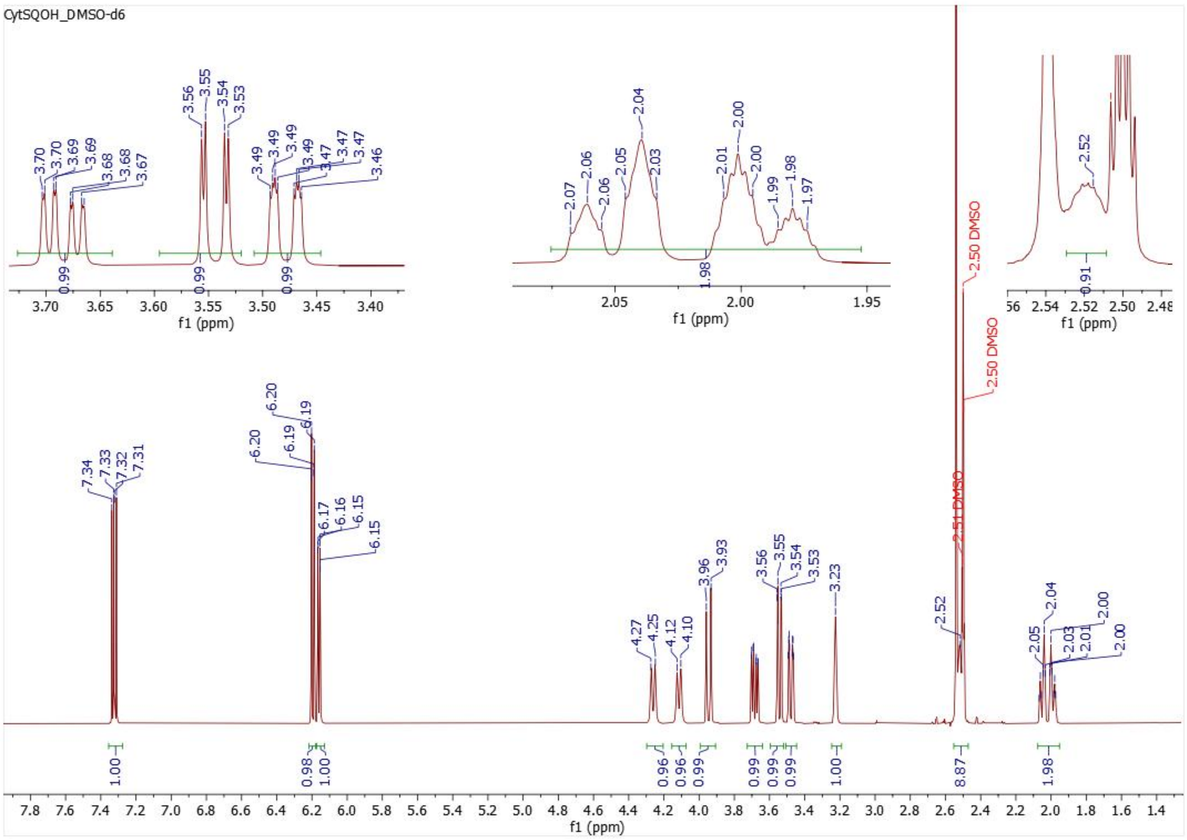

Figure S8. ^{1}H NMR spectrum of **3** (600 MHz, DMSO-d_6).

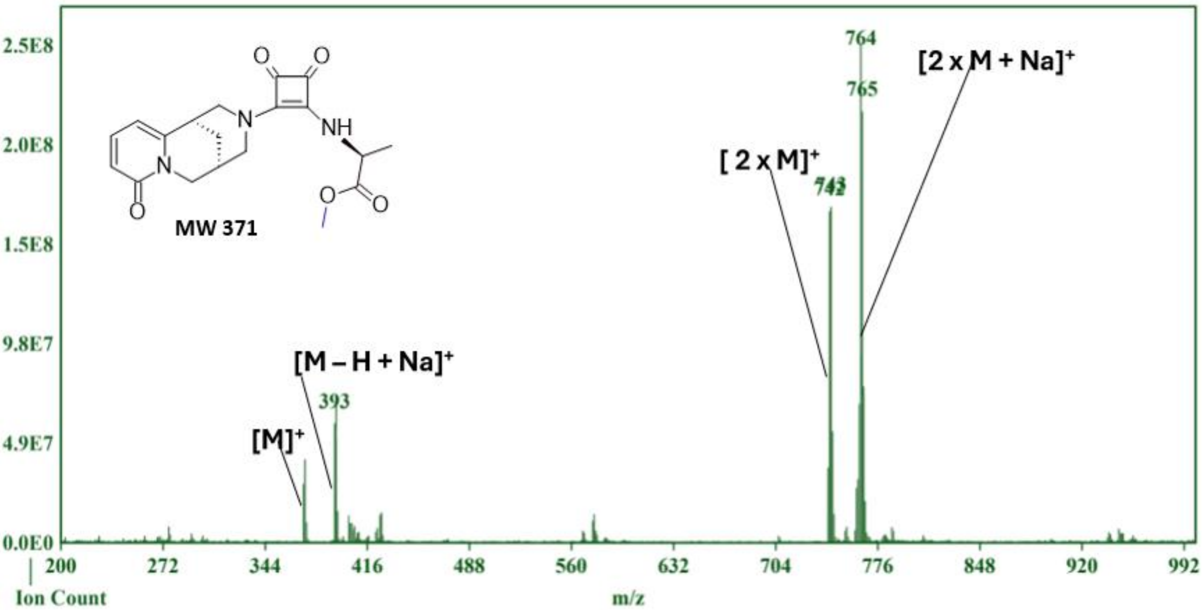

Figure S9. ESI-MS spectrum of **4** (MW 371).

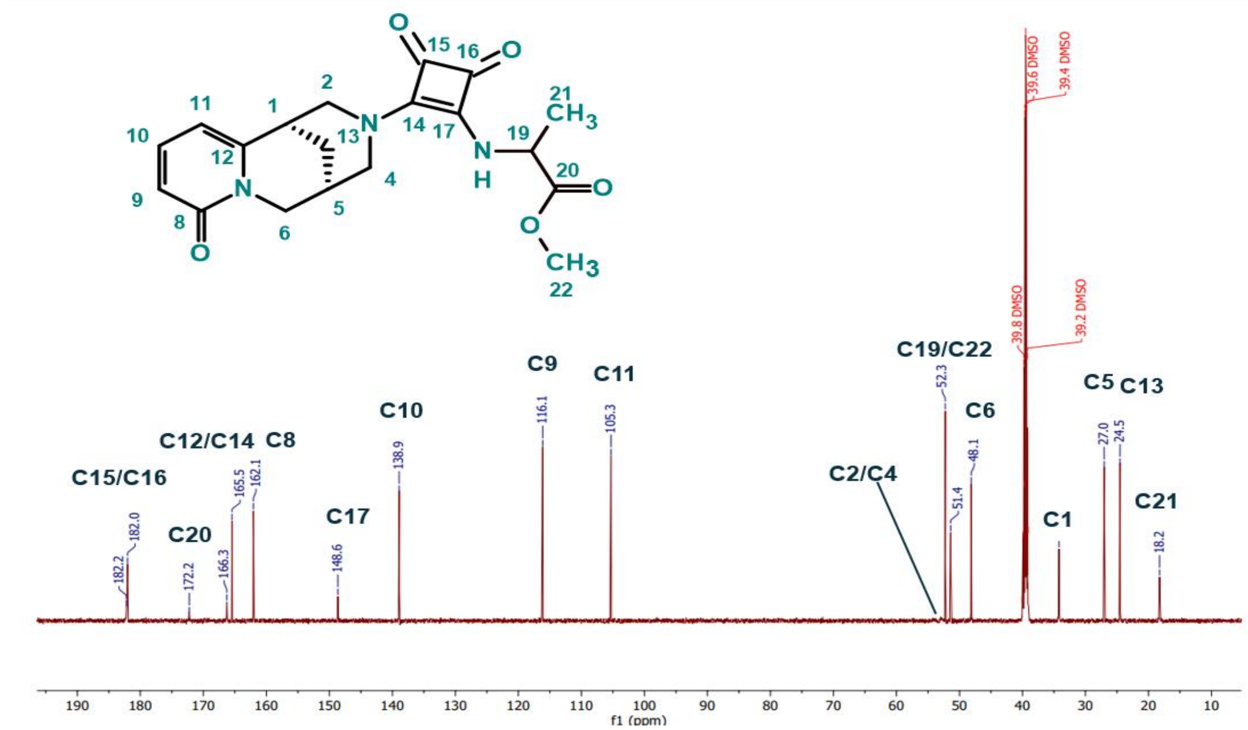

Figure S10. ^{13}C NMR spectrum of **4** (151 MHz, DMSO-d$_6$).

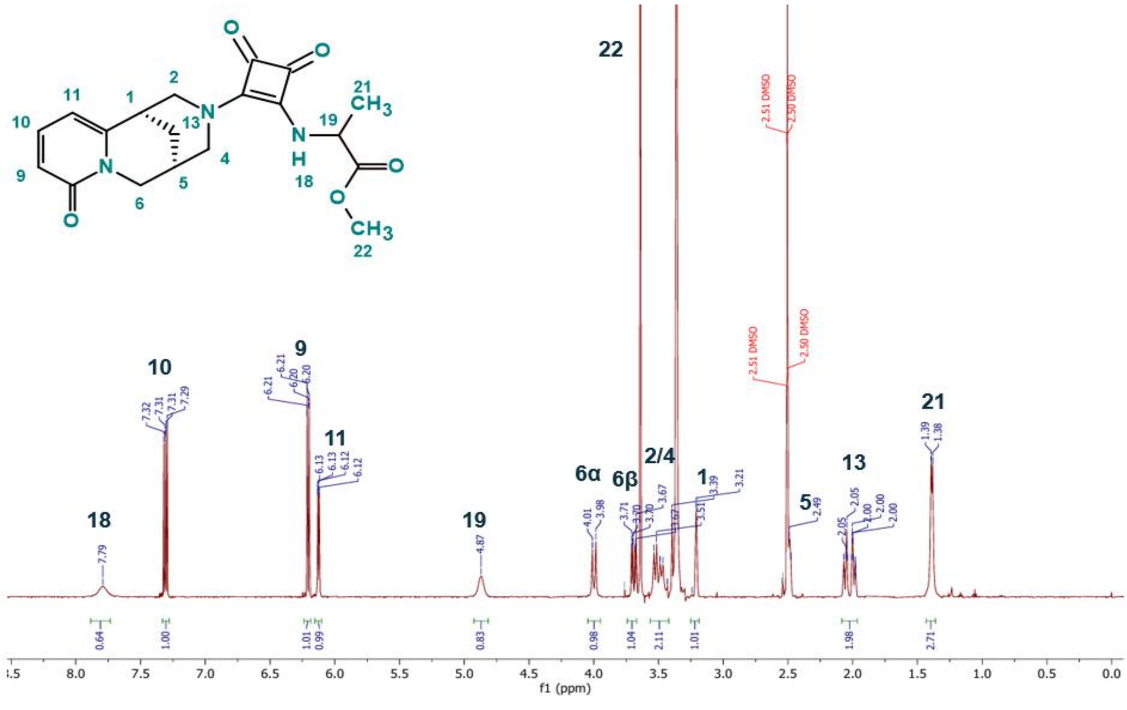

Figure S11. ^{1}H NMR spectrum of **4** (600 MHz, DMSO-d$_6$).

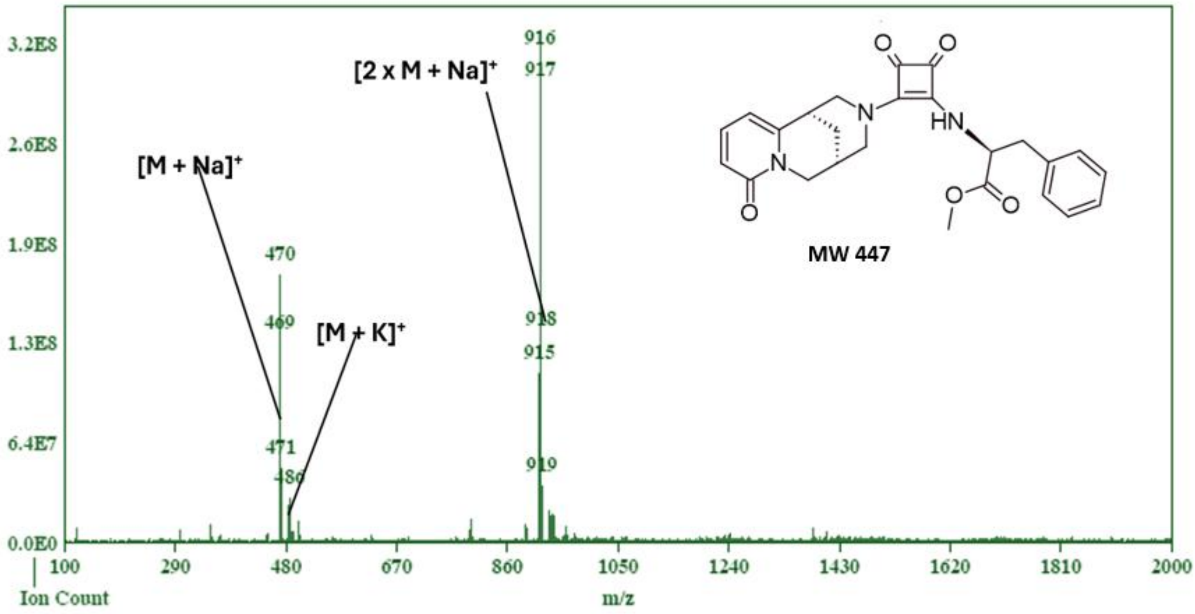

Figure S12. ESI-MS spectrum of **5** (MW 371).

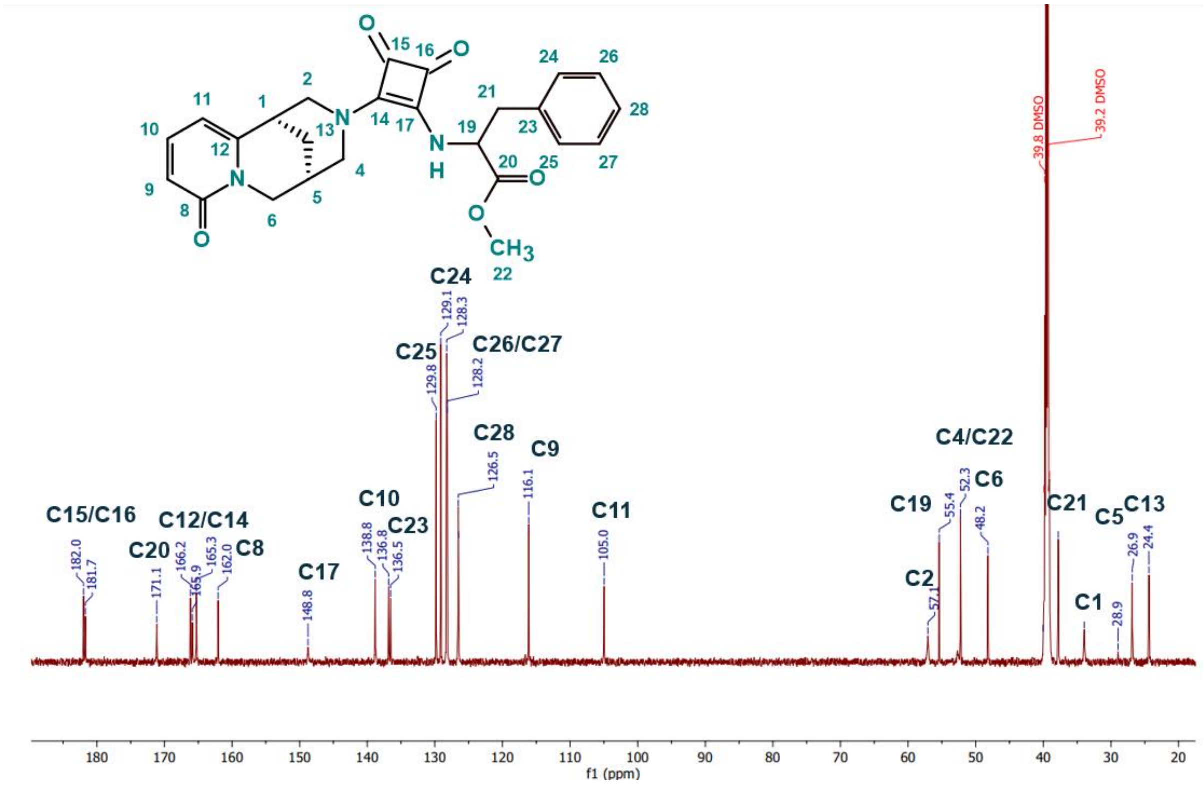

Figure S13. ^{13}C NMR spectrum of **5** (151 MHz, DMSO-d_6).

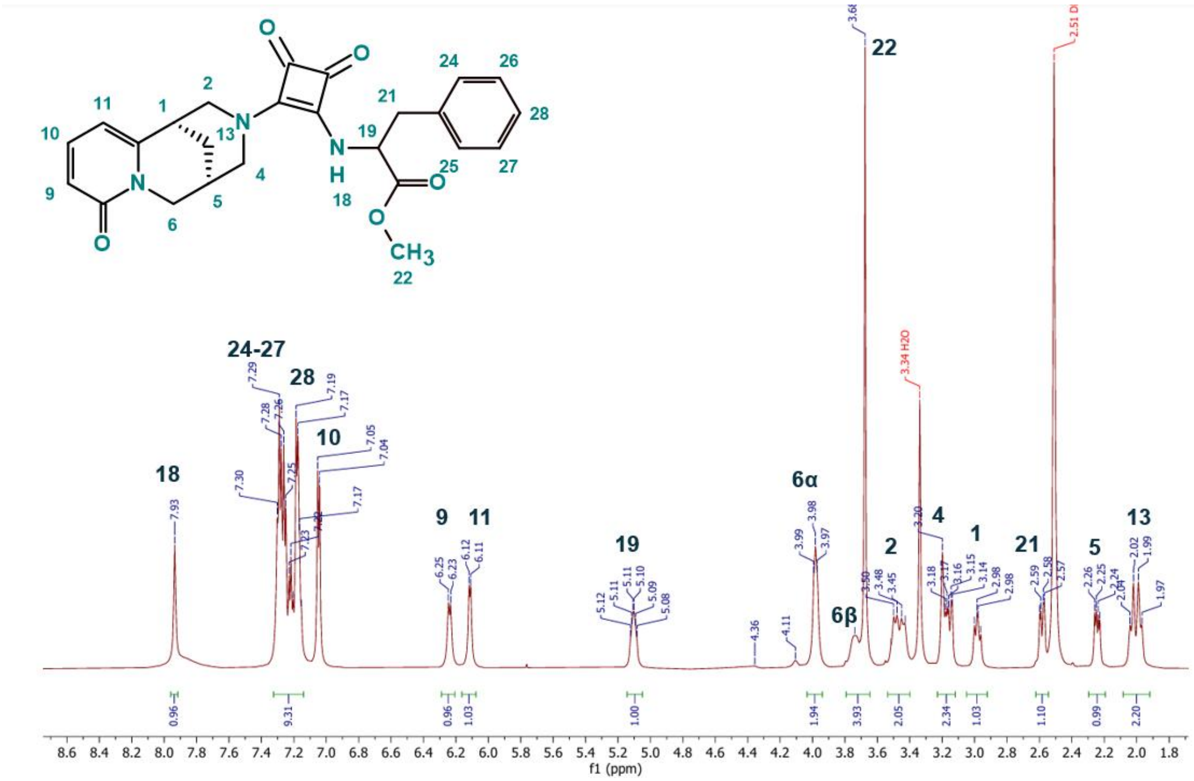

Figure S14. ^{1}H NMR spectrum of **5** (600 MHz, DMSO-d$_6$).

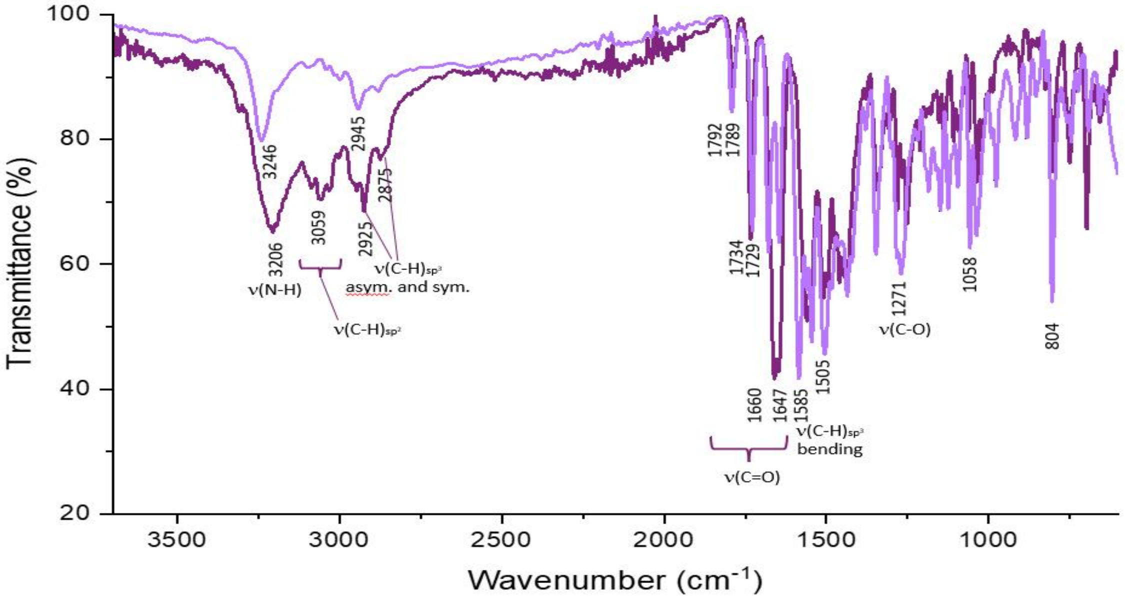

F

igure S15. The overlay of FT-IR spectra for **4** (violet purple line) and **5** (maroon line) in the frequency range 3700 - 500 cm^{-1}.

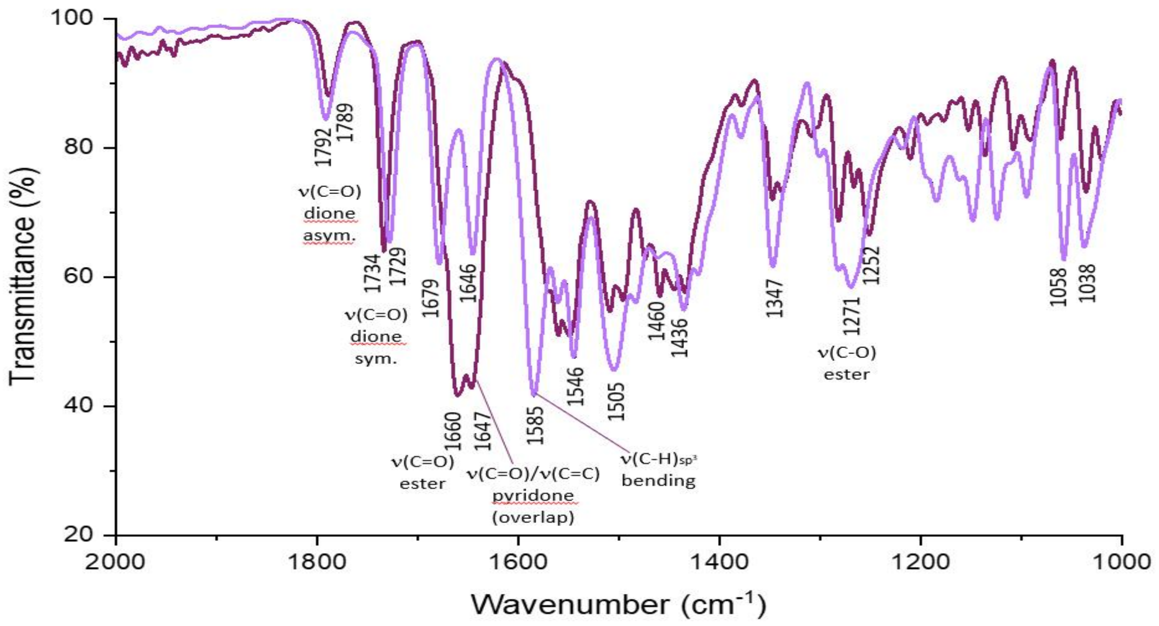

Figure S16. The overlay of FT-IR spectra for **4** (purple line) and **5** (maroon line) in the frequency range 2000-1000 cm^{-1}.

Table S1. Full optimized geometrical parameters (Å, º) for **3**.

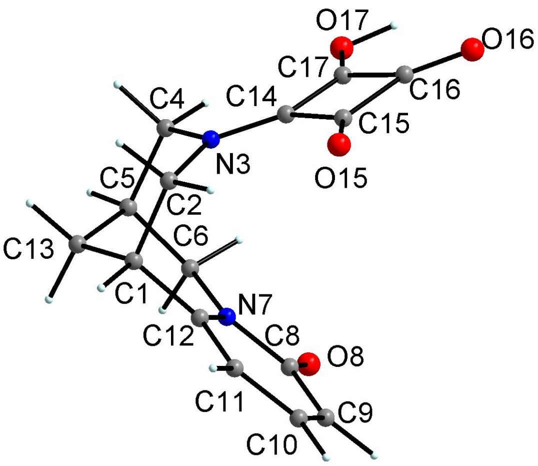

Bond length (Å)					
C1–C2	1.549	C2–N3	1.465	N3–C4	1.465
C4–C5	1.541	C5–C6	1.533	C6–N7	1.479
N7–C8	1.430	C8–O8	1.231	C8–C9	1.444
C9–C10	1.365	C10–C11	1.418	C11–C12	1.371
N7–C12	1.376	C12–C1	1.516	C1–C13	1.535
C13–C5	1.535	N3–C14	1.338	C14–C15	1.508
C15–O15	1.211	C15–C16	1.548	C16–O16	1.214
C16–C17	1.469	C17–O17	1.339	C17–C14	1.391
O17–H	0.971				
Angles (º)					
C1–C2–N3	110.28	C2–N3–C4	115.98	N3–C4–C5	111.37
C4–C5–C6	113.38	C5–C6–N7	114.77	C6–N7–C8	112.91
N7–C8–O8	119.05	N7–C8–C9	114.97	C8–C9–C10	121.50
C9–C10–C11	120.63	C10–C11–C12	119.82	C11–C12–C1	121.21
C12–C1–C13	111.05	C1–C13–C5	106.90	C2–N3–C14	121.07
N3–C14–C15	132.33	C14–C15–O15	134.03	C14–C15–C16	87.38
C15–C16–O16	139.05	C15–C16–C17	86.56	C16–C17–O17	132.40
C16–C17–C14	95.07				
Torsion angles (º)					
C1–C2–N3–C4	52.36	C2–N3–C4–C5	-51.03	N3–C4–C5–C6	-68.26
C4–C5–C6–N7	83.69	C5–C6–N7–C8	-173.95	C6–N7–C8–O8	1.82
C6–N7–C8–C9	-177.90	N7–C8–C9–C10	1.93	C8–C9–C10–C11	-0.58
C9–C10–C11–C12	-0.76	C10–C11–C12–C1	-177.14	C11–C12–C1–C2	87.48
C11–C12–C1–C13	-150.95	C12–C1–C2–N3	64.87	C12–C1–C13–C5	-59.79
C13–C5–C4–N3	54.78	C5–C4–N3–C14	128.82	C4–N3–C14–C15	179.16
N3–C14–C15–O15	0.23	C14–C15–C16–O16	179.69	C14–C15–C16–C17	-0.12
C15–C16–C17–O17	-179.75	N3–C14–C17–O17	-0.35	N3–C15–C17–C16	179.77

Dihedral angle between the plane of pyridone ring and square acidic ring = 60.79
Deviation of C13 from the plane of envelope ring (C1,C12,N7,C6,C5) = 0.735

Table S2. Hydrogen-bond geometry (Å, °) in the **3·DMSO** crystal.

D—H···*A*	*D*—H	H···*A*	*D*···*A*	*D*—H···*A*
C21—H21*B*···O16	0.98	2.30	3.161 (5)	146
C1—H1···O1i	1.00	2.50	3.129 (4)	120
C2—H2*A*···O1^{i}	0.99	2.47	3.071 (4)	118
C2—H2*B*···O15	0.99	2.55	3.304 (4)	133
C4—H4*A*···O18	0.99	2.44	3.183 (4)	131
C6—H6*A*···O16^{ii}	0.99	2.50	3.342 (4)	142
O18—H18···O8^{iii}	0.84 (1)	1.63 (2)	2.460 (3)	166 (4)

Symmetry codes: (*i*) *x*+1, *y*, *z*+1; (*ii*) *x*+1, *y*, *z*; (*iii*) −*x*+1, *y*−1/2, −*z*.

Table S3. Hydrogen-bond geometry (Å, °) in the **3.**

D—H···*A*	*D*—H	H··· *A*	*D*···*A*	*D*—H···*A*
O17—H17···O8*A*	0.91 (3)	1.61 (3)	2.510 (2)	170 (3)
O17*A*—H17*A*···O8^{i}	0.86 (3)	1.68 (3)	2.531 (2)	168 (3)
C2—H2*B*···O15	0.99	2.40	3.183 (3)	135
C4—H4*A*···O17	0.99	2.56	3.307 (3)	132
C4—H4*B*···O15^{ii}	0.99	2.29	3.052 (3)	133
C9—H9···O16*A*^{iii}	0.95	2.29	3.198 (3)	160
C2*A*—H2*D*···O15*A*	0.99	2.44	3.222 (3)	136
C4*A*—H4*C*···O15*A*^{iv}	0.99	2.55	3.393 (3)	142
C4*A*—H4*C*···O17*A*	0.99	2.59	3.263 (3)	126
C9*A*—H9*A*···O16^{ii}	0.95	2.57	3.240 (3)	128
C9*A*—H9*A*···O17	0.95	2.58	3.166 (3)	120

Symmetry codes: (*i*) *x*, *y*+1, *z*; (*ii)* *x*+1, *y*, *z*; (*iii)* *x*−1, *y*−1, *z*; (*iv*) *x*−1, *y*, *z*.

Table S4. Full optimized geometrical parameters (Å, º) for **4**.

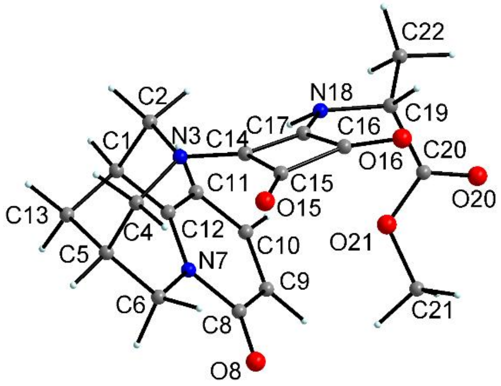

**Bond length (Å)**					
C1–C2	1.553	C2–N3	1.455	N3–C4	1.464
C4–C5	1.542	C5–C6	1.538	C6–N7	1.486
N7–C8	1.429	C8–O8	1.232	C8–C9	1.444
C9–C10	1.364	C10–C11	1.419	C11–C12	1.375
N7–C12	1.372	C12–C1	1.511	C1–C13	1.537
C13–C5	1.536	N3–C14	1.352	C14–C15	1.491
C15–O15	1.215	C15–C16	1.539	C16–O16	1.215
C16–C17	1.487	C17–N18	1.356	N18–C19	1.467
C19–C20	1.540	C20–O20	1.212	C20–O21	1.342
O21–C21	1.445	C19–C22	1.529	N18–H	1.016
**Angles (º)**					
C1–C2–N3	111.43	C2–N3–C4	115.03	N3–C4–C5	109.69
C4–C5–C6	113.00	C5–C6–N7	115.34	C6–N7–C8	113.28
N7–C8–O8	119.23	N7–C8–C9	115.26	C8–C9–C10	121.63
C9–C10–C11	120.35	C10–C11–C12	119.74	C11–C12–C1	121.18
C12–C1–C13	110.53	C1–C13–C5	107.08	C2–N3–C14	124.42
N3–C14–C15	130.67	C14–C15–O15	134.50	C14–C15–C16	87.24
C15–C16–O16	138.01	C15–C16–C17	87.26	C16–C17–N18	131.44
C16–C17–C14	92.77	C17–N18–C19	119.55	N18–C19–C20	114.42
N18–C19–C22	110.69	C22–C19–C20	110.56	C19–C20–O20	122.75
C19–C20–O21	112.64	O20–C20–O21	124.59	C20–O21–C21	116.07
**Torsion angles (º)**					
C1–C2–N3–C4	53.18	C2–N3–C4–C5	55.33	N3–C4–C5–C6	63.20
C4–C5–C6–N7	-88.03	C5–C6–N7–C8	177.00	C6–N7–C8–O8	-3.85
C6–N7–C8–C9	175.92	N7–C8–C9–C10	1.17	C8–C9–C10–C11	0.02
C9–C10–C11–C12	-1.03	C10–C11–C12–C	178.50	C11–C12–C1–C2	-90.70
C11–C12–C1–C13	147.92	C12–C1–C2–N3	-67.13	C12–C1–C13–C5	62.05
C13–C5–C4–N3	-59.61	C5–C4–N3–C14	-119.24	C4–N3–C14–C15	-21.28
N3–C14–C15–O15	5.87	C14–C15–C16–O16	175.74	C14–C15–C16–C17	-3.29
C15–C16–C17–N18	-175.60	N3–C14–C17–N18	-3.56	N3–C15–C17–C16	177.41
O16–C16–C17–N18	5.32	C14–C17–N18–C19	-168.70	C17–N18–C19–C22	-158.02
C17–N18–C19–C20	76.27	N18–C19–C20–O20	168.04	N18–C19–C20–O21	-13.56
C19–C20–O21–C21	-174.73	O20–C20–O21–C21	3.64		

Dihedral angle between the plane of pyridone ring and square acidic ring = 58.00

Deviation of C13 from the plane of envelope ring (C1,C12,N7,C6,C5) = 0.741

Table S5. Hydrogen-bond geometry (Å, º) in the **4.**

D—H···*A*	*D*—H	H···*A*	*D*···*A*	*D*—H···*A*
N18—H18···O8^{i}	0.86	2.11	2.961 (3)	168
C2—H2*A*···O8^{i}	0.97	2.32	3.225 (3)	154
C2—H2*A*···N18	0.97	2.68	3.422 (3)	133
C4—H4*A*···O15^{ii}	0.97	2.59	3.442 (4)	146
C4—H4*B*···O15	0.97	2.44	3.211 (4)	136
C5—H5···O20^{iii}	0.98	2.55	3.299 (3)	134

Symmetry codes: (*i*) *x*+1, *y*, *z*; (*ii*) *x*+1/2, −*y*+1/2, −*z*+1; (*iii*) −*x*+1/2, −*y*+1, *z*+1/2.

Table S6. Full optimized geometrical parameters (Å, º) for **5**.

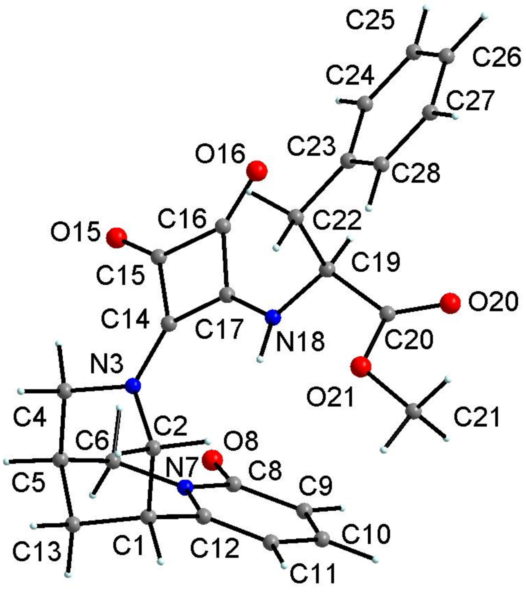

Bond length (Å)					
C1–C2	1.550	C2–N3	1.457	N3–C4	1.469
C4–C5	1.538	C5–C6	1.535	C6–N7	1.481
N7–C8	1.431	C8–O8	1.230	C8–C9	1.445
C9–C10	1.364		C10–C11		1.419
C11–C12		1.372	N7–C12		1.373
C12–C1		1.515	C1–C13		1.535
C13–C5		1.534	N3–C14		1.348
C14–C15		1.492	C15–O15		1.215
C15–C16		1.534	C16–O16		1.217
C16–C17		1.486	C17–N18		1.353
N18–C19		1.468	C19–C20		1.524
C20–O20		1.206	C20–O21		1.360
O21–C21		1.440	C19–C22		1.556
C22–C23		1.512	C23–C24		1.403
C24–C25		1.394	C25–C26		1.397
C26–C27		1.395	C27–C28		1.396
C28–C23		1.399	N18–H		1.011

Angles (º)					
C1–C2–N3	110.90	C2–N3–C4	115.85	N3–C4–C5	111.33
C4–C5–C6	113.32	C5–C6–N7	114.89	C6–N7–C8	112.93
N7–C8–O8	119.12	N7–C8–C9	114.98	C8–C9–C10	121.55
C9–C10–C11	120.54	C10–C11–C12	119.77	C11–C12–C1	121.12
C12–C1–C13	110.80	C1–C13–C5	106.73	C2–N3–C14	123.58
N3–C14–C15	130.74	C14–C15–O15	134.52	C14–C15–C16	87.54
C15–C16–O16	137.79	C15–C16–C17	87.54	C16–C17–N18	130.80
C16–C17–C14	92.72	C17–N18–C19	120.90	N18–C19–C20	108.83
N18–C19–C22	112.49	C22–C19–C20	111.29	C19–C20–O20	125.69
C19–C20–O21	110.59	O20–C20–O21	123.70	C20–O21–C21	115.19
C22–C23–C24	119.83	C23–C24–C25	120.44	C24–C25–C26	120.25
C25–C26–C27	119.80	C26–C27–C28	120.01	C27–C28–C23	120.71

Torsion angles (º)					
C1–C2–N3–C4	51.03	C2–N3–C4–C5	-51.27	N3–C4–C5–C6	-67.19
C4–C5–C6–N7	86.31	C5–C6–N7–C8	-176.98	C6–N7–C8–O8	0.91
C6–N7–C8–C9	-178.84	N7–C8–C9–C10	2.24	C8–C9–C10–C11	-0.59
C9–C10–C11–C12	-0.91	C10–C11–C12–C1	-177.95	C11–C12–C1–C2	88.50
C11–C12–C1–C13	-149.41	C12–C1–C2–N3	66.49	C12–C1–C13–C5	-60.86
C13–C5–C4–N3	56.14	C5–C4–N3–C14	136.08	C4–N3–C14–C15	-12.54
N3–C14–C15–O15	2.07	C14–C15–C16–O16	179.94	C14–C15–C16–C17	-0.55
C15–C16–C17–N18	179.35	N3–C14–C17–N18	0.02	N3–C15–C17–C16	178.66
O16–C16–C17–N18	-1.12	C14–C17–N18–C19	-176.85	C17–N18–C19–C22	78.60
C17–N18–C19–C20	-157.60	N18–C19–C20–O20	134.46	N18–C19–C20–O21	-47.28
C19–C20–O21–C21	-178.74	O20–C20–O21–C21	-0.44	N18–C19–C22–C23	-172.37
C20–C19–C22–C23	65.20	C19–C22–C23–C24	74.52	C22–C23–C24–C25	-179.22
C23–C24–C25–C26	-0.13	C24–C25–C26–C27	0.01	C25–C26–C27–C28	0.10
C26–C26–C28–C23	-0.10	C27–C28–C23–C24	-0.02	C28–C23–C24–C25	0.13

Dihedral angle between the plane of pyridone ring and sqaric acid ring = 68.90

Deviation of C13 from the plane of envelope ring (C1,C12,N7,C6,C5) = 0.744

Table S7. Hydrogen-bond geometry (Å, º) in the **5.**

D—H···A	D—H	H···A	D···A	D—H···A
N18—H18···O15^{i}	0.86	2.16	2.946 (3)	152
C2—H2B···O15^{i}	0.97	2.66	3.573 (4)	157
C4—H4A···O15	0.97	2.42	3.191 (4)	136
C5—H5···O20^{ii}	0.98	2.59	3.435 (4)	145
C11—H11···O8^{i}	0.93	2.50	3.248 (4)	138
C19—H19···O16	0.98	2.36	3.151 (3)	138
C21—H21C···O8^{i}	0.96	2.39	3.318 (5)	163
C22—H22B···O16^{i}	0.97	2.64	3.475 (4)	145

Symmetry codes: (i) x−1, y, z; (ii) x+1, y−1, z.

Table S8. Full optimized geometrical parameters (Å, º) for **6**. (6_OMe)

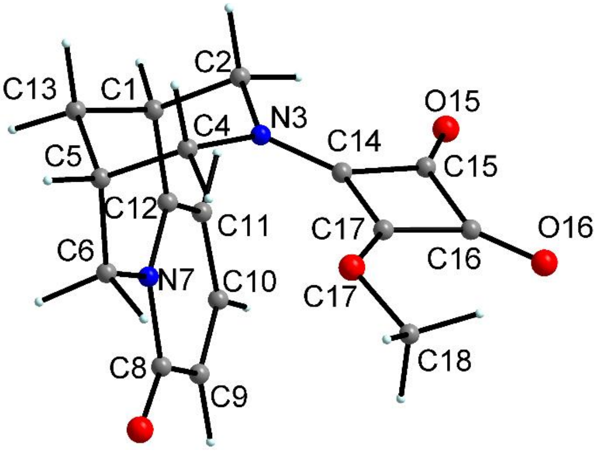

Bond length (Å)					
C1–C2	1.549	C2–N3	1.464	N3–C4	1.465
C4–C5	1.541	C5–C6	1.533	C6–N7	1.479
N7–C8	1.429	C8–O8	1.232	C8–C9	1.443
C9–C10	1.365	C10–C11	1.418	C11–C12	1.371
N7–C12	1.376	C12–C1	1.516	C1–C13	1.536
C13–C5	1.535	N3–C14	1.339	C14–C15	1.500
C15–O15	1.213	C15–C16	1.545	C16–O16	1.214
C16–C17	1.479	C17–O17	1.332	C17–C14	1.396
O17–C18	1.447				
Angles (º)					
C1–C2–N3	110.19	C2–N3–C4	115.94	N3–C4–C5	111.29
C4–C5–C6	113.25	C5–C6–N7	114.78	C6–N7–C8	112.89
N7–C8–O8	119.06	N7–C8–C9	114.98	C8–C9–C10	121.49
C9–C10–C11	120.63	C10–C11–C12	119.82	C11–C12–C1	121.25
C12–C1–C13	111.07	C1–C13–C5	106.92	C2–N3–C14	121.00
N3–C14–C15	132.33	C14–C15–O15	135.26	C14–C15–C16	86.51
C15–C16–O16	137.35	C15–C16–C17	86.51	C16–C17–O17	135.26
C16–C17–C14	94.38	C17–C14–C15	91.34	C17–O17–C18	115.80
Torsion angles (º)					
C1–C2–N3–C4	-52.67	C2–N3–C4–C5	51.29	N3–C4–C5–C6	68.15
C4–C5–C6–N7	-83.64	C5–C6–N7–C8	173.70	C6–N7–C8–O8	-1.91
C6–N7–C8–C9	177.88	N7–C8–C9–C10	-1.78	C8–C9–C10–C11	0.56
C9–C10–C11–C12	0.70	C10–C11–C12–C1	176.97	C11–C12–C1–C2	-87.45
C11–C12–C1–C13	151.03	C12–C1–C2–N3	-64.73	C12–C1–C13–C5	59.78
C13–C5–C4–N3	57.89	C5–C4–N3–C14	-128.45	C4–N3–C14–C15	-178.99
N3–C14–C15–O15	-0.43	C14–C15–C16–O16	-178.35	C14–C15–C16–C17	0.74
C15–C16–C17–O17	-179.37	N3–C14–C17–O17	-0.45	C16–C17–O17–C18	-10.33

Dihedral angle between the plane of pyridone ring and square acidic ring = 60.09
Deviation of C13 from the plane of envelope ring (C1,C12,N7,C6,C5) = 0.734

Table S9. Hydrogen-bond geometry (Å, °) in the co-crystal **6.**

D—H···A	D—H	H···A	D···A	D—H···A
O15—H15···O8*i*	0.91 (5)	1.59 (5)	2.506 (3)	175 (4)
C2—H2A···O15	0.97	2.45	3.207 (4)	135
C2—H2B···O8*ii*	0.97	2.51	3.444 (4)	162
C4—H4A···O16*iii*	0.97	2.48	3.432 (4)	169
C4—H4B···O18	0.97	2.56	3.297 (4)	133
C6—H6A···O16A	0.97	2.53	3.211 (4)	127
C9—H9···O8A*i*	0.93	2.38	3.290 (4)	167
C2A—H21···O15A	0.97	2.51	3.261 (4)	134
C4A—H41···O16iv	0.97	2.65	3.518 (4)	150
C4A—H42···O18A	0.97	2.46	3.201 (4)	133

Symmetry codes: (*i*) −x, y+1/2, −z+1; (*ii*) −x+1, y+1/2, −z+1; (*iii*) x+1, y, z; (*iv*) −x, y−1/2, −z.

Table S10. Full optimized geometrical parameters (Å, º) for **7**. (7_OEt)

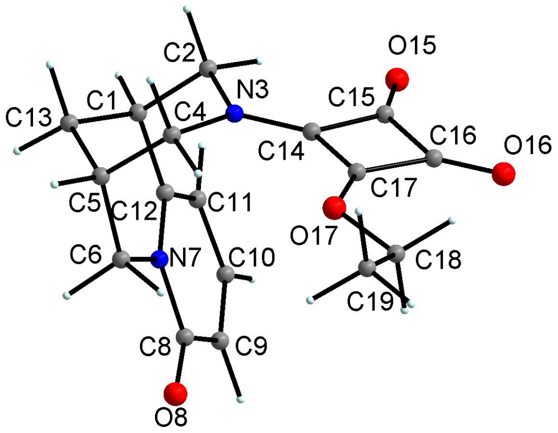

Bond length (Å)					
C1–C2	1.549	C2–N3	1.464	N3–C4	1.464
C4–C5	1.541	C5–C6	1.532	C6–N7	1.479
N7–C8	1.429	C8–O8	1.232	C8–C9	1.443
C9–C10	1.365	C10–C11	1.418	C11–C12	1.371
N7–C12	1.376	C12–C1	1.516	C1–C13	1.536
C13–C5	1.535	N3–C14	1.339	C14–C15	1.499
C15–O15	1.213	C15–C16	1.544	C16–O16	1.214
C16–C17	1.480	C17–O17	1.331	C17–C14	1.396
O17–C18	1.460	C18–C19	1.514		
**Angles (º)**					
C1–C2–N3	110.21	C2–N3–C4	115.96	N3–C4–C5	111.31
C4–C5–C6	113.25	C5–C6–N7	114.77	C6–N7–C8	112.89
N7–C8–O8	119.06	N7–C8–C9	114.99	C8–C9–C10	121.49
C9–C10–C11	120.63	C10–C11–C12	119.82	C11–C12–C1	121.24
C12–C1–C13	111.03	C1–C13–C5	106.91	C2–N3–C14	121.93
N3–C14–C15	132.28	C14–C15–O15	134.26	C14–C15–C16	87.75
C15–C16–O16	137.31	C15–C16–C17	86.54	C16–C17–O17	135.30
C16–C17–C14	94.28	C17–C14–C15	91.42	C17–O17–C18	116.90
O17–C18–C19	107.52				
**Torsion angles (º)**					
C1–C2–N3–C4	-52.68	C2–N3–C4–C5	51.13	N3–C4–C5–C6	68.34
C4–C5–C6–N7	-83.71	C5–C6–N7–C8	173.83	C6–N7–C8–O8	-1.88
C6–N7–C8–C9	177.89	N7–C8–C9–C10	-1.80	C8–C9–C10–C11	0.60
C9–C10–C11–C12	0.70	C10–C11–C12–C1	177.13	C11–C12–C1–C2	-87.55
C11–C12–C1–C13	151.95	C12–C1–C2–N3	-64.61	C12–C1–C13–C5	59.80
C13–C5–C4–N3	-54.66	C5–C4–N3–C14	-130.47	C4–N3–C14–C15	-178.06
N3–C14–C15–O15	-0.41	C14–C15–C16–O16	-178.48	C14–C15–C16–C17	0.64
C15–C16–C17–O17	-179.61	N3–C14–C17–O17	-0.21	C16–C17–O17–C18	-8.45
C17–O17–C18–C19	163.34				

Dihedral angle between the plane of pyridone ring and square acidic ring = 62.30

Deviation of C13 from the plane of envelope ring (C1,C12,N7,C6,C5) = 0.735

Table S11. Hydrogen-bond geometry (Å, °) in the co-crystal **7.**

D—H···A	D—H	H···A	D···A	D—H···A
O15A—H15A···O8A^{i}	0.82	1.71	2.512 (4)	166
C2—H2A···O15	0.97	2.53	3.273 (5)	133
C4—H4B···O17	0.97	2.44	3.192 (5)	134
C18—H18B···O16	0.97	2.43	3.231 (8)	140
C2A—H2AA···O15A	0.97	2.44	3.197 (5)	135
C2A—H2AB···O8A^{ii}	0.97	2.52	3.463 (5)	165
C4A—H4AA···O16A^{iii}	0.97	2.58	3.543 (5)	172
C4A—H4AB···O17A	0.97	2.59	3.309 (5)	131
C6A—H6AA···O16	0.97	2.61	3.265 (5)	125
C9A—H9A···O8^{i}	0.93	2.46	3.374 (5)	166

Symmetry codes: (i) −x, y+1/2, −z+1; (ii) −x+1, y+1/2, −z+1; (iii) x+1, y, z.
