# Supplementary material for: Functionalized Cytisine Squaramides: Synthesis, Structural Elucidation, and Co-Crystallization
Source: Molecules. 2026 Jun 4;31(11):1961. doi: 10.3390/molecules31111961 (PMC13257630; doi:10.3390/molecules31111961)

checkCIF/PLATON report

Structure factors have been supplied for datablock(s) 3dmso

THIS REPORT IS FOR GUIDANCE ONLY. IF USED AS PART OF A REVIEW PROCEDURE FOR PUBLICATION, IT SHOULD NOT REPLACE THE EXPERTISE OF AN EXPERIENCED CRYSTALLOGRAPHIC REFEREE.

No syntax errors found. CIF dictionary Interpreting this report

Datablock: 3dmso

Bond precision:	C-C = 0.0036 A		Wavelength=0.71073
Cell:	a=8.5773(4)	b=8.4952(5)	c=11.7978(6)
	alpha=90	beta=101.924(5)	gamma=90
Temperature:	100 K		

	Calculated	Reported
Volume	841.11(8)	841.11(8)
Space group	P 21	P 21
Hall group	P 2yb	P 2yb
Moiety formula	C15 H14 N2 O4, C2 H6 O S	C15 H14 N2 O4, C2 H6 O S
Sum formula	C17 H20 N2 O5 S	C17 H20 N2 O5 S
Mr	364.41	364.41
Dx,g cm-3	1.439	1.439
Z	2	2
Mu (mm-1)	0.224	0.224
F000	384.0	384.0
F000’	384.43	
h,k,lmax	11,11,16	11,11,16
Nref	4611[2451]	4041
Tmin,Tmax	0.930,0.972	0.957,1.000
Tmin’	0.930	

Correction method= # Reported T Limits: Tmin=0.957 Tmax=1.000
AbsCorr = MULTI-SCAN

Data completeness= 1.65/0.88   Theta(max)= 29.306

R(reflections)= 0.0389(3537)   wR2(reflections)= 0.0845(4041)

S = 1.002   Npar= 241

The following ALERTS were generated. Each ALERT has the format
test-name_ALERT_alert-type_alert-level.
Click on the hyperlinks for more details of the test.

Alert level C

```
PLAT243_ALERT_4_C High MinorResAtom  Ueq as Compared to Neighbours        C22 Check
```

Alert level G

```
PLAT002_ALERT_2_G Number of Distance or Angle Restraints on AtSite          2 Note
PLAT172_ALERT_4_G The CIF-Embedded .res File Contains DFIX Records          1 Report
PLAT302_ALERT_4_G Anion/Solvent/Minor-Residue Disorder (Resd    2)        25% Note
PLAT790_ALERT_4_G Centre of Gravity not Within Unit-Cell: Resd.  #          2 Note
              C2 H6 O S
PLAT791_ALERT_4_G Model has Chirality at C1         (Sohncke SpGr)          R Verify
PLAT791_ALERT_4_G Model has Chirality at C5         (Sohncke SpGr)          R Verify
PLAT860_ALERT_3_G Number of Least-Squares Restraints ............           2 Note
PLAT899_ALERT_4_G SHELXL2018 is Outdated   and Succeeded by SHELXL     2019/3 Note
PLAT910_ALERT_3_G Missing FCF Reflection(s) Below Theta(Min)[Deg]=       2.69 Note
                1  0  0,   0  0  1,
PLAT912_ALERT_4_G Missing # of FCF Reflections Above STh/L=  0.600        177 Note
PLAT941_ALERT_3_G Average HKL Measurement Multiplicity ..........         4.9 Low
PLAT965_ALERT_2_G The SHELXL WEIGHT Optimisation has not Converged     Please Check
PLAT969_ALERT_5_G The 'Henn et al.' R-Factor-gap value ..........       2.892 Note
              Predicted wR2: Based on SigI**2  2.92 or SHELX Weight  8.43 Note
PLAT978_ALERT_2_G Number C-C Bonds with Positive Residual Density.          4 Info
```

---

0 **ALERT level A** = Most likely a serious problem - resolve or explain
0 **ALERT level B** = A potentially serious problem, consider carefully
1 **ALERT level C** = Check. Ensure it is not caused by an omission or oversight
14 **ALERT level G** = General information/check it is not something unexpected

0 ALERT type 1 CIF construction/syntax error, inconsistent or missing data
3 ALERT type 2 Indicator that the structure model may be wrong or deficient
3 ALERT type 3 Indicator that the structure quality may be low
8 ALERT type 4 Improvement, methodology, query or suggestion
1 ALERT type 5 Informative message, check

It is advisable to attempt to resolve as many as possible of the alerts in all categories. Often the minor alerts point to easily fixed oversights, errors and omissions in your CIF or refinement strategy, so attention to these fine details can be worthwhile. It is up to the individual to critically assess their own results and, if necessary, seek expert advice.

PLATON version of 23/04/2026; check.def file version of 30/03/2026

duplicate check

No duplication found

Datablock 3dmso - ellipsoid plot

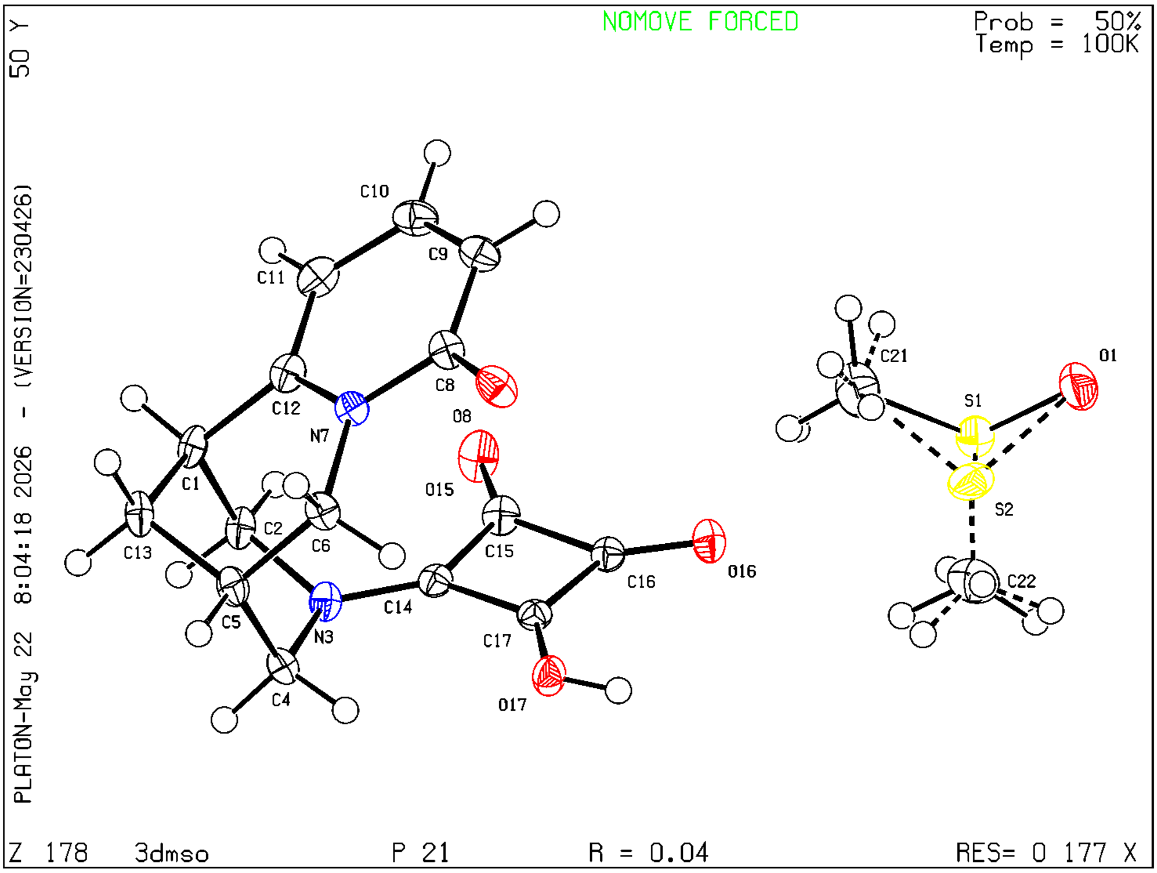

Supplement: Supplementary file 1 [file molecules-31-01961-s001.zip › checkcif_3_DMSO.pdf]
