# Supplementary material for: Functionalized Cytisine Squaramides: Synthesis, Structural Elucidation, and Co-Crystallization
Source: Molecules. 2026 Jun 4;31(11):1961. doi: 10.3390/molecules31111961 (PMC13257630; doi:10.3390/molecules31111961)

checkCIF/PLATON report

Structure factors have been supplied for datablock(s) AP_cytSqAlaOMe_RT

THIS REPORT IS FOR GUIDANCE ONLY. IF USED AS PART OF A REVIEW PROCEDURE FOR PUBLICATION, IT SHOULD NOT REPLACE THE EXPERTISE OF AN EXPERIENCED CRYSTALLOGRAPHIC REFEREE.

No syntax errors found. CIF dictionary Interpreting this report

Datablock: AP_cytSqAlaOMe_RT

```
Bond precision:   C-C = 0.0039 A          Wavelength=0.71073

Cell:              a=7.1836(3)      b=11.0542(5)       c=22.4305(8)
                   alpha=90         beta=90            gamma=90
Temperature:       295 K
```

	Calculated	Reported
Volume	1781.18(13)	1781.18(13)
Space group	P 21 21 21	P 21 21 21
Hall group	P 2ac 2ab	P 2ac 2ab
Moiety formula	C19 H21 N3 O5	C19 H21 N3 O5
Sum formula	C19 H21 N3 O5	C19 H21 N3 O5
Mr	371.39	371.39
Dx,g cm-3	1.385	1.385
Z	4	4
Mu (mm-1)	0.102	0.102
F000	784.0	784.0
F000'	784.39	
h,k,lmax	9,15,30	9,15,30
Nref	4903[2802]	4598
Tmin,Tmax	0.971,0.979	0.982,1.000
Tmin'	0.969	

```
Correction method= # Reported T Limits: Tmin=0.982 Tmax=1.000
AbsCorr = MULTI-SCAN
```

```
Data completeness= 1.64/0.94        Theta(max)= 29.377

                                                wR2(reflections)=
R(reflections)= 0.0556( 2812)                   0.0929( 4598)

S = 1.002                Npar= 246
```

The following ALERTS were generated. Each ALERT has the format
test-name_ALERT_alert-type_alert-level.
Click on the hyperlinks for more details of the test.

Alert level C

```
STRVA01_ALERT_4_C           Flack test results are ambiguous.
           From the CIF: _refine_ls_abs_structure_Flack    0.400
           From the CIF: _refine_ls_abs_structure_Flack_su    0.700
PLAT230_ALERT_2_C Hirshfeld Test Diff for    O8       --C8       .        5.5 s.u.
PLAT906_ALERT_3_C Large K Value in the Analysis of Variance ......      3.659 Check
```

Alert level G

```
PLAT007_ALERT_5_G Number of Unrefined Donor-H Atoms .............          1 Report
              H18
PLAT032_ALERT_4_G Std. Uncertainty on Flack Parameter Value High .      0.700 Report
PLAT480_ALERT_4_G Long H...A H-Bond Reported H2A      ..N18      .       2.68 Ang.
PLAT791_ALERT_4_G Model has Chirality at C1         (Sohncke SpGr)          S Verify
PLAT791_ALERT_4_G Model has Chirality at C5         (Sohncke SpGr)          R Verify
PLAT791_ALERT_4_G Model has Chirality at C19        (Sohncke SpGr)          R Verify
PLAT899_ALERT_4_G SHELXL2018 is Outdated   and Succeeded by SHELXL     2019/3 Note
PLAT910_ALERT_3_G Missing FCF Reflection(s) Below Theta(Min)[Deg]=       2.59 Note
                0  1  1,   0  0  2,
PLAT912_ALERT_4_G Missing # of FCF Reflections Above STh/L=  0.600        105 Note
PLAT965_ALERT_2_G The SHELXL WEIGHT Optimisation has not Converged     Please Check
PLAT969_ALERT_5_G The 'Henn et al.' R-Factor-gap value ..........      3.266 Note
              Predicted wR2: Based on SigI**2  2.84 or SHELX Weight  9.27 Note
PLAT978_ALERT_2_G Number C-C Bonds with Positive Residual Density.          0 Info
```

---

0 **ALERT level A** = Most likely a serious problem - resolve or explain
0 **ALERT level B** = A potentially serious problem, consider carefully
3 **ALERT level C** = Check. Ensure it is not caused by an omission or oversight
12 **ALERT level G** = General information/check it is not something unexpected

0 ALERT type 1 CIF construction/syntax error, inconsistent or missing data
3 ALERT type 2 Indicator that the structure model may be wrong or deficient
2 ALERT type 3 Indicator that the structure quality may be low
8 ALERT type 4 Improvement, methodology, query or suggestion
2 ALERT type 5 Informative message, check

It is advisable to attempt to resolve as many as possible of the alerts in all categories. Often the minor alerts point to easily fixed oversights, errors and omissions in your CIF or refinement strategy, so attention to these fine details can be worthwhile. It is up to the individual to critically assess their own results and, if necessary, seek expert advice.

PLATON version of 23/04/2026; check.def file version of 30/03/2026

---

duplicate check

No duplication found

Datablock AP_cytSqAlaOMe_RT - ellipsoid plot

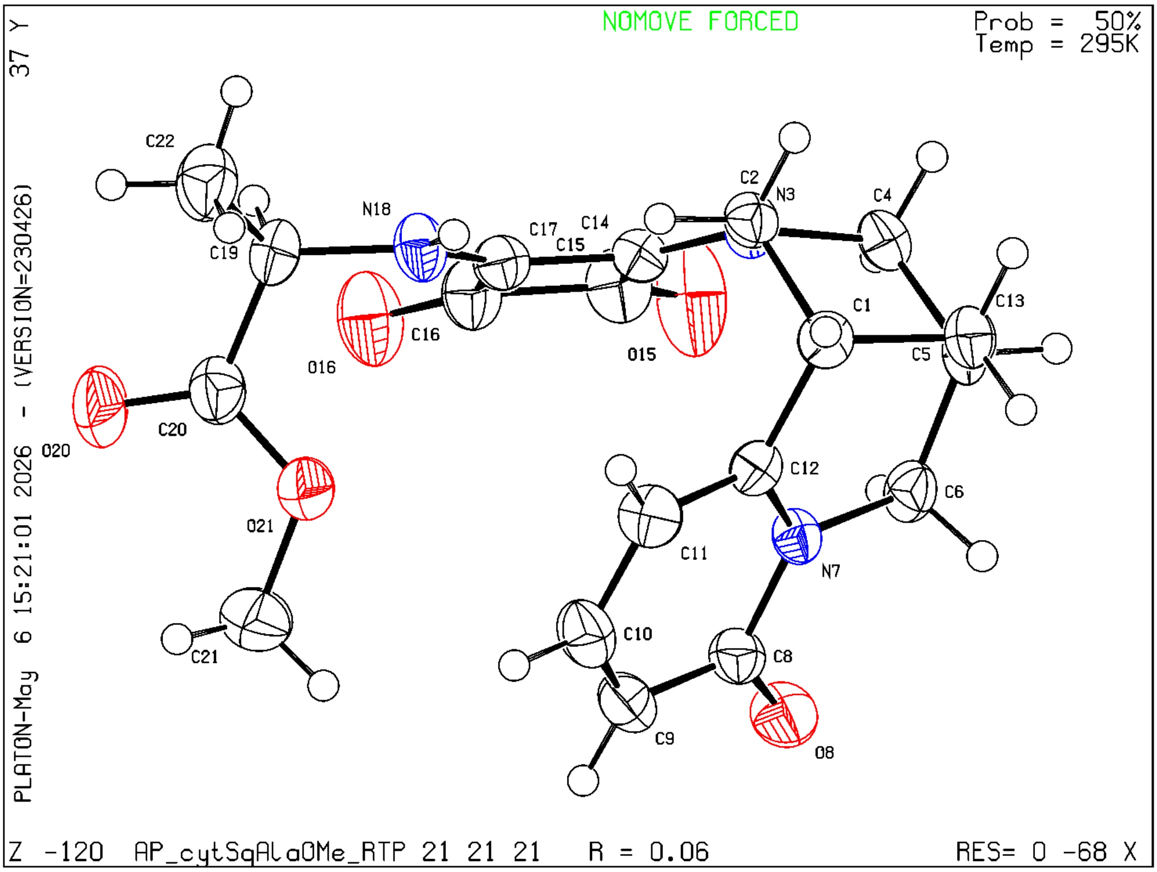

Supplement: Supplementary file 1 [file molecules-31-01961-s001.zip › checkcif_4.pdf]
