# Supplementary material for: Functionalized Cytisine Squaramides: Synthesis, Structural Elucidation, and Co-Crystallization
Source: Molecules. 2026 Jun 4;31(11):1961. doi: 10.3390/molecules31111961 (PMC13257630; doi:10.3390/molecules31111961)

checkCIF/PLATON report

Structure factors have been supplied for datablock(s) AP_cytSqPhAlaOMe_RT

THIS REPORT IS FOR GUIDANCE ONLY. IF USED AS PART OF A REVIEW PROCEDURE FOR PUBLICATION, IT SHOULD NOT REPLACE THE EXPERTISE OF AN EXPERIENCED CRYSTALLOGRAPHIC REFEREE.

No syntax errors found. CIF dictionary Interpreting this report

Datablock: AP_cytSqPhAlaOMe_RT

```
Bond precision:   C-C = 0.0048 A          Wavelength=0.71073

Cell:               a=6.9920(3)      b=9.3258(5)      c=33.231(2)
                    alpha=90         beta=90          gamma=90
Temperature:        295 K

                Calculated                 Reported
Volume          2166.9(2)                  2166.9(2)
Space group     P 21 21 21                 P 21 21 21
Hall group      P 2ac 2ab                  P 2ac 2ab
Moiety formula  C25 H25 N3 O5              C25 H25 N3 O5
Sum formula     C25 H25 N3 O5              C25 H25 N3 O5
Mr              447.48                     447.48
Dx,g cm-3       1.372                      1.372
Z               4                          4
Mu (mm-1)       0.097                      0.097
F000            944.0                      944.0
F000'           944.45
h,k,lmax        9,12,45                    9,12,43
Nref            5956[ 3412]                5257
Tmin,Tmax       0.970,0.979                0.958,1.000
Tmin'           0.969

Correction method= # Reported T Limits: Tmin=0.958 Tmax=1.000
AbsCorr = MULTI-SCAN
```

Data completeness= 1.54/0.88 Theta(max)= 29.349

R(reflections)= 0.0550(3753) wR2(reflections)= 0.1297(5257)

S = 1.046 Npar= 299

The following ALERTS were generated. Each ALERT has the format
test-name_ALERT_alert-type_alert-level.
Click on the hyperlinks for more details of the test.

Alert level C

```
STRVA01_ALERT_4_C           Flack parameter is too small
          From the CIF: _refine_ls_abs_structure_Flack   -0.700
          From the CIF: _refine_ls_abs_structure_Flack_su    0.600
PLAT340_ALERT_3_C Low Bond Precision on  C-C Bonds ..............    0.00478 Ang.
PLAT906_ALERT_3_C Large K Value in the Analysis of Variance ......      2.035 Check
```

Alert level G

```
PLAT007_ALERT_5_G Number of Unrefined Donor-H Atoms .............          1 Report
             H18
PLAT032_ALERT_4_G Std. Uncertainty on Flack Parameter Value High .      0.600 Report
PLAT480_ALERT_4_G Long H...A H-Bond Reported H2B      ..O15      .       2.66 Ang.
PLAT480_ALERT_4_G Long H...A H-Bond Reported H22B     ..O16      .       2.64 Ang.
PLAT791_ALERT_4_G Model has Chirality at C1         (Sohncke SpGr)          R Verify
PLAT791_ALERT_4_G Model has Chirality at C5         (Sohncke SpGr)          R Verify
PLAT791_ALERT_4_G Model has Chirality at C19        (Sohncke SpGr)          S Verify
PLAT899_ALERT_4_G SHELXL2018 is Outdated   and Succeeded by SHELXL     2019/3 Note
PLAT910_ALERT_3_G Missing FCF Reflection(s) Below Theta(Min)[Deg]=       2.50 Note
               0  1  1,   0  0  2,   0  0  4,
PLAT912_ALERT_4_G Missing # of FCF Reflections Above STh/L=  0.600        284 Note
PLAT916_ALERT_2_G Hooft y and Flack x Parameter Values Differ by .       0.20 Check
PLAT941_ALERT_3_G Average HKL Measurement Multiplicity ..........        4.7 Low
PLAT952_ALERT_5_G Calculated (ThMax) and CIF-Reported Lmax  Differ          2 Units
PLAT958_ALERT_1_G Calculated (ThMax) and Actual (FCF) Lmax  Differ          2 Units
PLAT969_ALERT_5_G The 'Henn et al.' R-Factor-gap value ..........      3.742 Note
             Predicted wR2: Based on SigI**2  3.47 or SHELX Weight 12.40 Note
PLAT978_ALERT_2_G Number C-C Bonds with Positive Residual Density.          1 Info
```

---

0 **ALERT level A** = Most likely a serious problem - resolve or explain
0 **ALERT level B** = A potentially serious problem, consider carefully
3 **ALERT level C** = Check. Ensure it is not caused by an omission or oversight
16 **ALERT level G** = General information/check it is not something unexpected

1 ALERT type 1 CIF construction/syntax error, inconsistent or missing data
2 ALERT type 2 Indicator that the structure model may be wrong or deficient
4 ALERT type 3 Indicator that the structure quality may be low
9 ALERT type 4 Improvement, methodology, query or suggestion
3 ALERT type 5 Informative message, check

It is advisable to attempt to resolve as many as possible of the alerts in all categories. Often the minor alerts point to easily fixed oversights, errors and omissions in your CIF or refinement strategy, so attention to these fine details can be worthwhile. It is up to the individual to critically assess their own results and, if necessary, seek expert advice.

PLATON version of 23/04/2026; check.def file version of 30/03/2026

---

duplicate check

No duplication found

Datablock AP_cytSqPhAlaOMe_RT - ellipsoid plot

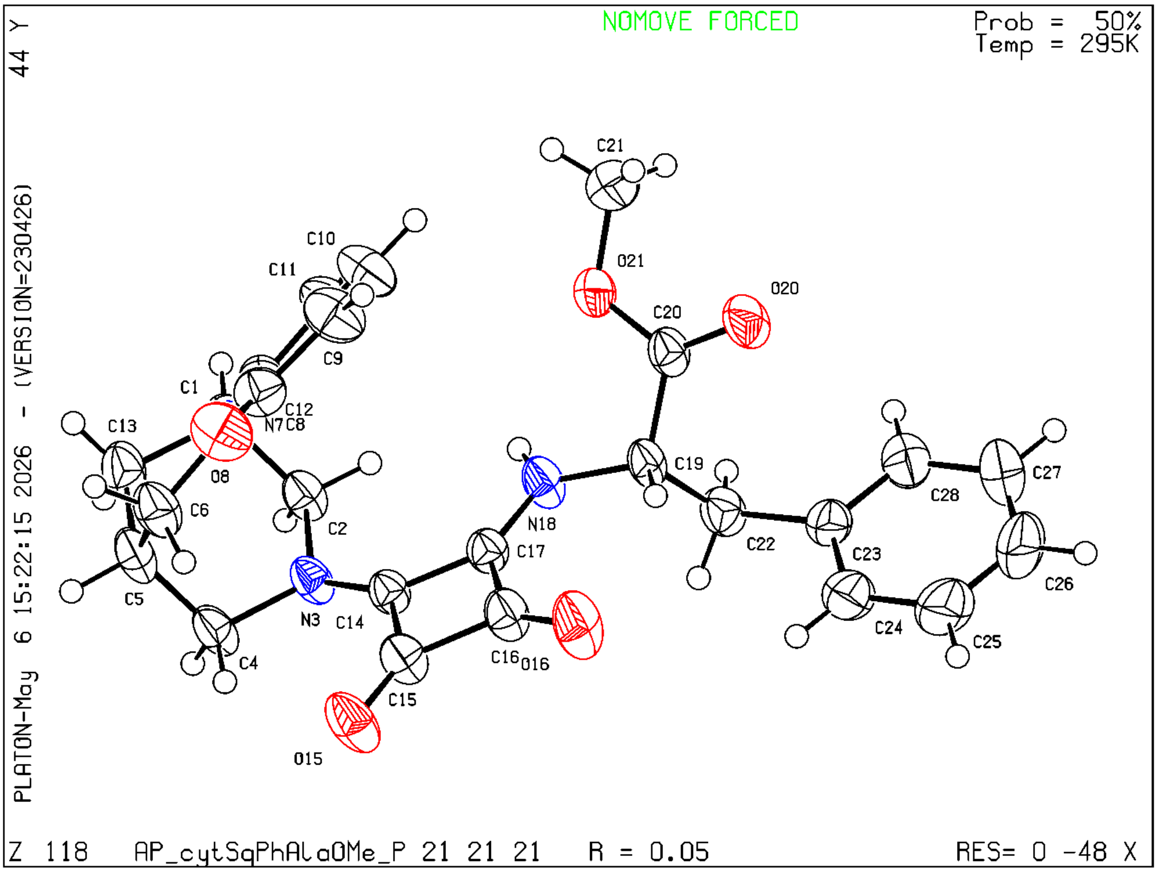

Supplement: Supplementary file 1 [file molecules-31-01961-s001.zip › checkcif_5.pdf]
