# Supplementary material for: Functionalized Cytisine Squaramides: Synthesis, Structural Elucidation, and Co-Crystallization
Source: Molecules. 2026 Jun 4;31(11):1961. doi: 10.3390/molecules31111961 (PMC13257630; doi:10.3390/molecules31111961)

checkCIF/PLATON report

Structure factors have been supplied for datablock(s) AP_cytSqOMe_RT

THIS REPORT IS FOR GUIDANCE ONLY. IF USED AS PART OF A REVIEW PROCEDURE FOR PUBLICATION, IT SHOULD NOT REPLACE THE EXPERTISE OF AN EXPERIENCED CRYSTALLOGRAPHIC REFEREE.

No syntax errors found. CIF dictionary Interpreting this report

Datablock: AP_cytSqOMe_RT

Bond precision:	C-C = 0.0045 A		Wavelength=0.71073
Cell:	a=7.6070(3)	b=14.1981(5)	c=13.1188(5)
	alpha=90	beta=91.282(4)	gamma=90
Temperature:	295 K		

	Calculated	Reported
Volume	1416.54(9)	1416.54(9)
Space group	P 21	P 21
Hall group	P 2yb	P 2yb
Moiety formula	C16 H16 N2 O4, C15 H14 N2 O4	C16 H16 N2 O4, C15 H14 N2 O4
Sum formula	C31 H30 N4 O8	C31 H30 N4 O8
Mr	586.59	586.59
Dx,g cm-3	1.375	1.375
Z	2	2
Mu (mm-1)	0.101	0.101
F000	616.0	616.0
F000'	616.32	
h,k,lmax	10,19,18	10,19,17
Nref	7864[4083]	6756
Tmin,Tmax	0.971,0.988	0.985,1.000
Tmin'	0.968	

Correction method= # Reported T Limits: Tmin=0.985 Tmax=1.000
AbsCorr = MULTI-SCAN

```
Data completeness= 1.65/0.86       Theta(max)= 29.429

                                                        wR2(reflections)=
R(reflections)= 0.0468( 4608)                           0.1016( 6756)

S = 1.004                   Npar= 392
```

```
The following ALERTS were generated. Each ALERT has the format
       test-name_ALERT_alert-type_alert-level.
Click on the hyperlinks for more details of the test.
```

Alert level C

```
PLAT241_ALERT_2_C High  MainResAtom  Ueq as Compared to Neighbours        C6A Check
PLAT241_ALERT_2_C High  MainResAtom  Ueq as Compared to Neighbours         C6 Check
PLAT340_ALERT_3_C Low Bond Precision on  C-C Bonds ...............    0.00446 Ang.
```

Alert level G

```
PLAT032_ALERT_4_G Std. Uncertainty on Flack Parameter Value High .      0.500 Report
PLAT480_ALERT_4_G Long H...A H-Bond Reported H41      ..O16      .       2.65 Ang.
PLAT791_ALERT_4_G Model has Chirality at C1        (Sohncke SpGr)           S Verify
PLAT791_ALERT_4_G Model has Chirality at C1A       (Sohncke SpGr)           S Verify
PLAT791_ALERT_4_G Model has Chirality at C5        (Sohncke SpGr)           R Verify
PLAT791_ALERT_4_G Model has Chirality at C5A       (Sohncke SpGr)           R Verify
PLAT899_ALERT_4_G SHELXL2018 is Outdated   and Succeeded by SHELXL     2019/3 Note
PLAT910_ALERT_3_G Missing FCF Reflection(s) Below Theta(Min)[Deg]=       2.68 Note
                0  0  1,   0  1  1,
PLAT912_ALERT_4_G Missing # of FCF Reflections Above STh/L=  0.600        354 Note
PLAT916_ALERT_2_G Hooft y and Flack x Parameter Values Differ by .       0.10 Check
PLAT969_ALERT_5_G The 'Henn et al.' R-Factor-gap value ..........       4.068 Note
              Predicted wR2: Based on SigI**2  2.50 or SHELX Weight 10.11 Note
PLAT978_ALERT_2_G Number C-C Bonds with Positive Residual Density.          0 Info
```

```
   0 ALERT level A = Most likely a serious problem - resolve or explain
   0 ALERT level B = A potentially serious problem, consider carefully
   3 ALERT level C = Check. Ensure it is not caused by an omission or oversight
  12 ALERT level G = General information/check it is not something unexpected

   0 ALERT type 1 CIF construction/syntax error, inconsistent or missing data
   4 ALERT type 2 Indicator that the structure model may be wrong or deficient
   2 ALERT type 3 Indicator that the structure quality may be low
   8 ALERT type 4 Improvement, methodology, query or suggestion
   1 ALERT type 5 Informative message, check
```

It is advisable to attempt to resolve as many as possible of the alerts in all categories. Often the minor alerts point to easily fixed oversights, errors and omissions in your CIF or refinement strategy, so attention to these fine details can be worthwhile. It is up to the individual to critically assess their own results and, if necessary, seek expert advice.

PLATON version of 23/04/2026; check.def file version of 30/03/2026

duplicate check

No duplication found

Datablock AP_cytSqOMe_RT - ellipsoid plot

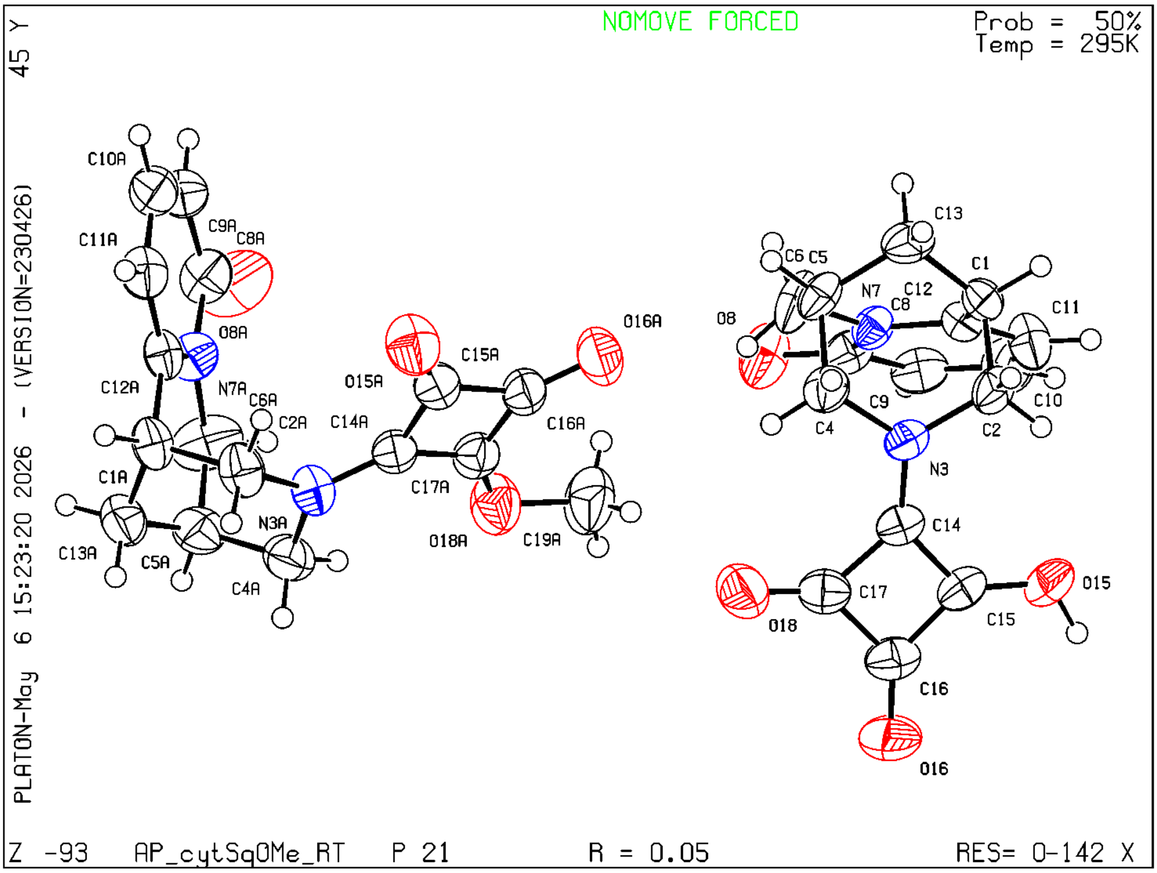

Supplement: Supplementary file 1 [file molecules-31-01961-s001.zip › checkcif_6.pdf]
