# Supplementary material for: Functionalized Cytisine Squaramides: Synthesis, Structural Elucidation, and Co-Crystallization
Source: Molecules. 2026 Jun 4;31(11):1961. doi: 10.3390/molecules31111961 (PMC13257630; doi:10.3390/molecules31111961)

checkCIF/PLATON report

Structure factors have been supplied for datablock(s) AP_R3_EtO_Sq_Cyt_RT

THIS REPORT IS FOR GUIDANCE ONLY. IF USED AS PART OF A REVIEW PROCEDURE FOR PUBLICATION, IT SHOULD NOT REPLACE THE EXPERTISE OF AN EXPERIENCED CRYSTALLOGRAPHIC REFEREE.

No syntax errors found. CIF dictionary Interpreting this report

Datablock: AP_R3_EtO_Sq_Cyt_RT

Bond precision:	C-C = 0.0062 A		Wavelength=0.71073
Cell:	a=7.6761(3)	b=14.3340(4)	c=13.1636(5)
	alpha=90	beta=90.203(3)	gamma=90
Temperature:	295 K		

	Calculated	Reported
Volume	1448.37(9)	1448.37(9)
Space group	P 21	P 21
Hall group	P 2yb	P 2yb
Moiety formula	C17 H18 N2 O4, C15 H14 N2 O4	C17 H18 N2 O4, C15 H14 N2 O4
Sum formula	C32 H32 N4 O8	C32 H32 N4 O8
Mr	600.62	600.61
Dx,g cm-3	1.377	1.377
Z	2	2
Mu (mm-1)	0.100	0.100
F000	632.0	632.0
F000’	632.32	
h,k,lmax	10,19,18	10,19,17
Nref	7929[4114]	7138
Tmin,Tmax	0.985,0.993	0.933,1.000
Tmin’	0.976	

Correction method= # Reported T Limits: Tmin=0.933 Tmax=1.000
AbsCorr = MULTI-SCAN

Data completeness= 1.74/0.90 Theta(max)= 29.303

R(reflections)= 0.0571(4191) wR2(reflections)= 0.1329(7138)

S = 1.001 Npar= 399

The following ALERTS were generated. Each ALERT has the format
test-name_ALERT_alert-type_alert-level.
Click on the hyperlinks for more details of the test.

Alert level C

```
PLAT241_ALERT_2_C High  MainResAtom  Ueq as Compared to Neighbours         C6 Check
PLAT340_ALERT_3_C Low Bond Precision on  C-C Bonds ...............    0.00621 Ang.
PLAT360_ALERT_2_C Short  C(sp3)-C(sp3) Bond  C18       - C19      .      1.35 Ang.
PLAT906_ALERT_3_C Large K Value in the Analysis of Variance ......      3.229 Check
```

Alert level G

```
PLAT007_ALERT_5_G Number of Unrefined Donor-H Atoms .............          1 Report
              H15A
PLAT032_ALERT_4_G Std. Uncertainty on Flack Parameter Value High .      0.500 Report
PLAT480_ALERT_4_G Long H...A H-Bond Reported H6AA     ..O16       .      2.61 Ang.
PLAT720_ALERT_4_G Number of Unusual/Non-Standard Labels ..........          6 Note
              H2AA    H2AB    H4AA    H4AB    H6AA    H6AB
PLAT791_ALERT_4_G Model has Chirality at C1          (Sohncke SpGr)          S Verify
PLAT791_ALERT_4_G Model has Chirality at C1A         (Sohncke SpGr)          S Verify
PLAT791_ALERT_4_G Model has Chirality at C5          (Sohncke SpGr)          R Verify
PLAT791_ALERT_4_G Model has Chirality at C5A         (Sohncke SpGr)          R Verify
PLAT899_ALERT_4_G SHELXL2018 is Outdated   and Succeeded by SHELXL     2019/3 Note
PLAT910_ALERT_3_G Missing FCF Reflection(s) Below Theta(Min)[Deg]=       2.65 Note
                0  0  1,   0  1  1,
PLAT912_ALERT_4_G Missing # of FCF Reflections Above STh/L=  0.600        222 Note
PLAT965_ALERT_2_G The SHELXL WEIGHT Optimisation has not Converged     Please Check
PLAT969_ALERT_5_G The 'Henn et al.' R-Factor-gap value ...........      4.022 Note
              Predicted wR2: Based on SigI**2  3.30 or SHELX Weight 13.27 Note
PLAT978_ALERT_2_G Number C-C Bonds with Positive Residual Density.          0 Info
```

---

0 **ALERT level A** = Most likely a serious problem - resolve or explain
0 **ALERT level B** = A potentially serious problem, consider carefully
4 **ALERT level C** = Check. Ensure it is not caused by an omission or oversight
14 **ALERT level G** = General information/check it is not something unexpected

0 ALERT type 1 CIF construction/syntax error, inconsistent or missing data
4 ALERT type 2 Indicator that the structure model may be wrong or deficient
3 ALERT type 3 Indicator that the structure quality may be low
9 ALERT type 4 Improvement, methodology, query or suggestion
2 ALERT type 5 Informative message, check

It is advisable to attempt to resolve as many as possible of the alerts in all categories. Often the minor alerts point to easily fixed oversights, errors and omissions in your CIF or refinement strategy, so attention to these fine details can be worthwhile. It is up to the individual to critically assess their own results and, if necessary, seek expert advice.

PLATON version of 23/04/2026; check.def file version of 30/03/2026

---

duplicate check

No duplication found

Datablock AP_R3_EtO_Sq_Cyt_RT - ellipsoid plot

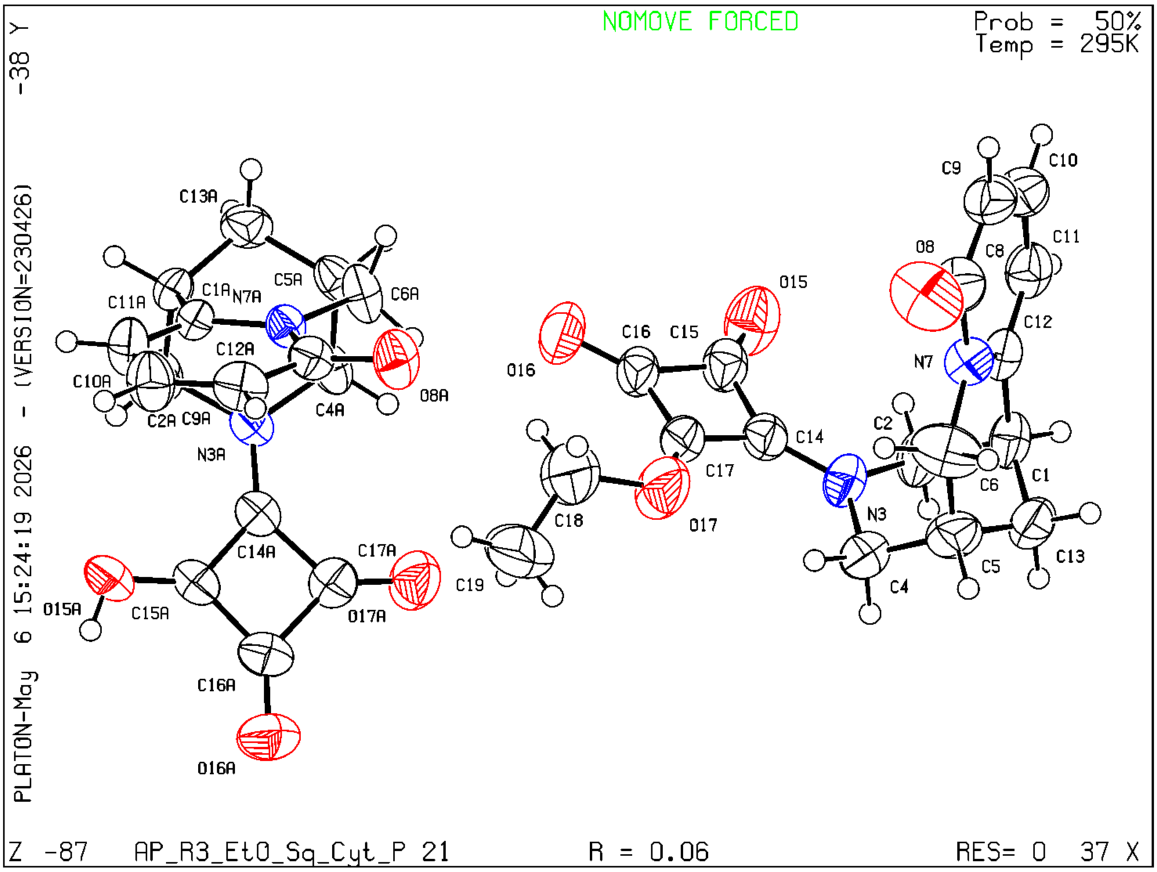

Supplement: Supplementary file 1 [file molecules-31-01961-s001.zip › checkcif_7.pdf]
